# Investigation of the electrophilic reactivity of the biologically active marine sesquiterpenoid onchidal and model compounds

**DOI:** 10.3762/bjoc.14.197

**Published:** 2018-08-24

**Authors:** Melissa M Cadelis, Brent R Copp

**Affiliations:** 1 School of Chemical Sciences, University of Auckland, Private Bag 92019, Auckland 1142, New Zealand

**Keywords:** dialdehyde, lysozyme, mollusc, onchidal, pyrrole

## Abstract

The structure of the sesquiterpene onchidal (**6**), a component of the defensive secretion of the shell-less mollusc *Onchidella binneyi*, contains a masked α,β-unsaturated 1,4-dialdehyde moiety, the presence of which has been proposed to be the cause of the feeding deterrent activity exhibited by the mollusc. We have found onchidal acts as an electrophile, reacting rapidly with the model nucleophile *n*-pentylamine forming diastereomeric aminated pyrrole adducts. Somewhat surprisingly, no reaction was observed between onchidal and *n*-pentanethiol. Structurally simplified *n*-pentyl **11**–**13** and cyclohexylmethyl **15**–**17** analogues of onchidal were prepared and demonstrated similar amine-selective reactivity. Onchidal and analogues reacted with the model protein lysozyme, forming covalent adducts and leading to protein cross-linking. These results provide preliminary evidence supporting the molecular mechanism of biological activity exhibited by onchidal.

## Introduction

**Figure 1:**
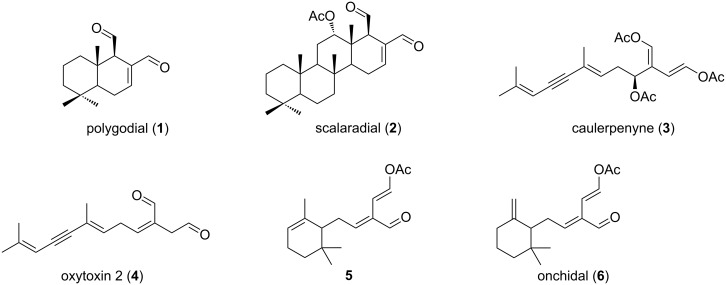
Masked and unmasked 1,4-dialdehyde natural products **1**–**6**.

More than 80 terpenoid natural products containing the 1,4-dialdehyde moiety have been isolated from sources such as fungi, algae, sponges and molluscs [1]. Many of these natural products exhibit biological activity, ranging from anti-inflammatory to antimicrobial and antifeedant activities [1]. The prototypical examples polygodial (**1**) and scalaradial (**2**, Figure 1) both exhibit antifeedant activity against worms and fish [1–2], with recent studies also showing that **1** is a potential lead as a marine antifouling agent [3].

The ichthyotoxic masked dialdehyde caulerpenyne (**3**), a major component of extracts of the green alga *Caulerpa taxifolia,* exhibits antiproliferative activities as well as wound healing abilities with the latter resulting from rapid transformation to the highly reactive 1,4-dialdehyde, oxytoxin 2 (**4**) [4–6]. Oxytoxin 2 (**4**) is itself a natural product, produced by the mollusc *Oxynoe olivacea* from a diet-derived (*Caulerpa* algae) precursor and is predominantly present in the predator-deterring mucous secretion of the mollusc [7]. Two structurally-related masked dialdehydes, **5** (from *Caulerpa ashmeadii*) [8] and onchidal (**6**) [9–10] (from the defensive secretion of the mollusc *Onchidella binneyi*) also exhibit biological properties including feeding deterrence, antibacterial and anticholinesterase activities.

Chemical reactivity studies using polygodial (**1**), scalaradial (**2**) and caulerpenyne (**3**) have demonstrated evidence of pyrrole formation upon reaction with primary amines, with conclusions drawn attributing bioactivities such as antifeedant activity to this chemical reactivity [1,11–13]. In an effort to ascertain whether the mollusc metabolite onchidal is susceptible to nucleophilic attack in a similar manner, herein we report on the reactivity of onchidal and a library of simplified *n*-pentyl and cyclohexylmethyl model compounds towards thiol and amine nucleophiles as well as their reactivity towards a model protein target, lysozyme.

## Results and Discussion

**Scheme 1:**
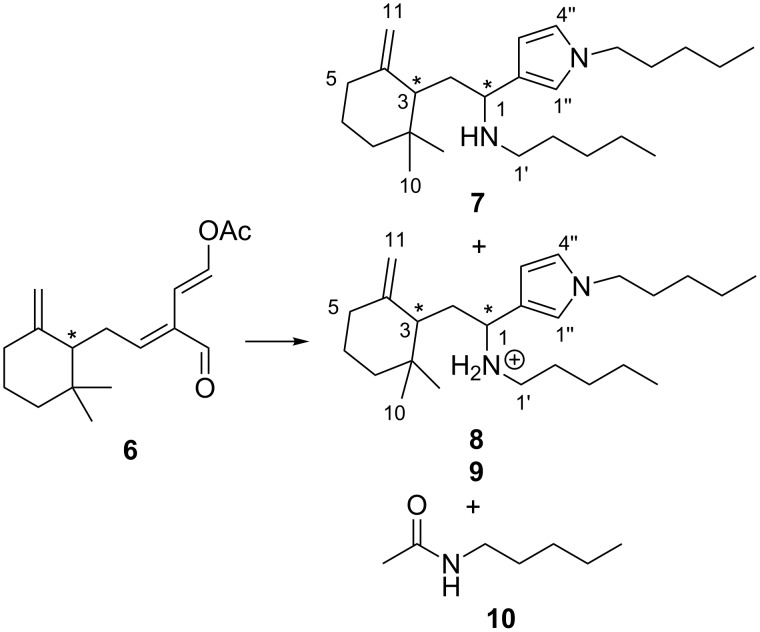
Products of the reaction of onchidal (**6**) with 1-pentylamine. Reagents and conditions: 1-pentylamine (excess), CDCl_3_, overnight.

Preliminary studies of the reactivity of onchidal (**6**) towards 1-pentanethiol or 1-pentylamine were undertaken in CDCl_3_ solvent in an NMR tube. Somewhat to our surprise, no reaction was observed with 1-pentanethiol, even with incubation in the presence of excess thiol for one week [14]. In contrast, incubation with excess 1-pentylamine rapidly afforded a mixture of products, as identified by changes in the ^1^H NMR spectrum. Signals attributable to *N*-alkyl-3-substituted pyrroles **7**–**9** and *N*-pentylacetamide **10** [δ_H_ 7.62 t, *J* = 4.7 Hz; 2.28 m] were observed. Purification by silica gel column chromatography, eluting with CH_2_Cl_2_, afforded pyrrole adduct **7** as the free base. Elution with CH_2_Cl_2_/MeOH afforded two fractions with the first comprised of a single diastereomer as a salt **8**, while a second fraction was obtained as a diastereomeric mixture (**8**:**9**, 3:1), again as salts (Scheme 1). Mass spectrometric data observed for **7** supported the formation of a diaminated pyrrole product, with a protonated molecular ion of *m/z* 373.3556 [M + H]^+^ corresponding to a formula of C_25_H_45_N_2_ (requires 373.3577). NMR data further supported such a structure, with pyrrole signals observed at [δ_H_ 6.57–6.55, m, H-1" and H-4"; 6.06, br s, H-3"; δ_C_ 120.6 (C-2" and C-4"); 118.5 (C-1"); 106.8 (C-3")] and pentylamine substitution at C-1 [δ_H_ 3.50–3.46, m; δ_C_ 54.1]. In the case of the more polar products **8** and **9**, (+)-ESIMS derived the same formula as for **7**, while differences observed in ^1^H NMR shifts for H-1/H-2/H-1' between **7** and **8** [δ**_8_**_–_**_7_**, Δδ +1.29–0.42] suggested **8**/**9** were purified as salts.

**Scheme 2:**
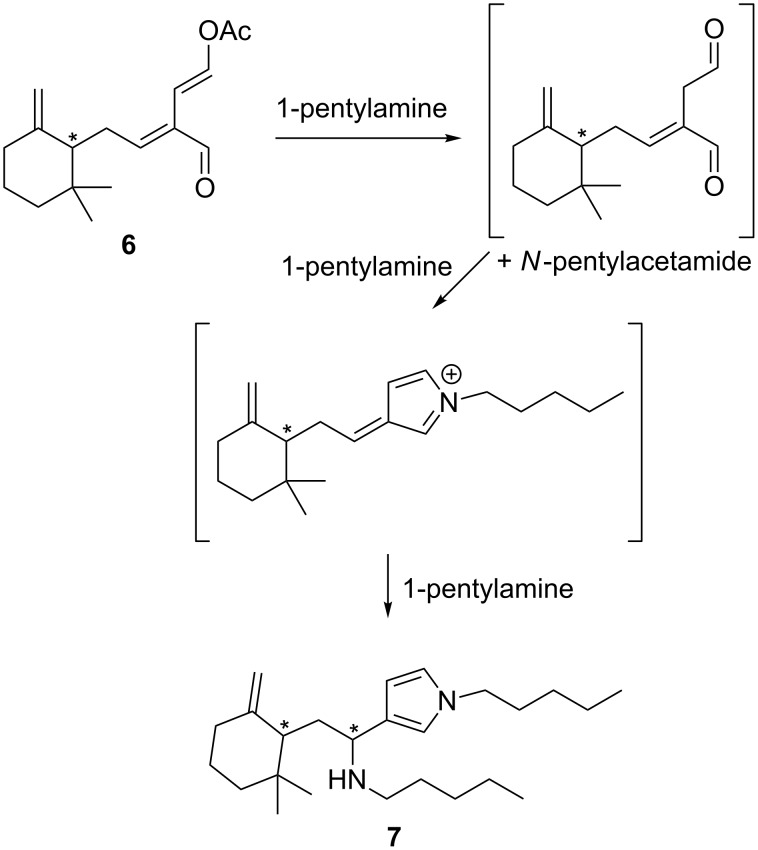
Proposed mechanism for formation of onchidal diaminated adducts.

A mechanism that leads to the formation of diaminated pyrrole adduct **7** starts with amine-induced formation of a 1,4-dialdehyde, which then undergoes Paal–Knorr pyrrole formation to give an azafulvinium intermediate (Scheme 2). This intermediate could then undergo trapping with an additional mole of amine nucleophile to give **7** as a mixture of diastereomers.

**Figure 2:**
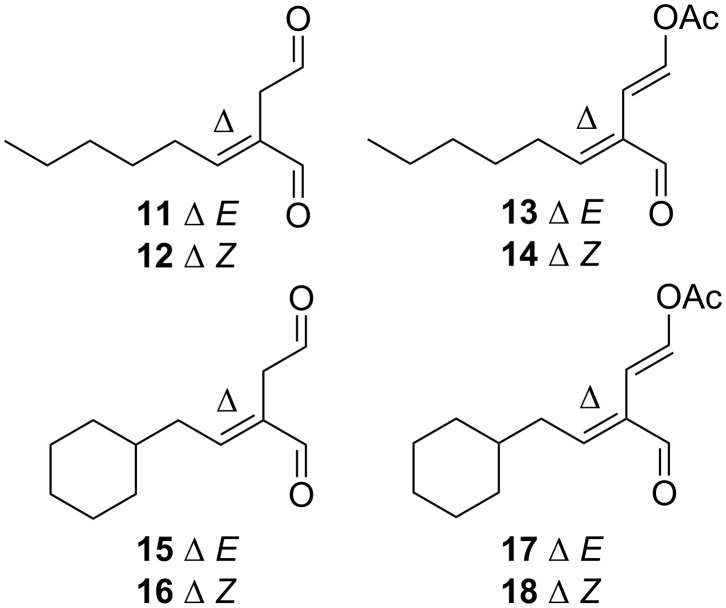
Target onchidal model compounds **11**–**18**.

In an effort to reduce the complexity of the NMR spectra observed for the diastereomeric onchidal–pyrrole adducts, a range of simpler achiral *n*-pentyl **11**–**14** and cyclohexylmethyl **15**–**18** side-chained model compounds, as either the dialdehyde or masked dialdehyde variants, were prepared (Figure 2).

**Scheme 3:**
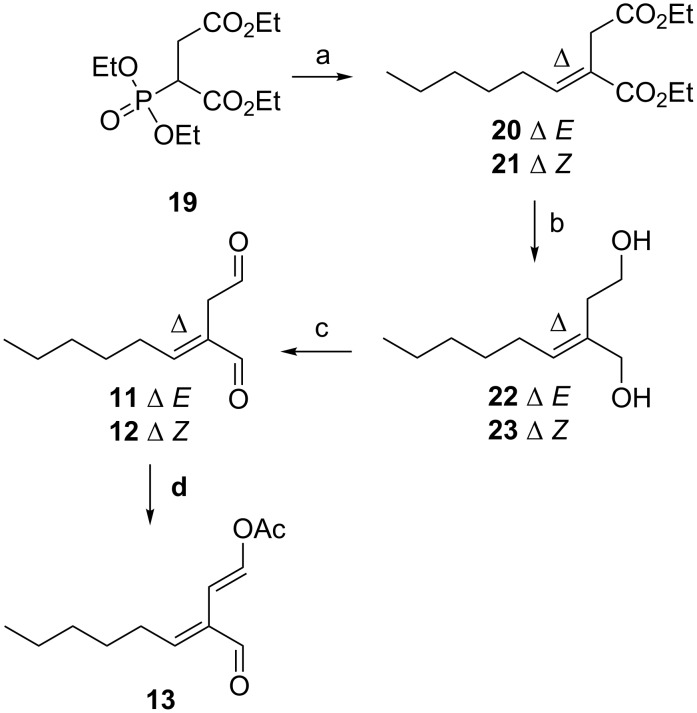
Synthesis of *n*-pentyl dialdehydes **11** and **12** and enol acetate **13**. Reagents and conditions: a) *n*-hexanal (0.8 equiv), LiOH·H_2_O (1.2 equiv), THF, 4 h, 60% (**20**), 10% (**21**); b) LiAlH_4_ (2.5 equiv), Et_2_O, 0 °C, 1 h, 63% (**22**), 67% (**23**); c) DMP (2.5 equiv), CH_2_Cl_2_, 4 h, 31% (**11**); d) Ac_2_O (2 equiv), pyridine (4 equiv), overnight, 17% (**13**).

Horner–Wadsworth–Emmons (H.W.E.) reaction of *n*-hexanal with phosphonoester **19** [15] afforded an *E*/*Z* mixture of olefinic diesters, purification of which by silica gel column chromatography afforded a fraction of the desired *E* diester **20** (60%), a second fraction comprised of a 5:1 *E*/*Z* mixture and a third fraction of *Z* diester **21** (10%, Scheme 3). The reduction of diesters **20** (*E*) and **21** (*Z*) with LiAlH_4_ afforded diols **22** and **23** in 63% and 67% yield, respectively. Subsequent oxidation of **22** with DMP afforded dialdehyde **11** in 31% yield. Correspondingly, the reaction of diol **23** with DMP afforded a mixture of dialdehyde **11** with dialdehyde **12** (1:1). Attempts at chromatographic separation of these two isomers resulted in degradation of **12**. Final conversion of **11** to enol acetate **13** was achieved by overnight reaction with pyridine and acetic anhydride. Purification by silica gel column chromatography afforded the desired *E,E* enol acetate **13** in 17% yield. A lack of purified dialdehyde **12** prevented any attempt at the preparation of enolacetate **14**.

**Scheme 4:**
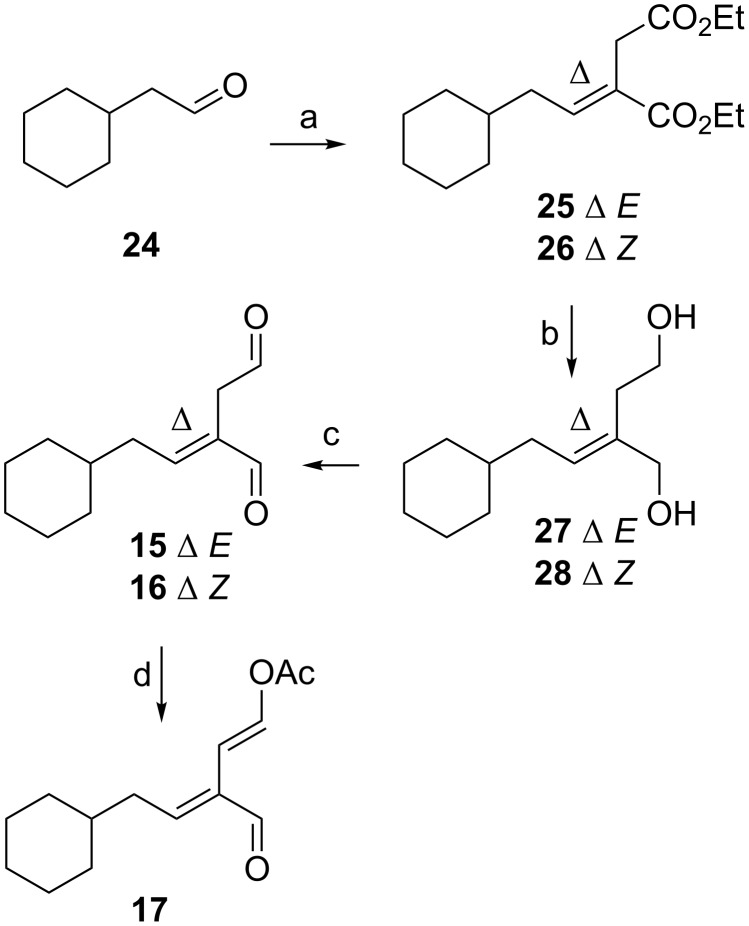
Synthesis of cyclohexylmethyl dialdehydes **15** and **16** and enol acetate **17**. Reagents and conditions: a) phosphonate **19** (1.3 equiv), LiOH·H_2_O (1.5 equiv), THF, 4 h, 15% (**25**), 1.5% (**26**); b) LiAlH_4_ (2.5 equiv), Et_2_O, 0 °C, 1 h, 61% (**27**), 71% (**28**); c) DMP (2.5 equiv), CH_2_Cl_2_, 4 h, 49% (**15**), 73% (**16**); d) Ac_2_O (2 equiv), pyridine (4 equiv), overnight, 43% (**17**).

Having developed a successful synthetic route to *n*-pentyl side-chain dialdehyde **11** and enol acetate **13**, the synthesis of analogues **15**–**18** with a side-chain more comparable to onchidal (**6**) were attempted. H.W.E reaction of 2-cyclohexylacetaldehyde (**24**) [16] with phosphonoester **19** afforded a fraction of the desired *E* diester **25** in 15% yield, a fraction of *Z* diester **26** in 1.5% yield and another fraction of a mixture of the two (5:1) (Scheme 4). The reaction of diesters **25** and **26** with LiAlH_4_ afforded the corresponding diols **27** and **28** in 61% and 71% yield, respectively, which upon oxidation (DMP) afforded dialdehydes **15** and **16** in 49% and 73% yield, respectively. The reaction of dialdehyde **15** with Ac_2_O and pyridine afforded enol acetate **17** in 43% yield after purification. Interestingly, the reaction of dialdehyde **16** with Ac_2_O/pyridine only afforded decomposition products, failing to give **18**.

**Figure 3:**
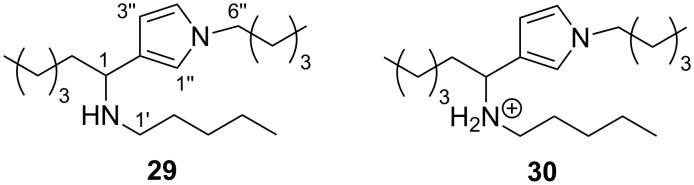
Pyrrole product **29** and salt **30** obtained from the reaction of dialdehyde **11** with *n*-pentylamine.

The electrophilic reactivity of model dialdehydes **11** and **15** and enol acetates **13** and **17** towards 1-pentanethiol and 1-pentylamine were then studied. As found for onchidal, no reaction (NMR tube) between **11**/**13**/**15**/**17** and 1-pentanethiol was detected, even after one week of incubation. In direct contrast, all four model compounds reacted rapidly with 1-pentylamine, forming pyrrole adducts. The reaction of dialdehyde **11** with 1-pentylamine afforded pyrrole adduct **29** almost instantaneously as determined by ^1^H NMR. Purification of the crude reaction product gave **29** as the free base (15% yield) and as the salt, **30** (also 15% yield, Figure 3). Spectroscopic and spectrometric analysis of **29** confirmed the formation of a diamine adduct, with detection of a protonated molecular ion in the (+)-ESI mass spectrum at *m*/*z* 307.3097 (C_20_H_39_N_2_ requires 307.3108) and NMR signals appropriate for a 3-substituted *N*-alkylpyrrole [δ_H_ 6.57 dd, *J* = 2.3, 2.3 Hz, H-4"; 6.54 br s, H-1"; 6.04 dd, *J* = 2.3, 2.3 Hz, H-3"; δ_C_ 120.8 (C-2"), 120.3 (C-4"), 118.4 (C-1"), 106.2 (C-3")].

**Scheme 5:**
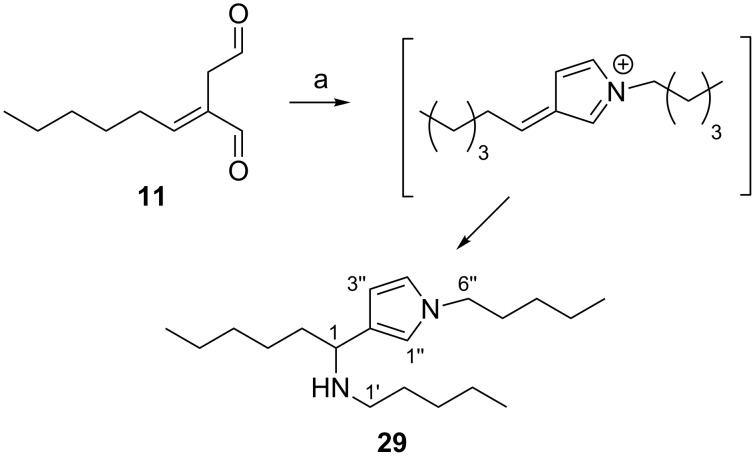
Reaction of dialdehyde **11** with excess 1-pentylamine to form **29**. Reagents and conditions: (a) 1-pentylamine (excess), CDCl_3_, overnight.

As proposed for the onchidal–diamine adduct, the formation of **29** is presumably a consequence of dialdehyde **11** undergoing Paal–Knorr pyrrole formation to form an azafulvenium intermediate which is subsequently quenched with another mole of amine nucleophile to form the observed product (Scheme 5).

**Figure 4:**
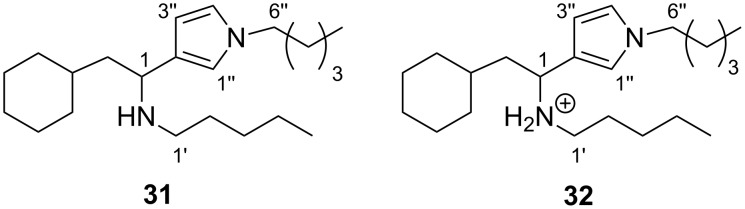
Pyrrole product **31** and salt **32** obtained from reaction of dialdehyde **15** with *n*-pentylamine.

Similar reactivity profiles were observed for each of cyclohexylmethyl dialdehyde **15**, and enol acetates **13** and **17**, with no reactivity towards 1-pentanethiol being detected, but with rapid reaction with 1-pentyamine to form pyrrole adducts. In the case of dialdehyde **15**, the reaction product was determined to be **31** (12% plus 18% as the salt, **32**, Figure 4), while enol esters **13** and **17** gave **29** and **31** (7% and 5% yields), respectively, upon reaction with the amine nucleophile.

We next investigated the reactivity of onchidal (**6**) and analogues **11**–**13** and **15**–**17** towards the lysine-rich model protein lysozyme. Previous studies have reported hen egg white lysozyme (HEWL) as a suitable target of electrophiles due to its commercial availability, a well-characterized amino acid sequence and the ability for routine (+)-ESIMS analysis to identify covalent adduct formation [17].

**Figure 5:**
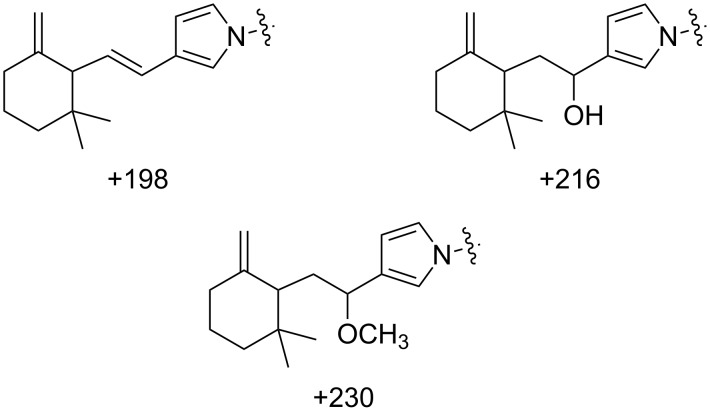
Lysine adducts arising from the reaction of onchidal (**6**) with lysozyme.

Reactivity studies were conducted with commercially available HEWL, in a solution of MeOH/H_2_O (+ 0.5% formic acid), and the reaction products were investigated by (+)-ESIMS. Preliminary reaction of onchidal (**6**) with lysozyme was conducted in a solvent mixture of MeOH/H_2_O (1:15) at 20 °C and examined regularly by (+)-ESIMS. No adducts were detected at 20 hours, but by day 3 (72 h), three new peaks representing mass additions of +198 mu, +216 mu, and +230 mu were detected (Figure 5 and Table 1). These adducts are likely the result of the reaction of lysine residues present in the enzyme [17]. The latter two adducts are proposed to be pyrrole adducts with incorporation of solvolytic H_2_O and methanol, respectively. The +198 mu adduct could have arisen via elimination of H_2_O or methanol from the corresponding adducts, or alternatively, from deprotonation of the anticipated lysozyme-onchidal azafulvenium intermediate. The adduct product distributions were calculated from the deconvoluted (+)-ESI mass spectrum, identifying a total lysozyme modification yield of 15% (Table 1). The presence of a large amount of unmodified lysozyme (85%), even after 72 h, was attributed to the slow reactivity of the enol acetate functionality of onchidal, as observed in the original model studies.

**Table 1:** Summary of lysozyme modifications by onchidal (**6**) and analogues **11**–**13** and **15**–**17**.^a^

No.	unmod (%)^b^	+1 (%)^c^			+2 (%)^c^		
	
		alkene^d^	OH^d^	OCH_3_^d^	alkene^d^	OH^d^	OCH_3_^d^

**6^a,e^**	85	5	4	6	0	0	0
**11^f^**	37	24	18	21	0	0	0
**12**	13	10	8	21	5	0	14
**13**	82	0	18	0	0	0	0
**15**	10	14	7	15	11	0	11
**16**	30	18	30	22	0	0	0
**17^e^**	93	2	3	2	0	0	0

^a^Standard reaction conditions: 50 µM substrate, 10 µM lysozyme, in MeOH/H_2_O at 20 °C for 20 hours (unless otherwise noted). Product distribution determined from deconvoluted (+)-HRESIMS data. ^b^Percentage of unmodified lysozyme. ^c^Percentage of mono-adduct (+1) and di-adduct (+2) products detected by (+)-ESIMS. ^d^Alkene-, hydroxy and methoxy group containing adducts detected. In the case of di-adducts, ions observed consistent for mixed nucleophilic quenching products, i.e., one hydroxy and one methoxy group are not reported in the Table. ^e^Incubation time of 3 days. ^f^Incubation time of 4 hours.

Next, the reactivity of dialdehydes **11**, **12**, **15** and **16** and enol acetates **13** and **17** with lysozyme were examined in a similar manner with mass spectrometry identifying varying degrees of modification. Of the dialdehydes, **11** was the most reactive leading to rapid formation of a white precipitate, speculated to be due to formation of insoluble higher order protein adducts. ESIMS analysis of the supernatant identified only a trace of unreacted lysozyme and detection of ions arising from extensive modification of the enzyme. To simplify the analysis of these adducts, the incubation time for **11** was shortened to 4 hours, with resultant ESIMS analysis identifying the presence of the three expected pyrrole adducts with mass additions of +132, +150 and +164 (Table 1). Interestingly, *Z*-dialdehyde **12**, formed the same adducts as **11** but at a much slower rate, requiring overnight incubation. In addition to the expected mono-adducts [+132, +150, +164], lysozyme di-adducts were also detected at +264 (2 × alkene), +282 (alkene and OH), +296 (alkene and OMe), +314 (OH and OMe), and +328 (2 × OMe).

Similar reactivity was observed for cyclohexylmethyl *E*-dialdehyde **15**, leading to the formation of a range of mono- (+158, +176, +190) and di-adducts (+316 [2 × alkene], +334 [alkene and OH], +348 [alkene and OMe], +366 [OH and OMe], +380 [2 × OMe]) (Table 1), while *Z*-dialdehyde **16** was comparatively less reactive, forming only mono-adducts. As expected, enol acetates **13** and **17** were only slowly reactive, giving 18% and 7% yield of adducts, respectively, with **17** requiring 72 hour incubation.

**Figure 6:**
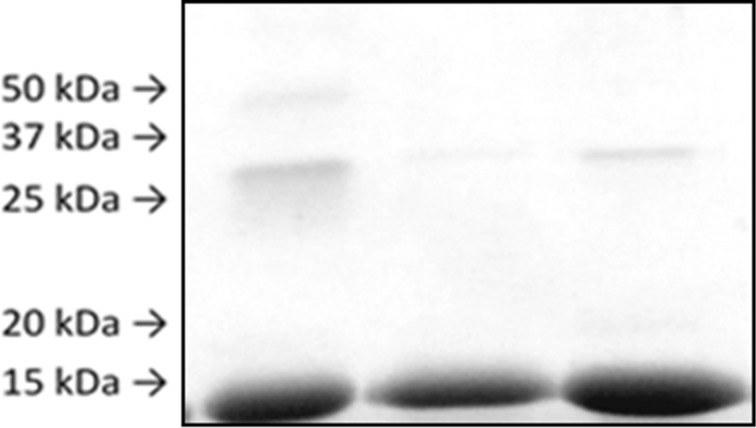
SDS-PAGE separation of lysozyme after modification with **11** (left), **13** (middle), **15** (right).

Sodium dodecyl sulfate polyacrylamide gel electrophoresis (SDS-PAGE) was used to look for the presence of protein crosslinking arising from the incubation of dialdehydes **11** and **15** and enol acetate **13** with lysozyme. Bands corresponding to dimers (28 kDa) were evident for both the *n*-pentyl and cyclohexylmethyl dialdehydes, with a faint band at 50 kDa also evident in the *n*-pentyl dialdehyde incubation reaction, indicating the presence of lysozyme trimers (Figure 6). No crosslinking was detected for enol acetate **13**, likely due to its low reactivity as determined from the *n*-pentylamine incubation studies.

## Conclusion

A chemical reactivity study of the opisthobranch mollusc metabolite onchidal (**6**) has identified that it can react with amines to form pyrrole products. The reaction was presumed to proceed via amine-mediated conversion of the enolester containing natural product to a 1,4-dialdehyde, which then undergoes Paal–Knorr pyrrole formation. Structurally simplified *n*-pentyl- and cyclohexylmethyl-dialdehydes were synthesized and found to undergo similar pyrrole forming reactions with pentylamine. These reactions were also apparent with the lysine-rich enzyme hen egg white lysozyme, with onchidal (**6**) and model compounds **11**–**13** and **15**–**17** affording pyrrole adducts of the enzyme that were detected by (+)-ESIMS. The more reactive dialdehydes were also found to lead to protein crosslinking with formation of lysozyme dimers and trimers. Taken together, these results support the hypothesis that onchidal (**6**) could be used in chemical defense in a similar manner to related sesquiterpenoid dialdehydes and enol esters.

## Supporting Information

File 1 Experimental procedures and characterization data of new compounds.

File 2 ^1^H and ^13^C NMR spectra of synthesized compounds.
