# Supplementary material for: Investigation of the electrophilic reactivity of the biologically active marine sesquiterpenoid onchidal and model compounds
Source: Beilstein J Org Chem. 2018 Aug 24;14:2229–35. doi: 10.3762/bjoc.14.197 (PMC6122400; doi:10.3762/bjoc.14.197)
Supplement: File 1 — Experimental procedures and characterization data of new compounds. [file Beilstein_J_Org_Chem-14-2229-s001.pdf]

Supporting Information File 1
for

Investigation of the electrophilic reactivity of the biologically active marine sesquiterpenoid onchidal and model compounds

Melissa M. Cadelis[1]* and Brent R. Copp[1]

Address: [1]School of Chemical Sciences, University of Auckland, Private Bag 92019, Auckland 1142, New Zealand

Email: Melissa M. Cadelis - m.cadelis@auckland.ac.nz

* Corresponding author

Experimental procedures and characterization data of new compounds

Contents

General experimental details S2
Extraction and isolation procedures S2
Experimental procedures and compound data S2
General procedure for protein modification S13
General procedure for SDS-PAGE S13
References S14

General experimental details

Infrared spectra were recorded on a Perkin-Elmer spectrometer. Mass spectra were acquired on a Bruker micrOTOF Q II spectrometer. ^{1}H and ^{13}C NMR spectra were recorded at 298 K on Bruker AC300, AVANCE 400 or 500 spectrometer using standard pulse sequences with TMS as an internal standard. NMR spectra were assigned using 2D NMR data. Silica gel column chromatography was carried out using Davisil silica gel (40–60 μm) or Merck silica gel (15–40 μm). Thin layer chromatography was conducted on Merck DC-plastikfolien Kieselgel 60 F254 plates. Phosphonate **19** and 2-cyclohexylethanal (**24**) were prepared by literature procedures [1,2].

Extraction and isolation procedures

Specimens of *Onchidella binneyi* were collected from Meola Reef, Western Springs, New Zealand. The molluscs were kept frozen until used. The frozen specimen (161 animals) were immersed in MeOH (2 × 100 mL) for 4 hours, filtered and concentrated under reduced pressure. The specimen were then immersed in CH_2Cl_2 (2 × 100 mL) for 4 hours, filtered and concentrated under reduced pressure. The combined extracts were purified by diol bonded silica gel column chromatography, eluting with *n*-hexane/Et_2O (99:1), to afford onchidal (**6**, 0.004 g) as a colourless oil. $[\alpha]_D^{23}$ +15.8 (c 0.43, $CHCl_3$) [lit.[3] +17.2 (c 1.01, $CHCl_3$)]. ^{1}H NMR data were in agreement with literature [3].

Experimental procedures and compound data

***N*-(2-(2,2-Dimethyl-6-methylenecyclohexyl)-1-(1-pentyl-1*H*-pyrrol-3-yl)ethyl)pentan-1-amine (7–9)**

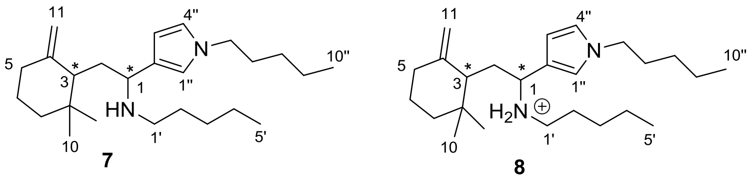

7 **8** **9**

To a solution of onchidal (**6**, 0.002 g, 0.007 mmol) in $CDCl_3$ (0.4 mL) was added 1-pentylamine (0.001 g, 0.014 mmol). The reaction was left overnight and solvent removed under reduced pressure. Purification by silica gel column chromatography, eluting with CH_2Cl_2, afforded **7** as an orange oil (0.0015 g, 56%) and elution with CH_2Cl_2/MeOH (9:1) afforded a mixture of **8** and **9** (3:1) as an orange oil (0.001 g, 37%).

7: R_f (CH_2Cl_2/MeOH, 9:1) 0.31; IR (ATR) ν_{max} 2978, 2874, 1446, 1384, 1152, 1110, 1076, 908, 730 cm^{-1}; ^{1}H NMR ($CDCl_3$, 500 MHz) δ 6.57–6.55 (2H, m, H-1'', H-4''), 6.06 (1H, br s, H-3''), 4.80 (1H, br s, H_2-11_A), 4.62 (1H, br s, H_2-11_B), 3.80 (2H, t, *J* = 7.2 Hz, H_2-6''), 3.50–3.46 (1H, m, H-1), 2.59–2.54

(1H, m, H_2-1'$_A$), 2.41–2.36 (1H, m, H_2-1'$_B$), 2.07–2.02 (3H, m, H-3, H_2-5), 1.78–1.72 (4H, m, H_2-2, H_2-7''), 1.54–1.48 (4H, m, H_2-6, H_2-2'), 1.32–1.22 (8H, m, H_2-3', H_2-4', H_2-8'', H_2-9''), 1.20–1.17 (2H, m, H_2-7), 0.94 (3H, s, H_3-9), 0.91–0.85 (9H, m, H_3-10, H_3-5', H_3-10''); ^{13}C NMR ($CDCl_3$, 125 MHz) δ 149.9 (C-4), 120.6 (C-2'', C-4''), 118.5 (C-1''), 109.3 (C-11), 106.8 (C-3''), 54.1 (C-1), 50.7 (C-3), 49.8 (C-6''), 47.1 (C-1'), 35.5 (C-8), 34.9 (C-7), 32.2 (C-5), 31.3 (C-7''), 29.9 (C-2), 29.7 (C-2'), 29.1 (C-8''), 28.3 (C-9), 26.93 (C-10/C-3'), 26.88 (C-10/C-3'), 23.8 (C-6), 22.7 (C-9''), 22.4 (C-4'), 14.14 (C-10''), 14.10 (C-5'); (+)-HRESIMS $[M+H]^+$ *m/z* 373.3556 (calcd for $C_{25}H_{45}N_2$, 373.3577).

8: R_f (CH_2Cl_2/MeOH, 9:1) 0.11; IR (ATR) v_{max} 2977, 2861, 1445, 1381, 1350, 1120, 1045, 955, 672 cm^{-1}; ^{1}H NMR ($CDCl_3$, 500 MHz) δ 6.79 (1H, dd, *J* = 1.6, 1.6 Hz, H-1''), 6.60 (1H, dd, *J* = 2.3, 2.3 Hz, H-4''), 6.23 (1H, dd, *J* = 2.3, 2.3 Hz, H-3''), 4.81 (1H, br s, H_2-11$_A$), 4.57 (1H, br s, H_2-11$_B$), 3.92 (1H, dd, *J* = 11.8, 2.6 Hz, H-1), 3.83 (2H, t, *J* = 7.1 Hz, H_2-6''), 2.75–2.70 (1H, m, H_2-1'$_A$), 2.66–2.61 (1H, m, H_2-1'$_B$), 2.49–2.45 (1H, m, H_2-2$_A$), 2.38–2.32 (1H, m, H_2-5$_A$), 2.26–2.21 (1H, m, H_2-2$_B$), 2.00–1.95 (1H, m, H_2-5$_B$), 1.82–1.79 (2H, m, H_2-2'), 1.77–1.71 (2H, m, H_2-7''), 1.60–1.56 (2H, m, H-3, H_2-6$_A$), 1.48–1.46 (1H, m, H_2-6$_B$), 1.34–1.25 (4H, m, H_2-8'', H_2-9''), 1.23–1.14 (6H, m, H_2-7, H_2-3', H_2-4'), 0.87 (3H, t, *J* = 7.1 Hz, H_3-10''), 0.83 (3H, t, *J* = 6.8 Hz, H_3-5'), 0.82 (3H, s, H_3-9), 0.80 (3H, s, H_3-10); ^{13}C NMR ($CDCl_3$, 125 MHz) δ 148.6 (C-4), 121.5 (C-4''), 121.1 (C-1''), 116.9 (C-2''), 110.3 (C-11), 108.4 (C-3''), 55.6 (C-1), 50.4 (C-3), 49.9 (C-6''), 44.9 (C-1'), 36.8 (C-8), 34.8 (C-7), 32.5 (C-5), 31.3 (C-7''), 30.3 (C-2), 29.3 (C-10, C-8''), 28.9 (C-3'), 28.1 (C-9), 25.6 (C-2'), 23.8 (C-6), 22.4 (C-9''), 22.2 (C-4'), 14.14 (C-10''), 14.12 (C-5'); (+)-HRESIMS $[M+Na]^+$ *m/z* 395.3382 (calcd for $C_{25}H_{44}N_2Na$, 395.3397).

9: R_f (CH_2Cl_2/MeOH, 9:1) 0.17; IR (ATR) v_{max} 2977, 2861, 1445, 1381, 1350, 1120, 1045, 955, 672 cm^{-1}; ^{1}H NMR ($CDCl_3$, 500 MHz) δ 6.84 (1H, dd, *J* = 1.8, 1.8 Hz, H-1''), 6.59 (1H, dd, *J* = 2.2, 2.2 Hz, H-4''), 6.22 (1H, dd, *J* = 2.2, 2.2 Hz, H-3''), 4.78 (1H, br s, H_2-11$_A$), 4.65 (1H, br s, H_2-11$_B$), 3.97–3.94 (1H, m, H-1), 3.82 (2H, t, *J* = 7.3 Hz, H_2-6''), 2.87–2.81 (1H, m, H_2-1'$_A$), 2.67–2.62 (1H, m, H_2-1'$_B$), 2.35–2.30 (1H, m, H_2-5$_A$), 2.24–2.19 (1H, m, H_2-2$_A$), 2.05–1.98 (1H, m, H_2-5$_B$), 1.99–1.93 (1H, m, H_2-2$_B$), 1.77–1.71 (4H, m, H_2-2', H_2-7''), 1.59–1.56 (1H, m, H-3), 1.49–1.42 (2H, m, H_2-6), 1.35–1.24 (4H, m, H_2-8'', H_2-9''), 1.23–1.13 (6H, m, H_2-7, H_2-3', H_2-4'), 0.95 (3H, s, H_3-9), 0.92 (3H, s, H_3-10), 0.89 (3H, t, *J* = 7.0 Hz, H_3-10''), 0.84 (3H, t, *J* = 6.9 Hz, H_3-5'); ^{13}C NMR ($CDCl_3$, 125 MHz) δ 149.8 (C-4), 121.4 (C-1''), 120.6 (C-4''), 117.2 (C-2''), 110.0 (C-11), 108.3 (C-3''), 55.6 (C-1), 50.2 (C-3), 50.0 (C-6''), 44.8 (C-1'), 36.4 (C-8), 35.2 (C-7), 32.3 (C-5), 31.2 (C-7''), 29.9 (C-2), 29.04 (C-8''), 28.96 (C-3'), 28.1 (C-9), 26.7 (C-10), 25.6 (C-2'), 23.6 (C-6), 22.4 (C-9''), 22.1 (C-4'), 14.1 (C-10''), 14.0 (C-5'); (+)-HRESIMS $[M+Na]^+$ *m/z* 395.3382 (calcd for $C_{25}H_{44}N_2Na$, 395.3397).

(*E*)-Diethyl 2-hexylidenesuccinate (20); (*Z*)-diethyl 2-hexylidenesuccinate (21)

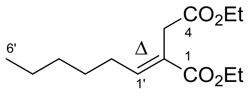

20 Δ *E*
21 Δ *Z*

Hexanal (0.035 g, 0.35 mmol) in THF (5 mL) was added to a solution of diethyl 2-(diethoxyphosphoryl)succinate (**19**, 0.143 g, 0.46 mmol) and lithium hydroxide monohydrate (0.022 g, 0.53 mmol) in THF (10 mL) at room temperature under nitrogen atmosphere. After stirring for 4 hours, the solvent was removed under reduced pressure. The residue was taken up in 1 N NaOH (10 mL) and washed with CH_2Cl_2 (2 × 10 mL). The organic layer was washed with 1 N NaOH (10 mL) and the combined aqueous layers were acidified to pH 2 with 1 M HCl. The aqueous layer was then extracted with EtOAc (2 × 10 mL) and the combined organic layers were washed with brine (10 mL), dried over anhydrous $MgSO_4$ and solvent removed under reduced pressure. Purification by silica gel column chromatography (CH_2Cl_2), afforded the *E* and *Z* isomers of the title compound (0.071 g, 78%). Further purification afforded the *E* isomer **20** in 60% yield and the *Z* isomer **21** in 10% and a third fraction of the *E/Z* mixture in a 5:1 ratio.

20: R_f (CH_2Cl_2) 0.61; IR (ATR) ν_{max} 3017, 2971, 2946, 1739, 1366, 1229, 1217, 1206 cm^{-1}; ^{1}H NMR ($CDCl_3$, 300 MHz) δ 6.96 (1H, t, *J* = 7.5 Hz, H-1'), 4.20 (2H, q, *J* = 7.2 Hz, H_2-1''), 4.14 (2H, q, *J* = 7.1 Hz, H_2-1'''), 3.34 (2H, s, H_2-3), 2.18 (2H, dt, *J* = 7.5, 7.5 Hz, H_2-2'), 1.50–1.42 (2H, m, H_2-3'), 1.33–1.29 (4H, m, H_2-4', H_2-5'), 1.28 (3H, t, *J* = 7.2 Hz, H_3-2''), 1.25 (3H, t, *J* = 7.1 Hz, H_3-2'''), 0.89 (3H, t, *J* = 6.5 Hz, H_3-6'); ^{13}C NMR ($CDCl_3$, 75 MHz) δ 171.1 (C-4), 167.2 (C-1), 145.8 (C-1'), 125.8 (C-2), 60.90 (C-1''/C-1'''), 60.86 (C-1''/C-1'''), 32.6 (C-3), 31.6 (C-4'), 29.0 (C-2'), 28.3 (C-3'), 22.6 (C-5'), 14.4 (C-6'/C-2'''), 14.3 (C-6'/C-2'''), 14.1 (C-2''); (+)-HRESIMS $[M+Na]^+$ *m/z* 279.1573 (calcd for $C_{14}H_{24}NaO_4$, 279.1567).

21: R_f (CH_2Cl_2) 0.67; IR (ATR) ν_{max} 3017, 2971, 2946, 1739, 1366, 1229, 1217, 1206 cm^{-1}; ^{1}H NMR ($CDCl_3$, 300 MHz) δ 6.05 (1H, t, *J* = 7.4 Hz, H-1'), 4.20 (2H, q, *J* = 7.2 Hz, H_2-1''), 4.13 (2H, q, *J* = 7.1 Hz, H_2-1'''), 3.25 (2H, d, *J* = 0.8 Hz, H_2-3), 2.56 (2H, dt, *J* = 7.4, 7.4 Hz, H_2-2'), 1.48–1.39 (2H, m, H_2-3'), 1.33–1.29 (4H, m, H_2-4', H_2-5'), 1.28 (3H, t, *J* = 7.2 Hz, H_3-2''), 1.25 (3H, t, *J* = 7.1 Hz, H_3-2'''), 0.91–0.87 (3H, m, H_3-6'); ^{13}C NMR ($CDCl_3$, 75 MHz) δ 171.7 (C-4), 166.8 (C-1), 147.7 (C-1'), 125.2 (C-2), 60.8 (C-1'''), 60.4 (C-1''), 40.5 (C-3), 31.6 (C-4'), 29.7 (C-2'), 29.0 (C-3'), 22.6 (C-5'), 14.3 (C-6', C-2'''), 14.1 (C-2''); (+)-HRESIMS $[M+Na]^+$ *m/z* 279.1557 (calcd for $C_{14}H_{24}NaO_4$, 279.1567).

(*E*)-2-Hexylidenebutane-1,4-diol (22)

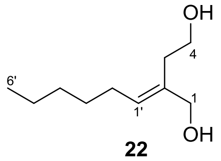

22

To a solution of $LiAlH_4$ (0.054 g, 1.40 mmol) in Et_2O (10 mL) at 0 °C was added (*E*)-diethyl 2-hexylidenesuccinate (**20**, 0.145 g, 0.56 mmol) under nitrogen atmosphere and stirred for 1 hour. The reaction was slowly diluted with EtOAc (10 mL) and quenched with 1 M HCl. The aqueous layer was extracted with EtOAc (2 × 5 mL) and the combined organic layers washed with brine (10 mL) and dried over anhydrous $MgSO_4$. Solvent removal under reduced pressure afforded the title compound as a colourless oil (0.061 g, 63%).

R_f (CH_2Cl_2) 0.33; IR (ATR) ν_{max} 3338, 2955, 2931, 1461, 1261, 1024, 762 cm^{-1}; ^1H NMR ($CDCl_3$, 500 MHz) δ 5.54 (1H, t, *J* = 7.4 Hz, H-1'), 4.02 (2H, s, H_2-1), 3.68 (2H, t, *J* = 5.9 Hz, H_2-4), 2.40 (2H, t, *J* = 5.9 Hz, H_2-3), 2.04 (2H, dt, *J* = 7.4, 7.4 Hz, H_2-2'), 1.39–1.27 (6H, m, H_2-3', H_2-4', H_2-5'), 0.89 (3H, t, *J* = 6.6 Hz, H_3-6'); ^{13}C NMR ($CDCl_3$, 125 MHz) δ 136.0 (C-2), 131.8 (C-1'), 68.6 (C-1), 61.9 (C-4), 32.5 (C-3), 31.7 (C-4'), 29.3 (C-3'), 27.8 (C-2'), 22.7 (C-5'), 14.1 (C-6'); (+)-HRESIMS $[M+Na]^+$ *m/z* 195.1348 (calcd for $C_{10}H_{20}NaO_2$, 195.1356).

(*Z*)-2-Hexylidenebutane-1,4-diol (23)

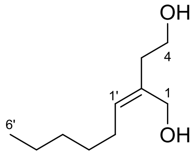

23

To a solution of $LiAlH_4$ (0.003 g, 0.070 mmol) in Et_2O (2 mL) at 0 °C was added (*Z*)-diethyl 2-hexylidenesuccinate (**21**, 0.009 g, 0.035 mmol) under nitrogen atmosphere and stirred for 1 hour. The reaction was slowly diluted with EtOAc (2 mL) and quenched with 1 M HCl. The aqueous layer was extracted with EtOAc (2 × 2 mL) and the combined organic layers washed with brine (5 mL) and dried over anhydrous $MgSO_4$. Solvent removal under reduced pressure afforded the title compound as a colourless oil (0.004 g, 67%).

R_f (CH_2Cl_2) 0.37; IR (ATR) ν_{max} 3377, 2975, 2931, 1650, 1267, 1049, 755 cm^{-1}; ^1H NMR ($CDCl_3$, 500 MHz) δ 5.42 (1H, t, *J* = 7.4 Hz, H-1'), 4.17 (2H, s, H_2-1), 3.75 (2H, t, *J* = 5.6 Hz, H_2-4), 2.37 (2H, t, *J* = 5.6 Hz, H_2-3), 2.09 (2H, dt, *J* = 7.4, 7.4 Hz, H_2-2'), 1.38–1.26 (6H, m, H_2-3', H_2-4', H_2-5'), 0.89 (3H, t, *J* = 6.8 Hz, H_3-6'); ^{13}C NMR ($CDCl_3$, 125 MHz) δ 135.9 (C-2) 132.3 (C-1'), 62.9 (C-4), 60.5 (C-1), 39.6

(C-3), 31.6 (C-4'), 29.7 (C-3'), 27.8 (C-2'), 22.7 (C-5'), 14.2 (C-6'); (+)-HRESIMS $[M+Na]^+$ *m/z* 195.1349 (calcd for $C_{10}H_{20}NaO_2$, 195.1356).

(*E*)-2-Hexylidenesuccinaldehyde (11)

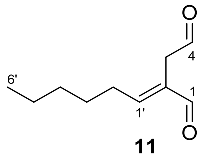

11

To a solution of (*E*)-2-hexylidenebutane-1,4-diol (0.070 g, 0.4 mmol) (**22**) in CH_2Cl_2 (5 mL) was added Dess–Martin periodinane (0.430 g, 1 mmol) under nitrogen atmosphere and stirred for 4 hours. The reaction was quenched with the slow addition of saturated aqueous $NaHCO_3$ and stirred for a further 15 minutes. The solution was filtered over a plug of celite and solvent removed under reduced pressure to afford the title compound as a colourless oil (0.021 g, 31%).

R_f (CH_2Cl_2) 0.61; IR (ATR) ν_{max} 2969, 2929, 2859, 1727, 1683, 1379, 953, 739 cm^{-1}; ^1H NMR ($CDCl_3$, 500 MHz) δ 9.61 (1H, t, *J* = 1.5 Hz, H-4), 9.46 (1H, s, H-1), 6.81 (1H, t, *J* = 7.5 Hz, H-1'), 3.41 (2H, br s, H_2-3), 2.31 (2H, dt, *J* = 7.5, 7.5 Hz, H_2-2'), 1.53–1.50 (2H, m, H_2-3'), 1.34–1.30 (4H, m, H_2-4', H_2-5'), 0.91–0.89 (3H, m, H_3-6'); ^{13}C NMR ($CDCl_3$, 125 MHz) δ 197.5 (C-4), 193.8 (C-1), 158.6 (C-1'), 135.3 (C-2), 39.2 (C-3), 31.6 (C-4'), 29.5 (C-3'), 28.1 (C-2'), 22.5 (C-5'), 14.0 (C-6'); (+)-HRESIMS $[M+H]^+$ *m/z* 169.1221 (calcd for $C_{10}H_{17}O_2$, 169.1223).

(*Z*)-2-Hexylidenesuccinaldehyde (12)

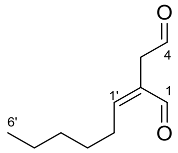

12

To a solution of (*Z*)-2-hexylidenebutane-1,4-diol (0.022 g, 0.13 mmol) (**23**) in CH_2Cl_2 (5 mL) was added Dess–Martin periodinane (0.135 g, 0.32 mmol) under nitrogen atmosphere and stirred for 4 hours. The reaction was quenched with the slow addition of saturated aqueous $NaHCO_3$, filtered over a plug of celite and solvent removed under reduced pressure to afford trace amounts of the title compound.

R_f (CH_2Cl_2) 0.68; ^1H NMR ($CDCl_3$, 300 MHz) δ 10.15 (1H, s, H-1), 9.65 (1H, t, *J* = 1.6 Hz, H-4), 6.62 (1H, t, *J* = 7.6 Hz, H-1'), 3.28 (2H, d, *J* = 0.8 Hz, H_2-3), 2.65 (2H, dt, *J* = 7.6, 7.6 Hz, H_2-2'), 1.57–1.48 (2H, m, H_2-3'), 1.35–1.28 (4H, m, H_2-4', H_2-5'), 0.91–0.89 (3H, m, H_3-6'); ^{13}C NMR ($CDCl_3$, 75 MHz) δ

198.7 (C-4), 189.0 (C-1), 153.9 (C-1'), 134.5 (C-2), 45.2 (C-3), 31.4 (C-4'), 29.2 (C-3'), 27.2 (C-2'), 22.5 (C-5'), 14.0 (C-6'); (+)-HRESIMS $[M+H]^+$ *m/z* 191.1046 (calcd for $C_{10}H_{16}NaO_2$, 191.1043).

(1*E*,3*E*)-3-Formylnona-1,3-dien-1-yl acetate (13)

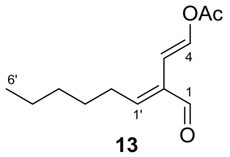

13

To (*E*)-2-hexylidenesuccinaldehyde (**11**, 0.019 g, 0.11 mmol) was added pyridine (0.036 g, 0.45 mmol) and acetic anhydride (0.023 g, 0.22 mmol) and stirred overnight under nitrogen atmosphere. The reaction mixture was washed with 1 M HCl (2 mL) and extracted with CH_2Cl_2 (2 × 5 mL). The combined organic layers were dried over anhydrous $MgSO_4$ and solvent removed under reduced pressure. Purification by silica gel column chromatography (*n*-hexane/EtOAc, 9:1) afforded the title compound as a colourless oil (0.004 g, 17%).

R_f (CH_2Cl_2) 0.64; IR (ATR) ν_{max} 2958, 2933, 1755, 1714, 1365, 1224, 1003 cm^{-1}; ^1H NMR ($CDCl_3$, 500 MHz) δ 9.44 (1H, d, *J* = 1.8 Hz, H-1), 8.23 (1H, d, *J* = 12.6 Hz, H-4), 6.48 (1H, t, *J* = 7.5 Hz, H-1'), 6.08 (1H, dd, *J* = 12.6, 7.5 Hz, H-3), 2.42 (2H, dt, *J* = 7.5, 7.5 Hz, H_2-2'), 2.17 (3H, s, H_3-2''), 1.56–1.53 (2H, m, H_2-3'), 1.36–1.28 (4H, m, H_2-4', H_2-5'), 0.93–0.89 (3H, m, H_3-6'); ^{13}C NMR ($CDCl_3$, 125 MHz) δ 193.6 (C-1), 167.8 (C-1''), 156.5 (C-1'), 141.3 (C-4), 135.7 (C-2), 105.2 (C-3), 31.6 (C-4'), 29.3 (C-2'), 28.4 (C-3'), 22.6 (C-5'), 20.8 (C-2''), 14.1 (C-6'); (+)-HRESIMS $[M+Na]^+$ *m/z* 233.1140 (calcd for $C_{12}H_{18}NaO_3$, 233.1148).

(*E*)-Diethyl 2-(2-cyclohexylethylidene)succinate (25); (*Z*)-diethyl 2-(2-cyclohexylethylidene)succinate (26)

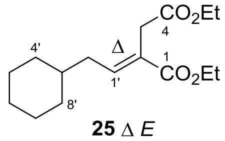

25 Δ *E*
26 Δ *Z*

2-Cyclohexylethanal (**24**, 0.60 g, 4.7 mmol) in THF (2 mL) was added to a solution of phosphonate **19** (1.92 g, 6.2 mmol) and lithium hydroxide monohydrate (0.30 g, 7.1 mmol) in THF (20 mL) at room temperature under nitrogen atmosphere. After stirring for 4 hours, solvent was removed under reduced pressure. The residue was taken up in 1 N NaOH (10 mL) and washed with CH_2Cl_2 (2 × 10 mL). The organic layer was washed with 1 N NaOH (10 mL) and the combined aqueous layers were acidified to pH 2 with 1 M HCl. The aqueous layer was then extracted with EtOAc (2 × 10 mL) and the

combined organic layers were washed with brine (10 mL), dried over anhydrous $MgSO_4$ and solvent removed under reduced pressure. Purification by silica gel column chromatography (CH_2Cl_2), afforded the *E* and *Z* isomers of the title compound (0.410 g, 31%). A fraction of the *E* isomer **25** was isolated in 15% yield, a fraction of the *Z* isomer **26** in 1.5% yield and a third fraction of the *E*/*Z* mixture.

25: R_f (CH_2Cl_2) 0.57; IR (ATR) ν_{max} 2970, 1742, 1366, 1217 cm^{-1}; ^1H NMR ($CDCl_3$, 500 MHz) δ 6.98 (1H, t, *J* = 7.5 Hz, H-1'), 4.20 (2H, q, *J* = 7.1 Hz, H_2-1''), 4.13 (2H, q, *J* = 7.1 Hz, H_2-1'''), 3.33 (2H, s, H_2-3), 2.08 (2H, dd, *J* = 7.5, 7.5 Hz, H_2-2'), 1.74–1.63 (6H, m, H_2-4', H_2-5', H_2-7'), 1.48–1.41 (1H, m, H-3'), 1.28 (3H, t, *J* = 7.1 Hz, H_3-2''), 1.24 (3H, t, *J* = 7.1 Hz, H_3-2'''), 1.22–1.12 (2H, m, H_2-6'), 0.98–0.90 (2H, m, H_2-8'); ^{13}C NMR ($CDCl_3$, 125 MHz) δ 171.0 (C-4), 167.1 C-1), 144.5 (C-1'), 126.3 (C-2), 60.78 (C-1''/C-1'''), 60.76 (C-1''/C-1'''), 37.8 (C-3'), 36.7 (C-2'), 33.3 (C-4', C-8'), 32.6 (C-3), 26.4 (C-5'/C-6'/C-7'), 26.3 (C-5'/C-6'/C-7'), 14.3 (C-2''/C-2'''), 14.2 (C-2''/C-2'''); (+)-HRESIMS $[M+Na]^+$ *m/z* 305.1721 (calcd for $C_{16}H_{26}NaO_4$, 305.1723).

26: R_f (CH_2Cl_2) 0.34; IR (ATR) ν_{max} 2970, 1742, 1366, 1217 cm^{-1}; ^1H NMR ($CDCl_3$, 500 MHz) δ 6.08 (1H, t, *J* = 7.3 Hz, H-1'), 4.19 (2H, q, *J* = 7.2 Hz, H_2-1''), 4.13 (2H, q, *J* = 7.2 Hz, H_2-1'''), 3.25 (2H, s, H_2-3), 2.47 (2H, dd, *J* = 7.3, 7.3 Hz, H_2-2'), 1.73–1.60 (6H, m, H_2-4', H_2-5', H_2-7'), 1.42–1.36 (1H, m, H-3'), 1.28 (3H, t, *J* = 7.2 Hz, H_3-2''), 1.24 (3H, t, *J* = 7.2 Hz, H_3-2'''), 1.22–1.13 (2H, m, H_2-6'), 1.00–0.93 (2H, m, H_2-8'); ^{13}C NMR ($CDCl_3$, 125 MHz) δ 171.7 (C-4), 166.8 C-1), 146.5 (C-1'), 125.7 (C-2), 60.8 (C-1'''), 60.4 (C-1''), 40.6 (C-3), 38.4 (C-3'), 37.2 (C-2'), 33.3 (C-4', C-8'), 26.6 (C-5'/C-6'/C-7'), 26.4 (C-5'/C-6'/C-7'), 14.3 (C-2'', C-2'''); (+)-HRESIMS $[M+Na]^+$ *m/z* 305.1712 (calcd for $C_{16}H_{26}NaO_4$, 305.1723).

(*E*)-2-(2-Cyclohexylethylidene)butane-1,4-diol (27)

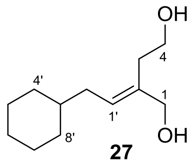

27

To a solution of $LiAlH_4$ (0.021 g, 0.56 mmol) in anhydrous diethyl ether (2 mL), (*E*)-diethyl 2-(2-cyclohexylethylidene)succinate (**25**, 0.063 g, 0.22 mmol) in Et_2O (2 mL) was added dropwise at 0 °C under nitrogen atmosphere. The mixture was left to warm to room temperature for an hour and quenched with the dropwise addition of 1 M HCl. The aqueous layer was extracted with CH_2Cl_2 (2 × 5 mL) and the combined organic layers were washed with brine (5 mL), dried over anhydrous $MgSO_4$ and solvent removed under reduced pressure to afford the title compound (0.027 g, 61%).

R_f (CH_2Cl_2) 0.22; IR (ATR) ν_{max} 3289, 2923, 2851, 1448, 1040, 1000, 871 cm^{-1}; ^1H NMR ($CDCl_3$, 500 MHz) δ 5.59 (1H, t, *J* = 7.2 Hz, H-1'), 4.06 (2H, s, H_2-1), 3.71 (2H, t, *J* = 6.0 Hz, H_2-4), 2.42 (2H, t, *J* = 6.0 Hz, H_2-3), 1.95 (2H, dd, *J* = 7.2, 7.2 Hz, H_2-2'), 1.72–1.63 (6H, m, H_2-4', H_2-5', H_2-7'), 1.33–1.25 (1H, m, H-3'), 1.23–1.12 (2H, m, H_2-6'), 0.94–0.86 (2H, m, H_2-8'); ^{13}C NMR ($CDCl_3$, 125 MHz) δ 136.6

(C-2), 130.5 (C-1'), 68.8 (C-1), 62.0 (C-4), 38.4 (C-3'), 35.6 (C-2'), 33.4 (C-4', C-8'), 32.6 (C-3), 26.6 (C-5'/C-6'/C-7'), 26.5 (C-5'/C-6'/C-7'); (+)-HRESIMS $[M+Na]^+$ *m/z* 221.1519 (calcd for $C_{12}H_{22}NaO_2$, 221.1512).

(*Z*)-2-(2-Cyclohexylethylidene)butane-1,4-diol (28)

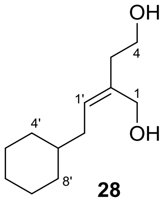

28

To a solution of $LiAlH_4$ (0.005 g, 0.12 mmol) in anhydrous diethyl ether (1 mL), (*Z*)-diethyl 2-(2-cyclohexylethylidene)succinate (**26**, 0.014 g, 0.05 mmol) in Et_2O (1 mL) was added dropwise at 0 °C under nitrogen atmosphere. The mixture was left to warm to room temperature for an hour and quenched with the dropwise addition of 1 M HCl. The aqueous layer was extracted with CH_2Cl_2 (2 × 5 mL) and the combined organic layers were washed with brine (5 mL), dried over anhydrous $MgSO_4$ and solvent removed under reduced pressure to afford the title compound (0.007 g, 71%).

R_f (CH_2Cl_2) 0.28; IR (ATR) ν_{max} 3308, 2920, 2850, 1448, 1043, 1013 cm^{-1}; ^1H NMR ($CDCl_3$, 500 MHz) δ 5.43 (1H, t, *J* = 7.4 Hz, H-1'), 4.15 (2H, s, H_2-1), 3.74 (2H, t, *J* = 5.6 Hz, H_2-4), 2.38 (2H, t, *J* = 5.6 Hz, H_2-3), 1.99 (2H, dd, *J* = 7.4, 7.4 Hz, H_2-2'), 1.70–1.63 (6H, m, H_2-4', H_2-5', H_2-7'), 1.30–1.22 (1H, m, H-3'), 1.21–1.12 (2H, m, H_2-6'), 0.94–0.89 (2H, m, H_2-8'); ^{13}C NMR ($CDCl_3$, 125 MHz) δ 136.6 (C-2), 130.7 (C-1'), 62.9 (C-4), 60.5 (C-1), 39.7 (C-3), 38.4 (C-3'), 35.6 (C-2'), 33.3 (C-4', C-8'), 26.6 (C-5'/C-6'/C-7'), 26.5 (C-5'/C-6'/C-7'); (+)-HRESIMS $[M+Na]^+$ *m/z* 221.1517 (calcd for $C_{12}H_{22}NaO_2$, 221.1512).

(*E*)-2-(2-Cyclohexylethylidene)succinaldehyde (15)

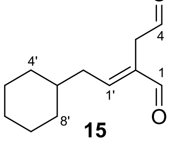

15

To a solution of (*E*)-2-(2-cyclohexylethylidene)butane-1,4-diol (**27**) (0.021 g, 0.11 mmol) in CH_2Cl_2 (2 mL) was added Dess–Martin periodinane (0.112 g, 0.26 mmol) under nitrogen atmosphere and stirred for 4 hours. The reaction was quenched with the slow addition of saturated aqueous $NaHCO_3$ and stirred for a further 15 minutes. The solution was filtered over a plug of celite and solvent removed under reduced pressure to afford the title compound as a colourless oil (0.010 g, 49%).

R_f (CH_2Cl_2) 0.41; IR (ATR) ν_{max} 2920, 2850, 1722, 1687, 1448 cm^{-1}; ^1H NMR ($CDCl_3$, 400 MHz) δ 9.60 (1H, t, *J* = 1.5 Hz, H-4), 9.47 (1H, s, H-1), 6.84 (1H, t, *J* = 7.4 Hz, H-1'), 3.39 (2H, br s, H_2-3), 2.22 (2H, dd, *J* = 7.4, 7.4 Hz, H_2-2'), 1.73–1.65 (6H, m, H_2-4', H_2-5', H_2-7'), 1.55–1.48 (1H, m, H-3'), 1.30–1.14 (2H, m, H_2-6'), 1.01–0.96 (2H, m, H_2-8'); ^{13}C NMR ($CDCl_3$, 100 MHz) δ 197.6 (C-4), 193.8 (C-1), 157.6 (C-1'), 136.0 (C-2), 39.4 (C-3), 37.8 (C-3'), 37.3 (C-2'), 33.4 (C-4', C-8'), 26.31 (C-5'/C-6'/C-7'), 26.27 (C-5'/C-6'/C-7'); (+)-HRESIMS $[M+Na]^+$ *m/z* 217.1194 (calcd for $C_{12}H_{18}NaO_2$, 217.1199).

(*Z*)-2-(2-Cyclohexylethylidene)succinaldehyde (16)

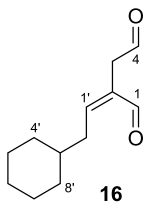

16

To a solution of (*Z*)-2-(2-cyclohexylethylidene)butane-1,4-diol (**28**, 0.007 g, 0.035 mmol) in CH_2Cl_2 (2 mL) was added Dess–Martin periodinane (0.037 g, 0.088 mmol) under nitrogen atmosphere and stirred for 4 hours. The reaction was quenched with the slow addition of saturated aqueous $NaHCO_3$ and stirred for a further 15 minutes. The solution was filtered over a plug of celite and solvent removed under reduced pressure to afford the title compound as a colourless oil (0.005 g, 73%).

R_f (CH_2Cl_2) 0.50; IR (ATR) ν_{max} 2923, 2853, 1732, 1675, 1366, 1230 cm^{-1}; ^1H NMR ($CDCl_3$, 400 MHz) δ 10.12 (1H, s, H-1), 9.65 (1H, t, *J* = 1.3 Hz, H-4), 6.63 (1H, t, *J* = 7.8 Hz, H-1'), 3.29 (2H, d, *J* = 1.3 Hz, H_2-3), 2.54 (2H, dd, *J* = 7.8, 7.8 Hz, H_2-2'), 1.77–1.65 (6H, m, H_2-4', H_2-5', H_2-7'), 1.52–1.44 (1H, m, H-3'), 1.22–1.14 (2H, m, H_2-6'), 1.04–0.96 (2H, m, H_2-8'); ^{13}C NMR ($CDCl_3$, 100 MHz) δ 198.7 (C-4), 189.9 (C-1), 152.7 (C-1'), 133.4 (C-2), 45.3 (C-3), 38.2 (C-3'), 34.8 (C-2'), 33.2 (C-4', C-8'), 26.5 (C-5'/C-6'/C-7'), 26.4 (C-5'/C-6'/C-7'), 26.3 (C-5'/C-6'/C-7'); (+)-HRESIMS $[M+Na]^+$ *m/z* 217.1197 (calcd for $C_{12}H_{18}NaO_2$, 217.1199).

(1*E*,3*E*)-5-Cyclohexyl-3-formylpenta-1,3-dien-1-yl acetate (17)

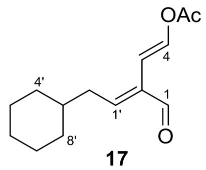

17

To (*E*)-2-(2-cyclohexylethylidene)succinaldehyde (**15**, 0.006 g, 0.03 mmol) was added pyridine (0.010 g, 0.12 mmol) and acetic anhydride (0.006 g, 0.06 mmol) and stirred overnight under nitrogen atmosphere. The reaction mixture was washed with 1 M HCl (1 mL) and extracted with CH_2Cl_2 (2 × 2

mL). The combined organic layers were dried over anhydrous $MgSO_4$ and solvent removed under reduced pressure. Purification by silica gel column chromatography (*n*-hexane/EtOAc, 9:1) afforded the title compound as a colourless oil (0.003 g, 43%).

R_f (CH_2Cl_2) 0.61; IR (ATR) ν_{max} 2972, 2924, 2850, 1739, 1448, 1381, 1218, 118, 1048 cm^{-1}; ^{1}H NMR ($CDCl_3$, 400 MHz) δ 9.45 (1H, d, *J* = 2.0 Hz, H-1), 8.25 (1H, d, *J* = 12.6 Hz, H-4), 6.52 (1H, t, *J* = 7.5 Hz, H-1'), 6.07 (1H, ddd, *J* = 12.6, 2.0, 0.8 Hz, H-3), 2.32 (2H, dd, *J* = 7.5, 7.5 Hz, H_2-2'), 2.17 (3H, s, H_3-2''), 1.75–1.69 (6H, m, H_2-4', H_2-5', H_2-7'), 1.54–1.50 (1H, m, H-3'), 1.22–1.15 (2H, m, H_2-6'), 1.04–0.95 (2H, m, H_2-8'); ^{13}C NMR ($CDCl_3$, 100 MHz) δ 193.6 (C-1), 167.8 (C-1''), 155.5 (C-1'), 141.3 (C-4), 136.2 (C-2), 105.3 (C-3), 38.1 (C-3'), 37.0 (C-2'), 33.4 (C-4', C-8'), 26.4 (C-5'/C-6'/C-7'), 26.3 (C-5'/C-6'/C-7'), 20.9 (C-2''); (+)-HRESIMS $[M+Na]^+$ *m/z* 259.1308 (calcd for $C_{14}H_{20}NaO_3$, 259.1305).

***N*-Pentyl-1-(1-pentyl-1*H*-pyrrol-3-yl)hexan-1-amine (29 and 30)**

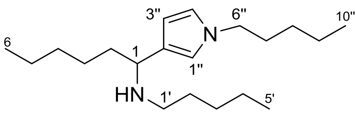

29

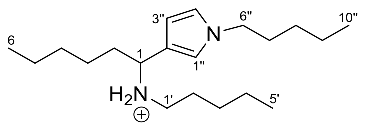

30

To a solution of (*E*)-2-hexylidenesuccinaldehyde (**11**, 0.014 g, 0.08 mmol) in $CHCl_3$ (2 mL) was added 1-pentylamine (0.007 g, 0.08 mmol) dropwise. The reaction was stirred for 1 hour and solvent removed under reduced pressure. Purification by silica gel column chromatography, eluting with CH_2Cl_2, afforded **29** as an orange oil (0.002 g, 15%) and elution with CH_2Cl_2/MeOH (9:1) afforded **30** as an orange oil (0.002 g, 15%).

29: R_f (CH_2Cl_2/MeOH, 9:1) 0.60; IR (ATR) ν_{max} 3274, 2956, 2930, 2859, 1552, 1460, 1380, 1166, 729 cm^{-1}; ^{1}H NMR ($CDCl_3$, 400 MHz) δ 6.57 (1H, dd, *J* = 2.3, 2.3 Hz, H-4''), 6.54 (1H, br s, H-1''), 6.04 (1H, dd, *J* = 2.3, 2.3 Hz, H-3''), 3.80 (2H, t, *J* = 7.0 Hz, H_2-6''), 3.58–3.54 (1H, m, H-1), 2.58–2.52 (2H, m, H_2-1'), 1.77–1.70 (2H, m, H_2-7''), 1.69–1.61 (2H, m, H_2-2), 1.56–1.44 (2H, m, H_2-2'), 1.34–1.21 (14H, m, H_2-3, H_2-4, H_2-5, H_2-3', H_2-4', H_2-8'', H_2-9''), 0.90–0.84 (9H, m, H_3-6, H_3-5', H_3-10''); ^{13}C NMR ($CDCl_3$, 125 MHz) δ 120.8 (C-2''), 120.3 (C-4''), 118.4 (C-1''), 106.2 (C-3''), 56.6 (C-1), 49.7 (C-6''), 47.6 (C-1'), 37.3 (C-2), 32.0 (C-3), 31.3 (C-7''), 29.79 (C-4/C-2'), 29.76 (C-4/C-2'), 29.0 (C-8''), 26.4 (C-3'), 22.74 (C-5/C-4'), 22.72 (C-5/C-4'), 22.4 (C-9''), 14.1 (C-6, C-5', C-10''); (+)-HRESIMS $[M+H]^+$ *m/z* 307.3097 (calcd for $C_{20}H_{39}N_2$, 307.3108).

30: R_f (CH_2Cl_2/MeOH, 9:1) 0.55; IR (ATR) ν_{max} 3345, 2957, 2927, 2859, 1555, 1466, 1379, 1167, 955 cm^{-1}; ^{1}H NMR ($CDCl_3$, 500 MHz) δ 6.78 (1H, br s, H-1''), 6.62 (1H, br s, H-4''), 6.22 (1H, br s, H-3''), 3.94 (1H, dd, *J* = 10.8, 3.4 Hz, H-1), 3.83 (2H, t, *J* = 7.0 Hz, H_2-6''), 2.76–2.59 (2H, m, H_2-1'), 2.18–2.13 (1H, m, H_2-2_A), 2.05–2.01 (1H, m, H_2-2_B), 1.77–1.72 (4H, m, H_2-2', H_2-7''), 1.32–1.20 (14H, m, H_2-3, H_2-4, H_2-5, H_2-3', H_2-4', H_2-8'', H_2-9''), 0.91–0.81 (9H, m, H_3-6, H_3-5', H_3-10''); ^{13}C NMR ($CDCl_3$, 125 MHz) δ 121.8 (C-4''), 120.7 (C-1''), 117.2 (C-2''), 107.8 (C-3''), 57.2 (C-1), 50.0 (C-6''), 45.1 (C-1'),

33.8 (C-2), 31.4 (C-4/C-7''), 31.2 (C-4/C-7''), 29.1 (C-8''), 28.9 (C-3'), 26.1 (C-3/C-2'), 25.8 (C-3/C-2'), 22.6 (C-5), 22.3 (C-4'/C-9''), 22.2 (C-4'/C-9''), 14.09 (C-6/C-5'/C-10''), 14.07 (C-6/C-5'/C-10''), 14.0 (C-6/C-5'/C-10''); (+)-HRESIMS $[M+H]^+$ *m/z* 307.3099 (calcd for $C_{20}H_{39}N_2$, 307.3108).

***N*-(2-Cyclohexyl-1-(1-pentyl-1*H*-pyrrol-3-yl)ethyl)pentan-1-amine (31 and 32)**

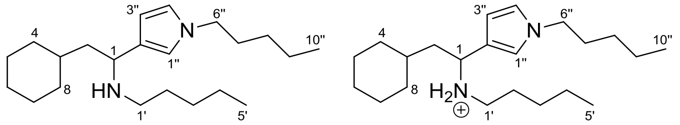

31 **32**

To a solution of (*E*)-2-(2-cyclohexylethylidene)succinaldehyde (**15**, 0.010 g, 0.05 mmol) in $CHCl_3$ (2 mL) was added 1-pentylamine (0.009 g, 0.10 mmol) dropwise. The reaction was stirred for 1 hour and solvent removed under reduced pressure. Purification by silica gel column chromatography, eluting with CH_2Cl_2, afforded **31** as an orange oil (0.002 g, 12%) and elution with CH_2Cl_2/MeOH (9:1) afforded **32** as an orange oil (0.003 g, 18%).

31: R_f (CH_2Cl_2/MeOH, 9:1) 0.60; IR (ATR) v_{max} 3415, 2961, 2920, 2853, 1501, 1448, 1380, 1160, 1130, 954 cm^{-1}; ^1H NMR ($CDCl_3$, 500 MHz) δ 6.56 (1H, dd, *J* = 2.3, 2.3 Hz, H-4''), 6.53 (1H, br s, H-1''), 6.03 (1H, dd, *J* = 2.3, 2.3 Hz, H-3''), 3.80 (2H, t, *J* = 7.1 Hz, H_2-6''), 3.65 (1H, t, *J* = 7.0 Hz, H-1), 2.57–2.46 (2H, m, H_2-1'), 1.76–1.71 (2H, m, H_2-7''), 1.65–1.60 (7H, m, H_2-2_A, H_2-4, H_2-5, H_2-7), 1.56–1.43 (3H, m, H_2-2_B, H_2-2'), 1.34–1.21 (9H, m, H-3, H_2-3', H_2-4', H_2-8'', H_2-9''), 1.16–1.13 (2H, m, H_2-6), 0.95–0.90 (2H, m, H_2-8), 0.88 (3H, t, *J* = 7.2 Hz, H_3-10''), 0.86 (3H, t, *J* = 7.2 Hz, H_3-5'); ^{13}C NMR ($CDCl_3$, 125 MHz) δ 120.6 (C-2'', C-4''), 118.7 (C-1''), 106.4 (C-3''), 53.8 (C-1), 49.7 (C-6''), 47.2 (C-1'), 45.0 (C-2), 34.7 (C-3), 34.0 (C-8), 33.2 (C-4), 31.3 (C-7''), 29.7 (C-2'), 29.0 (C-8''), 26.8 (C-5/C-6/C-7), 26.42 (C-3'), 26.35 (C-5/C-6/C-7), 22.7 (C-9''), 22.4 (C-4'), 14.2 (C-10''), 14.1 (C-5'); (+)-HRESIMS $[M+H]^+$ *m/z* 333.3251 (calcd for $C_{22}H_{41}N_2$, 333.3264).

32: R_f (CH_2Cl_2/MeOH, 9:1) 0.57; IR (ATR) v_{max} 3375, 2958, 2927, 2853, 1560, 1449, 1379, 1162, 955 cm^{-1}; ^1H NMR ($CDCl_3$, 400 MHz) δ 6.77 (1H, br s, H-1''), 6.60 (1H, dd, *J* = 2.3, 2.3 Hz, H-4''), 6.21 (1H, dd, *J* = 2.3, 2.3 Hz, H-3''), 4.05–4.01 (1H, m, H-1), 3.83 (2H, t, *J* = 7.3 Hz, H_2-6''), 2.76–2.59 (2H, m, H_2-1'), 1.99–1.95 (2H, m, H_2-2), 1.76–1.65 (10H, m, H_2-4, H_2-5, H_2-7, H_2-2', H_2-7''), 1.58–1.52 (1H, m, H-3), 1.34–1.17 (8H, m, H_2-3', H_2-4', H_2-8'', H_2-9''), 1.15–1.08 (2H, m, H_2-6), 0.93–0.82 (8H, m, H_2-8, H_3-5', H_3-10''); ^{13}C NMR ($CDCl_3$, 100 MHz) δ 121.5 (C-4''), 120.6 (C-1''), 117.3 (C-2''), 107.9 (C-3''), 57.9 (C-1), 49.9 (C-6''), 44.9 (C-1'), 41.3 (C-2), 34.4 (C-4, C-8), 34.1 (C-3), 31.2 (C-7''), 29.2 (C-2'), 28.9 (C-8''), 26.6 (C-5/C-6/C-7), 26.3 (C-5/C-6/C-7), 26.1 (C-3'), 22.4 (C-9''), 22.3 (C-4'), 14.13 (C-10''), 14.06 (C-5'); (+)-HRESIMS $[M+H]^+$ *m/z* 333.3253 (calcd for $C_{22}H_{41}N_2$, 333.3264).

General procedure for protein modification

To a solution of lysozyme (0.429 mg, 0.03 μM) in water (75 μL) was added compound (0.15 μM) in MeOH (5 μL). Following incubation at 20 °C for 20 hours, the sample (20 μL) was diluted with MeOH with 0.5% formic acid (0.5 mL) and analysed by (+)-HRESIMS.

General procedure for SDS-PAGE

Running buffer (pH 8.3) 4 L
Tris 12.0 g
Glycine 57.6 g
SDS (10%) 40 mL

Resolving Buffer (pH 8.8) 1 L
Tris bas 181.7 g
SDS (10%) 40 mL

Stacking Buffer (pH 6.8) 1 L
Tris base 60.6 g
SDS (10%) 40 mL

Resolving gel 15%
Water 14.2 mL
30% acrylamide (29% acryl:1% bis-acryl) 30 mL

Stacking gel
Water 30 mL
30% acrylamide (29% acryl:1% bis-acryl) 6.6 mL

Gel run
The modified lysozyme solution (2 μL) was diluted 10-fold with water and mixed with dye (5 μL). The mixture was boiled at 92 °C for 3 minutes and cooled to room temperature. Samples were loaded onto the stacking gel and run at 300 volts at 30 mA through the resolving gel.

Staining and destaining
After electrophoresis, the stacking gel was discarded and the resolving gel was stained with a solution of 10% v/v acetic acid, 40% v/v EtOH, 0.1% w/v Coomassie blue for 1 hour on a shaker. The stain was removed and destained with a solution of acetic acid/EtOH/H_2O (2:8:10 v/v/v) for 1 hour on a

shaker. Destaining procedure was then repeated overnight to ensure complete removal of the stain followed by washing with water.

References

1. Stratakis, M.; Nencka, R.; Rabalakos, C.; Adam, W.; Krebs, O. *J. Org. Chem.* **2002**, *67*, 8758–8763.
2. Li, S.; Wang, X.; Liu, L.; Kang, J.; Wang, L.; Liu, H.; Ruan, C.; Nie, A.; Zheng, Z.; Xie, Y.; Zhao, G.; Xiao, J.; Hu, Y.; Zhong, W.; Cui, H.; Zhou, X. PCT/CN2004/001118, June 4, **2006**.
3. Ireland, C.; Faulkner, D. J. *Bioorganic Chem.* **1978**, *7*, 125–131.
