# Supplementary material for: Investigation of the electrophilic reactivity of the biologically active marine sesquiterpenoid onchidal and model compounds
Source: Beilstein J Org Chem. 2018 Aug 24;14:2229–35. doi: 10.3762/bjoc.14.197 (PMC6122400; doi:10.3762/bjoc.14.197)
Supplement: File 2 — 1H and 13C NMR spectra of synthesized compounds. [file Beilstein_J_Org_Chem-14-2229-s002.pdf]

Supporting Information File 2
for

Investigation of the electrophilic reactivity of the biologically active marine sesquiterpenoid onchidal and model compounds

Melissa M. Cadelis[1]* and Brent R. Copp[1]

Address: [1]School of Chemical Sciences, University of Auckland, Private Bag 92019, Auckland 1142, New Zealand

Email: Melissa M. Cadelis - m.cadelis@auckland.ac.nz

* Corresponding author

^{1}H and ^{13}C NMR spectra of synthesized compounds

Contents

^{1}H NMR spectrum in $CDCl_3$ (500 MHz) of *N*-(2-(2,2-dimethyl-6-methylenecyclohexyl)-1-(1-pentyl-1*H*-pyrrol-3-yl)ethyl)pentan-1-amine (**7**) S3

^{13}C NMR spectrum in $CDCl_3$ (125 MHz) of *N*-(2-(2,2-dimethyl-6-methylenecyclohexyl)-1-(1-pentyl-1*H*-pyrrol-3-yl)ethyl)pentan-1-amine (**7**) S4

^{1}H NMR spectrum in $CDCl_3$ (500 MHz) of *N*-(2-(2,2-dimethyl-6-methylenecyclohexyl)-1-(1-pentyl-1*H*-pyrrol-3-yl)ethyl)pentan-1-amine (**8**) S5

^{13}C NMR spectrum in $CDCl_3$ (125 MHz) of *N*-(2-(2,2-dimethyl-6-methylenecyclohexyl)-1-(1-pentyl-1*H*-pyrrol-3-yl)ethyl)pentan-1-amine (**8**) S6

^{1}H NMR spectrum in $CDCl_3$ (500 MHz) of *N*-(2-(2,2-dimethyl-6-methylenecyclohexyl)-1-(1-pentyl-1*H*-pyrrol-3-yl)ethyl)pentan-1-amine (**8** and **9**) S7

^{13}C NMR spectrum in $CDCl_3$ (125 MHz) of *N*-(2-(2,2-dimethyl-6-methylenecyclohexyl)-1-(1-pentyl-1*H*-pyrrol-3-yl)ethyl)pentan-1-amine (**8** and **9**) S8

^{1}H NMR spectrum in $CDCl_3$ (500 MHz) of (*E*)- 2-hexylidenesuccinaldehyde (**11**) S9

^{13}C NMR spectrum in $CDCl_3$ (125 MHz) of (*E*)-2-hexylidenesuccinaldehyde (**11**) S10

^{1}H NMR spectrum in $CDCl_3$ (500 MHz) of (1*E*,3*E*)-3-formylnona-1,3-dien-1-yl acetate (**13**) S11
^{13}C NMR spectrum in $CDCl_3$ (125 MHz) of (1*E*,3*E*)-3-formylnona-1,3-dien-1-yl acetate (**13**) S12
^{1}H NMR spectrum in $CDCl_3$ (400 MHz) of (*E*)-2-(2-cyclohexylethylidene)succinaldehyde (**15**) S13
^{13}C NMR spectrum in $CDCl_3$ (100 MHz) of (*E*)-2-(2-cyclohexylethylidene)succinaldehyde (**15**) S14
^{1}H NMR spectrum in $CDCl_3$ (400 MHz) of (*Z*-2-(2-cyclohexylethylidene)succinaldehyde (**16**) S15
^{13}C NMR spectrum in $CDCl_3$ (100 MHz) of (*Z*)-2-(2-cyclohexylethylidene)succinaldehyde (**16**) S16
^{1}H NMR spectrum in $CDCl_3$ (400 MHz) of (1*E*,3*E*)-5-cyclohexyl-3-formylpenta-1,3-dien-1-yl acetate (**17**) S17
^{13}C NMR spectrum in $CDCl_3$ (100 MHz) of (1*E*,3*E*)-5-cyclohexyl-3-formylpenta-1,3-dien-1-yl acetate (**17**) S18
^{1}H NMR spectrum in $CDCl_3$ (300 MHz) of (*E*)-diethyl 2-hexylidenesuccinate (**20**) S19
^{13}C NMR spectrum in $CDCl_3$ (75 MHz) of (*E*)-diethyl 2-hexylidenesuccinate (**20**) S20
^{1}H NMR spectrum in $CDCl_3$ (300 MHz) of (*Z*)-diethyl 2-hexylidenesuccinate (**21**) S21
^{13}C NMR spectrum in $CDCl_3$ (75 MHz) of (*Z*)-diethyl 2-hexylidenesuccinate (**21**) S22
^{1}H NMR spectrum in $CDCl_3$ (500 MHz) of (*E*)-2-hexylidenebutane-1,4-diol (**22**) S23
^{13}C NMR spectrum in $CDCl_3$ (125 MHz) of (*E*)-2-hexylidenebutane-1,4-diol (**22**) S24
^{1}H NMR spectrum in $CDCl_3$ (500 MHz) of (*Z*)-2-hexylidenebutane-1,4-diol (**23**) S25
^{13}C NMR spectrum in $CDCl_3$ (125 MHz) of (*Z*)-2-hexylidenebutane-1,4-diol (**23**) S26
^{1}H NMR spectrum in $CDCl_3$ (500 MHz) of (*E*)-diethyl 2-(2-cyclohexylethylidene)succinate (**25**) S27
^{13}C NMR spectrum in $CDCl_3$ (125 MHz) of (*E*)-diethyl 2-(2-cyclohexylethylidene)succinate (**25**) S28
^{1}H NMR spectrum in $CDCl_3$ (500 MHz) of (*Z*)-diethyl 2-(2-cyclohexylethylidene)succinate (**26**) S29
^{13}C NMR spectrum in $CDCl_3$ (125 MHz) of (*Z*)-diethyl 2-(2-cyclohexylethylidene)succinate (**26**) S30
^{1}H NMR spectrum in $CDCl_3$ (500 MHz) of (*E*)-2-(2-cyclohexylethylidene)butane-1,4-diol (**27**) S31
^{13}C NMR spectrum in $CDCl_3$ (125 MHz) of (*E*)-2-(2-cyclohexylethylidene)butane-1,4-diol (**27**) S32
^{1}H NMR spectrum in $CDCl_3$ (500 MHz) of (*Z*)-2-(2-cyclohexylethylidene)butane-1,4-diol (**28**) S33
^{13}C NMR spectrum in $CDCl_3$ (125 MHz) of (*Z*)-2-(2-cyclohexylethylidene)butane-1,4-diol (**28**) S34
^{1}H NMR spectrum in $CDCl_3$ (400 MHz) of *N*-pentyl-1-(1-pentyl-1*H*-pyrrol-3-yl)hexan-1-amine (**29**) S35
^{1}H NMR spectrum in $CDCl_3$ (500 MHz) of *N*-pentyl-1-(1-pentyl-1*H*-pyrrol-3-yl)hexan-1-amine (**30**) S36
^{13}C NMR spectrum in $CDCl_3$ (125 MHz) of *N*-pentyl-1-(1-pentyl-1*H*-pyrrol-3-yl)hexan-1-amine (**30**) S37
^{1}H NMR spectrum in $CDCl_3$ (500 MHz) of *N*-(2-cyclohexyl-1-(1-pentyl-1*H*-pyrrol-3-yl)ethyl)pentan-1-amine (**31**) S38
^{13}C NMR spectrum in $CDCl_3$ (125 MHz) of *N*-(2-cyclohexyl-1-(1-pentyl-1*H*-pyrrol-3-yl)ethyl)pentan-1-amine (**31**) S39
^{1}H NMR spectrum in $CDCl_3$ (400 MHz) of *N*-(2-cyclohexyl-1-(1-pentyl-1*H*-pyrrol-3-yl)ethyl)pentan-1-amine (**32**) S40
^{13}C NMR spectrum in $CDCl_3$ (100 MHz) of *N*-(2-cyclohexyl-1-(1-pentyl-1*H*-pyrrol-3-yl)ethyl)pentan-1-amine (**32**) S41

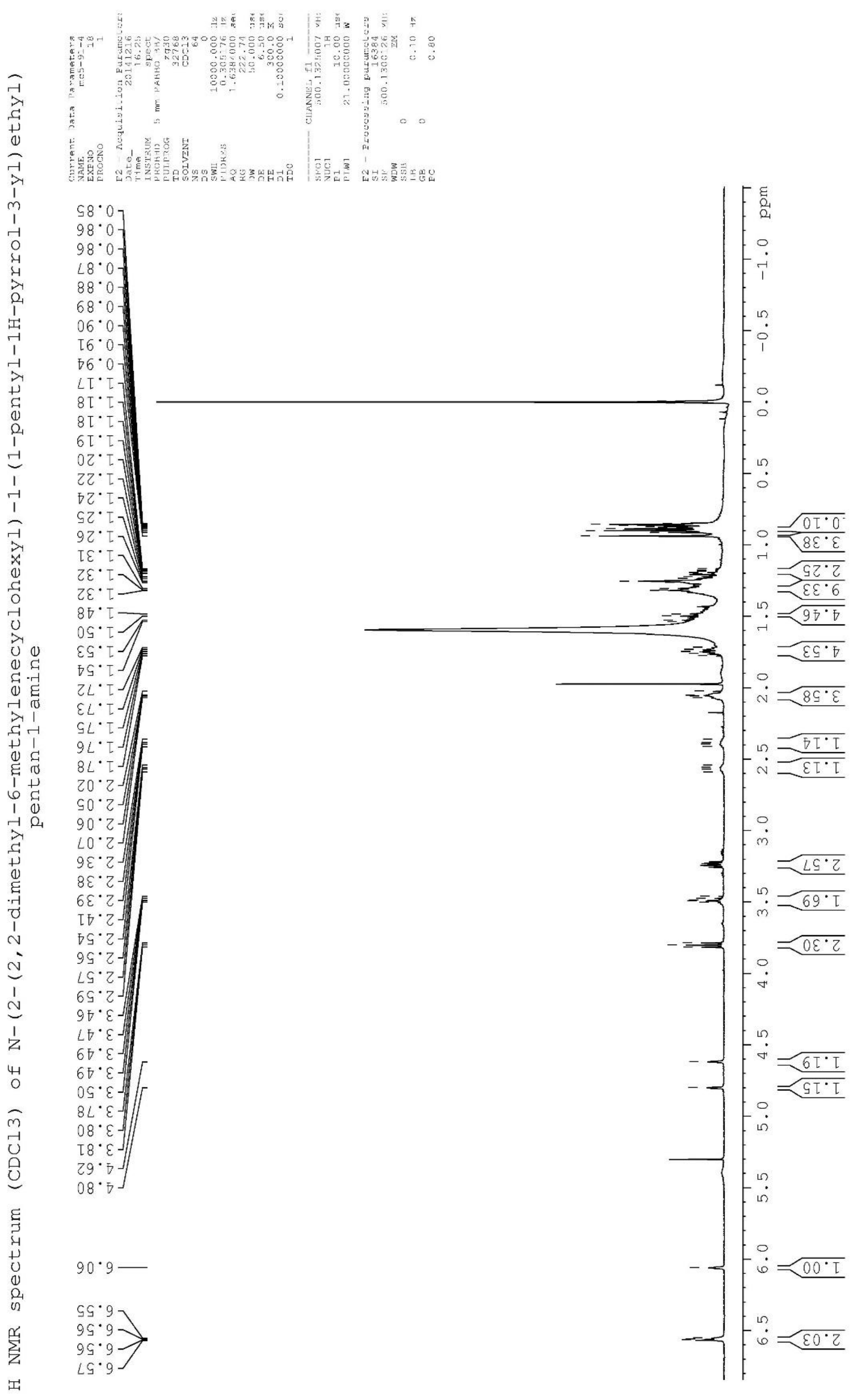

Figure 1 ^{1}H NMR spectrum in $CDCl_3$ (500 MHz) of *N*-(2-(2,2-dimethyl-6-methylenecyclohexyl)-1-(1-pentyl-1*H*-pyrrol-3-yl)ethyl)pentan-1-amine (**7**).

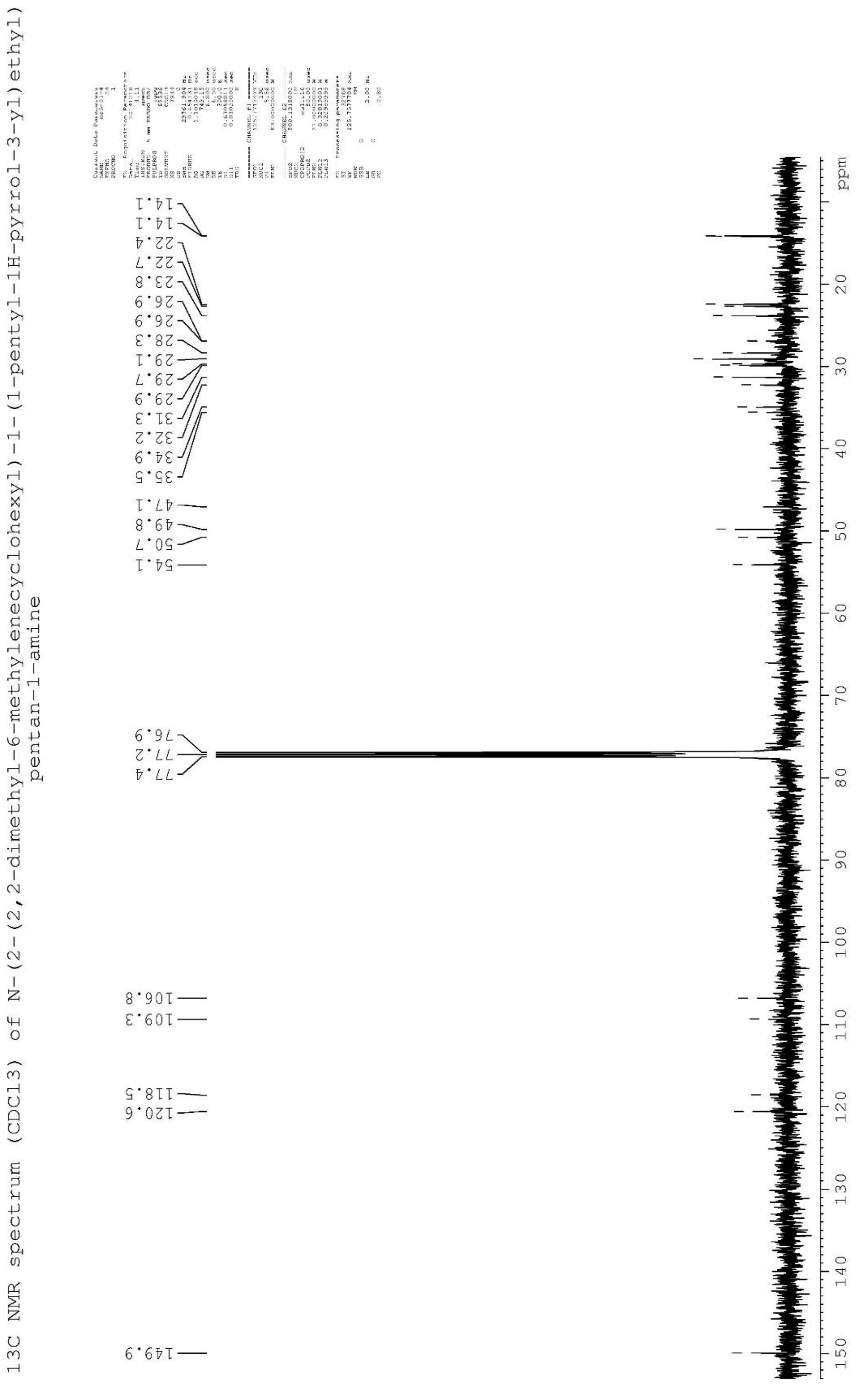

Figure 2 ^{13}C NMR spectrum in $CDCl_3$ (125 MHz) of *N*-(2-(2,2-dimethyl-6-methylenecyclohexyl)-1-(1-pentyl-1*H*-pyrrol-3-yl)ethyl)pentan-1-amine (**7**).

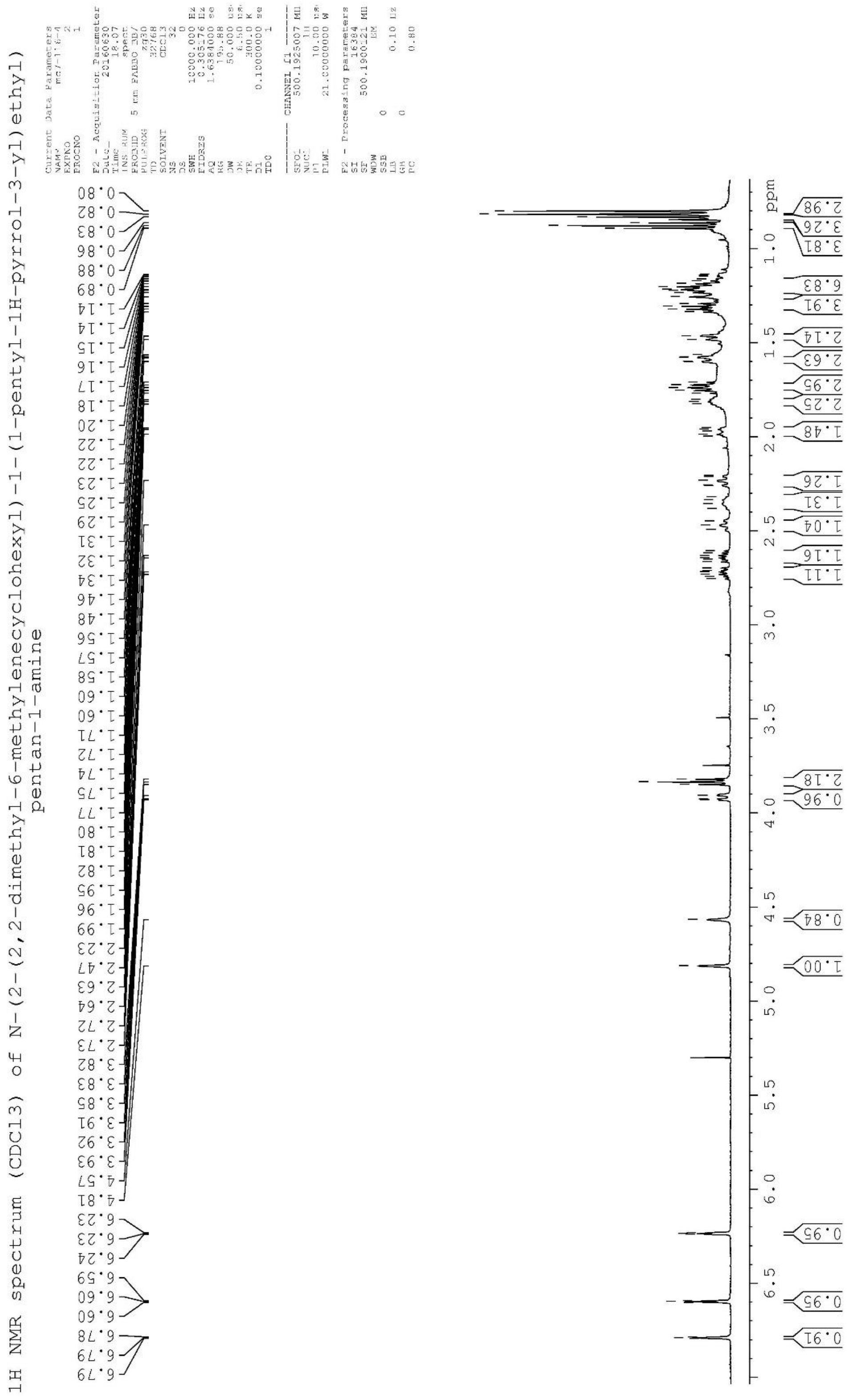

Figure 3 ^{1}H NMR spectrum in $CDCl_3$ (500 MHz) of *N*-(2-(2,2-dimethyl-6-methylenecyclohexyl)-1-(1-pentyl-1*H*-pyrrol-3-yl)ethyl)pentan-1-amine (**8**).

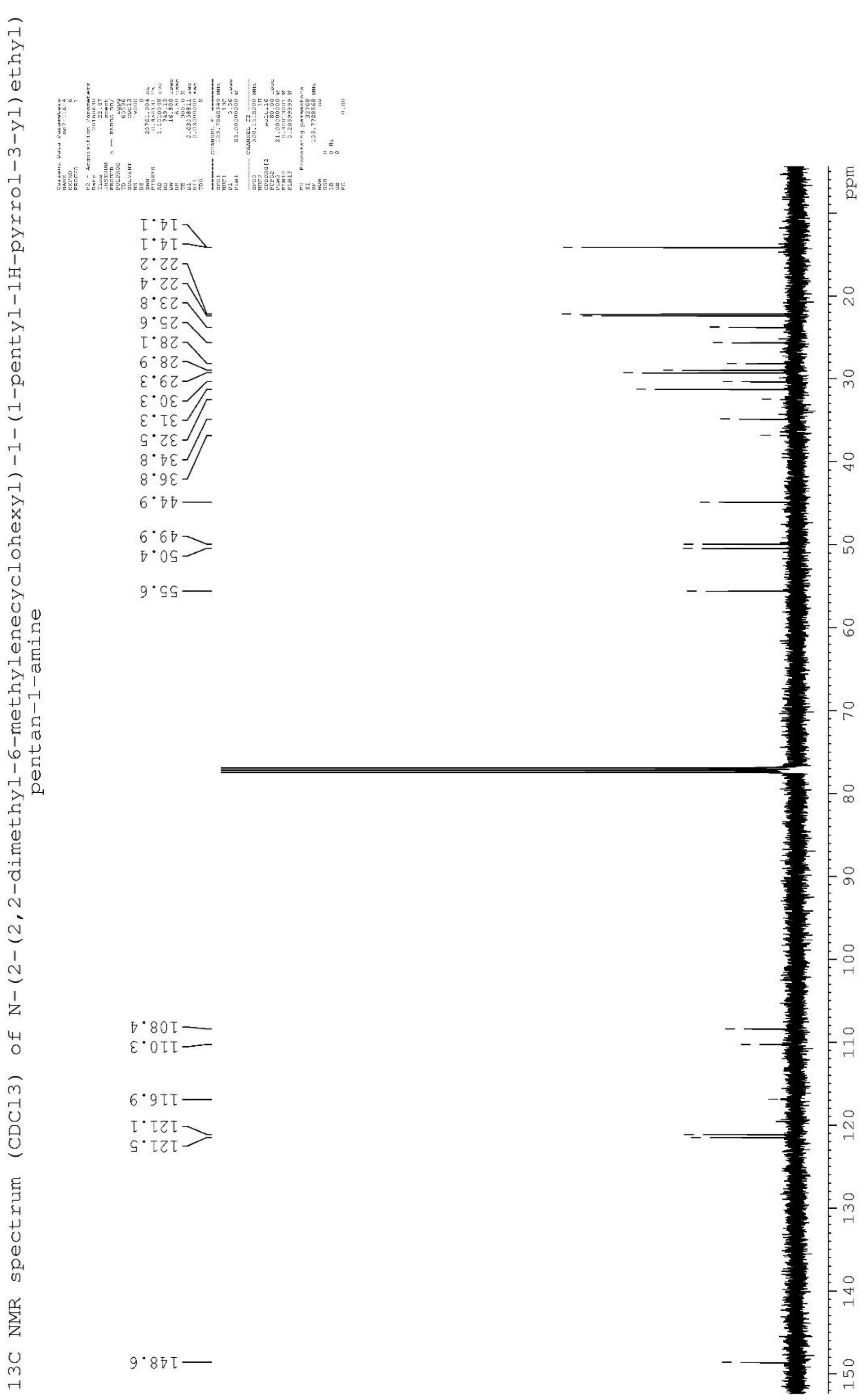

Figure 4 ^{13}C NMR spectrum in $CDCl_3$ (125 MHz) of *N*-(2-(2,2-dimethyl-6-methylenecyclohexyl)-1-(1-pentyl-1*H*-pyrrol-3-yl)ethyl)pentan-1-amine (**8**).

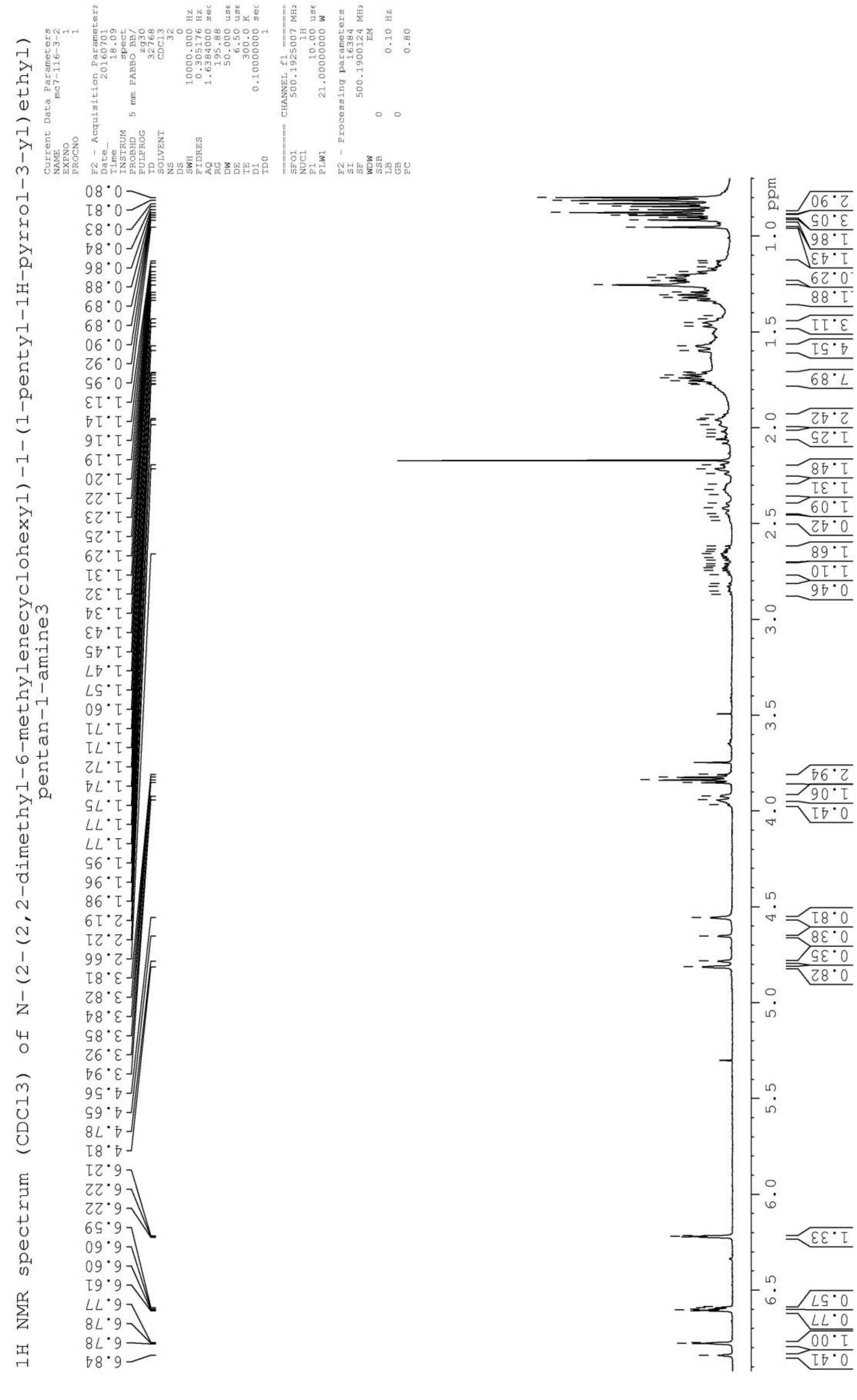

Figure 5 ^{1}H NMR spectrum in $CDCl_3$ (500 MHz) of *N*-(2-(2,2-dimethyl-6-methylenecyclohexyl)-1-(1-pentyl-1*H*-pyrrol-3-yl)ethyl)pentan-1-amine (**8** and **9**).

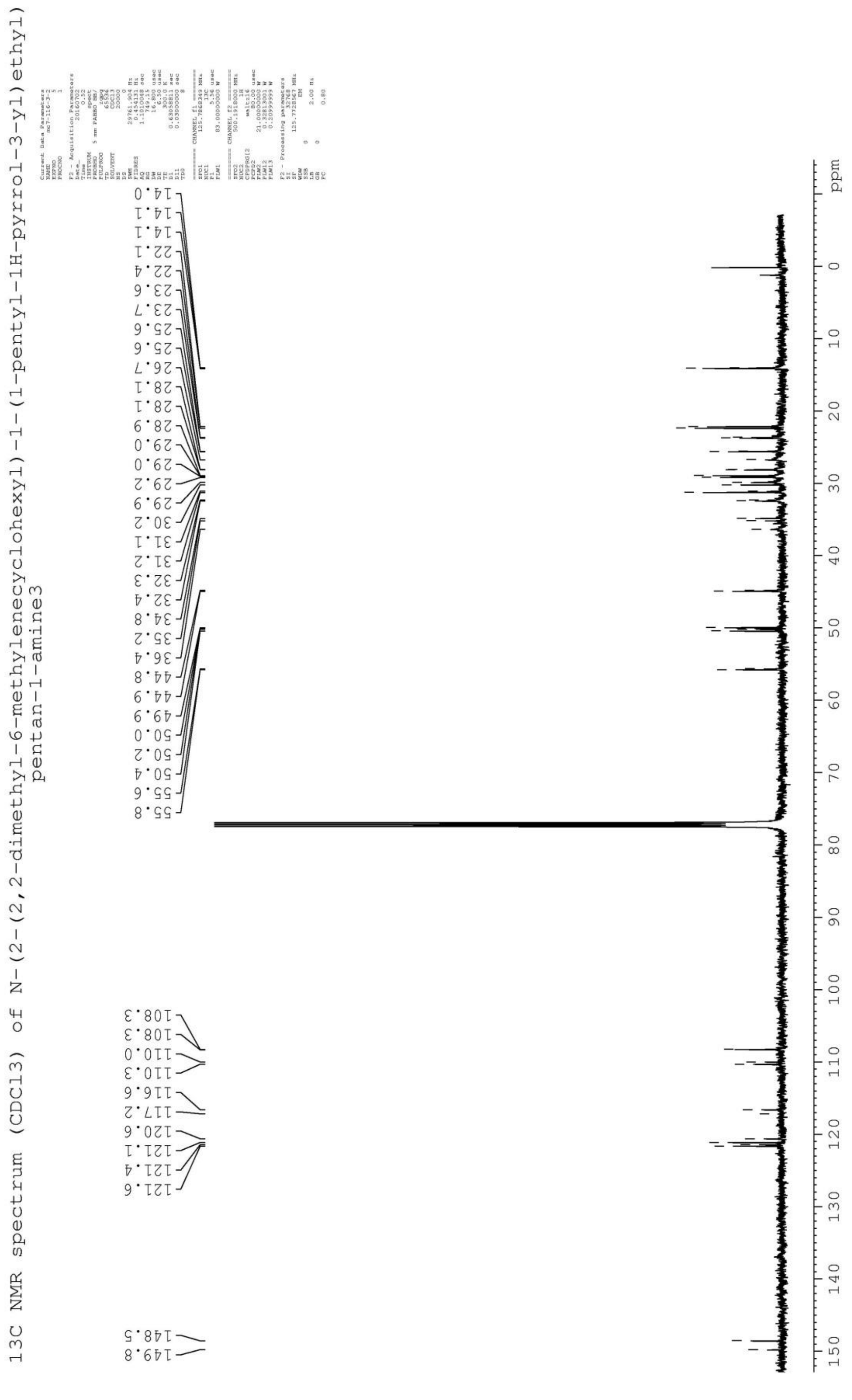

Figure 6 ^{13}C NMR spectrum in $CDCl_3$ (125 MHz) of *N*-(2-(2,2-dimethyl-6-methylenecyclohexyl)-1-(1-pentyl-1*H*-pyrrol-3-yl)ethyl)pentan-1-amine (**8** and **9**).

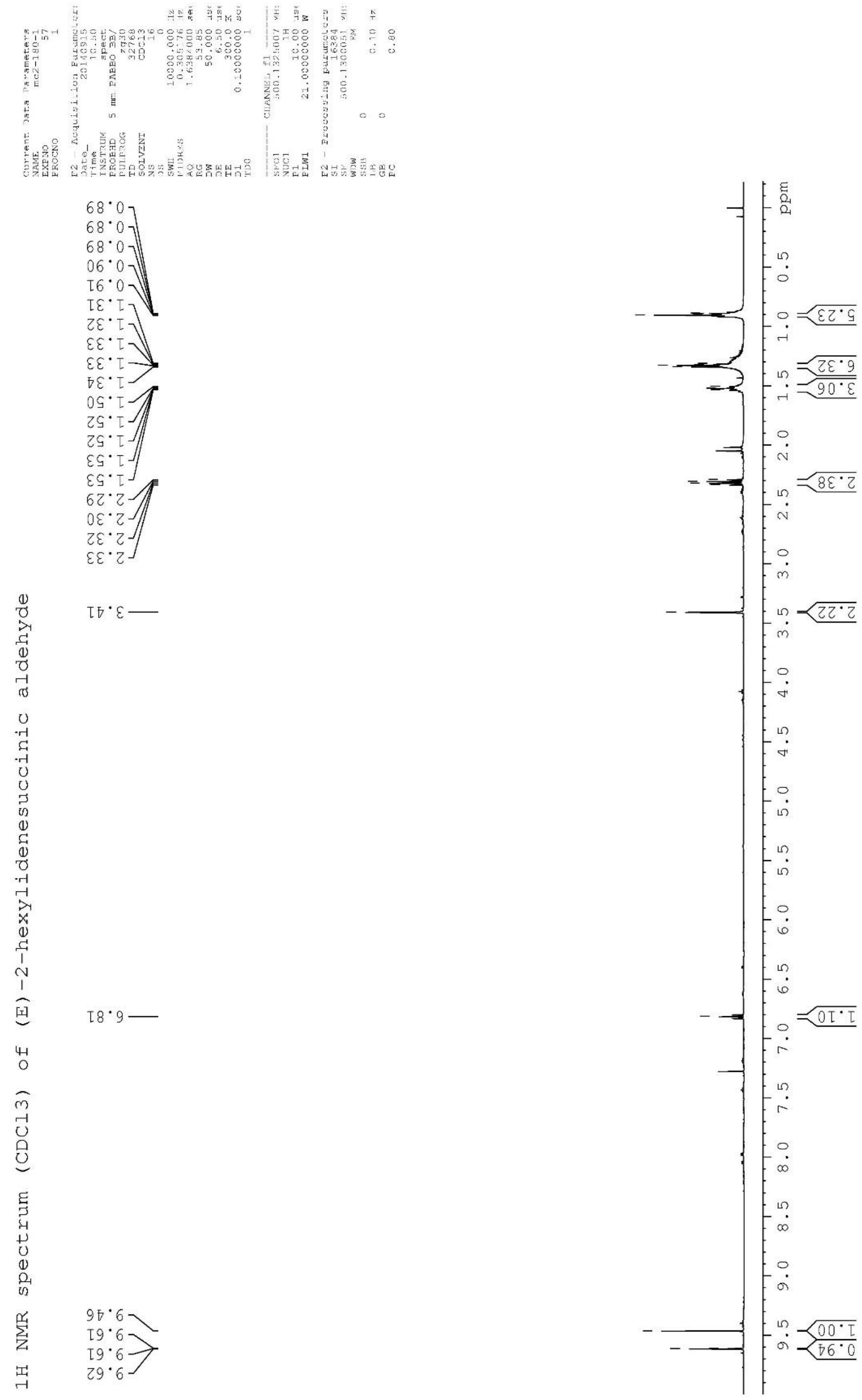

Figure 7 ^{1}H NMR spectrum in $CDCl_3$ (500 MHz) of (*E*)-2-hexylidenesuccinaldehyde (**11**).

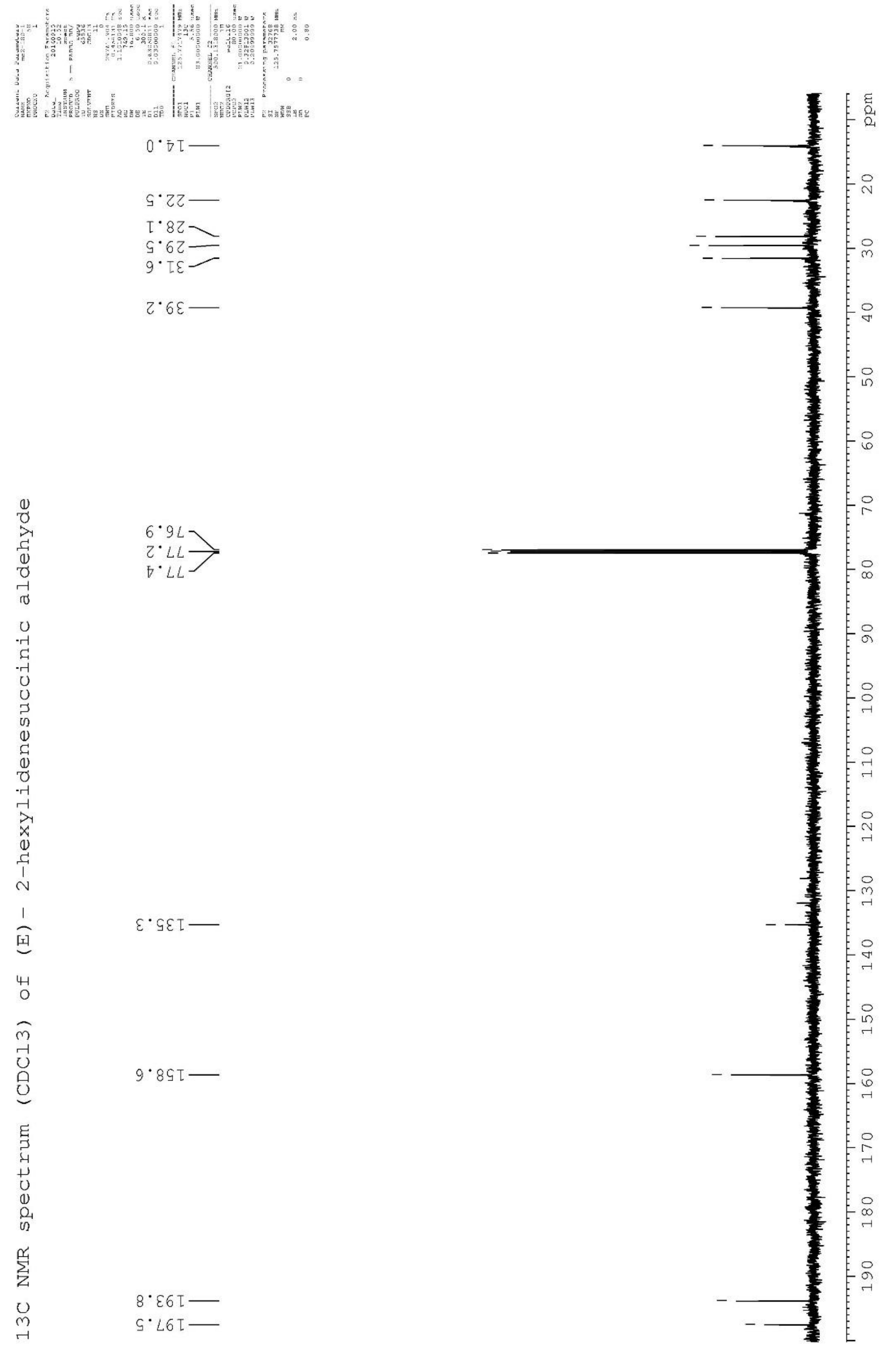

Figure 8 ^{13}C NMR spectrum in $CDCl_3$ (125 MHz) of (*E*)-2-hexylidenesuccinaldehyde (**11**).

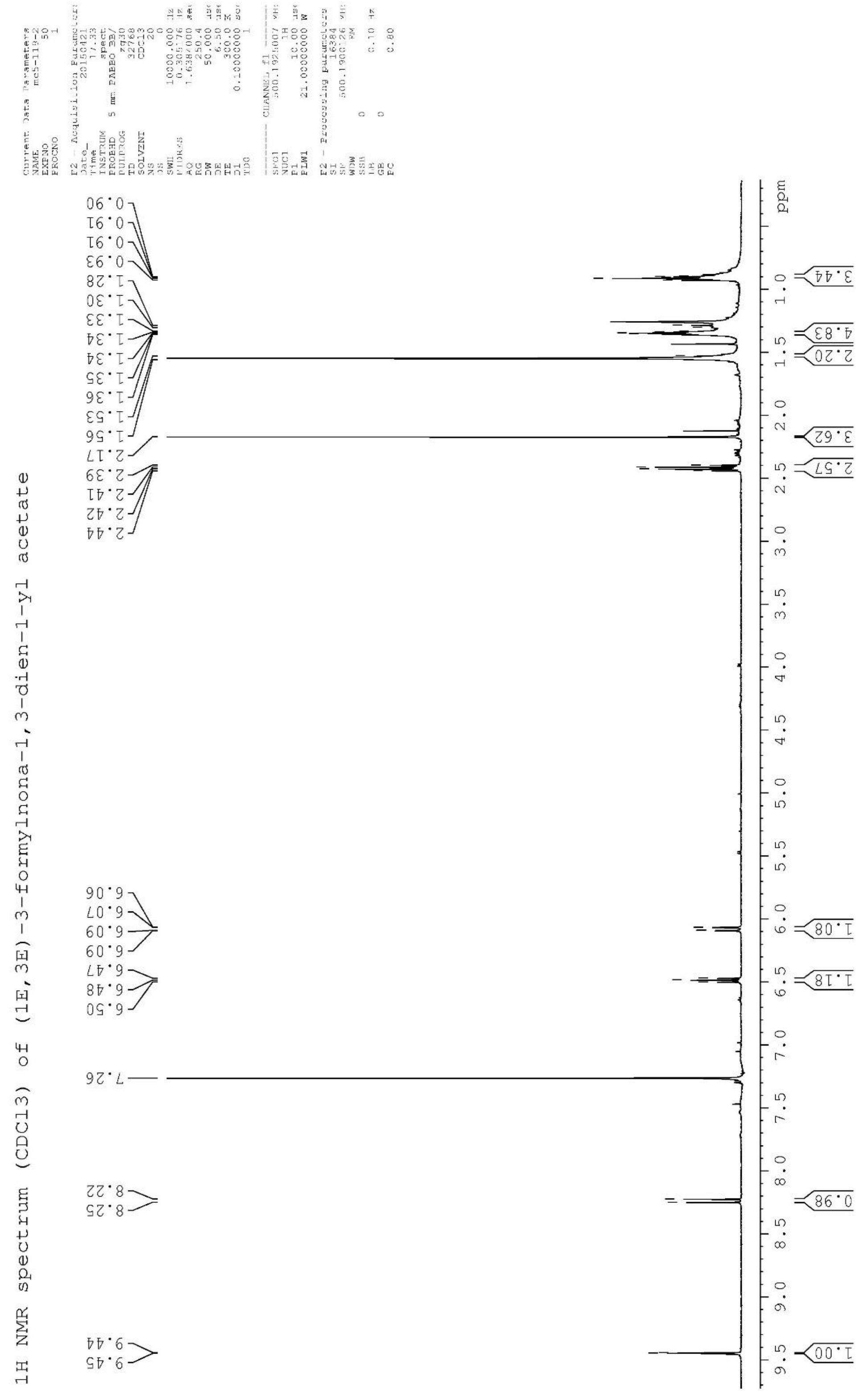

Figure 9 ^{1}H NMR spectrum in $CDCl_3$ (500 MHz) of (1*E*,3*E*)-3-formylnona-1,3-dien-1-yl acetate (**13**).

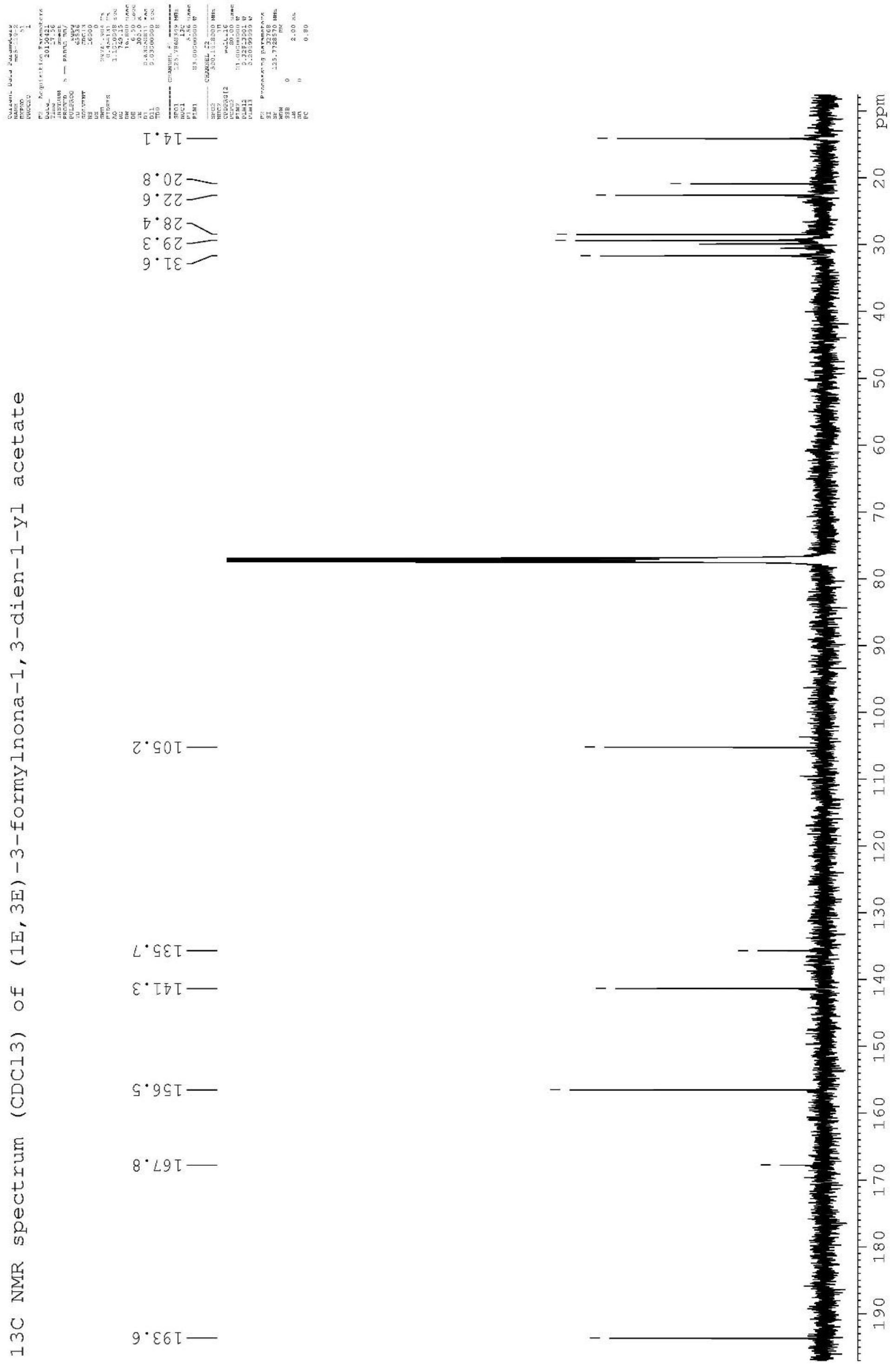

Figure 10 ^{13}C NMR spectrum in $CDCl_3$ (125 MHz) of (1*E*,3*E*)-3-formylnona-1,3-dien-1-yl acetate (**13**).

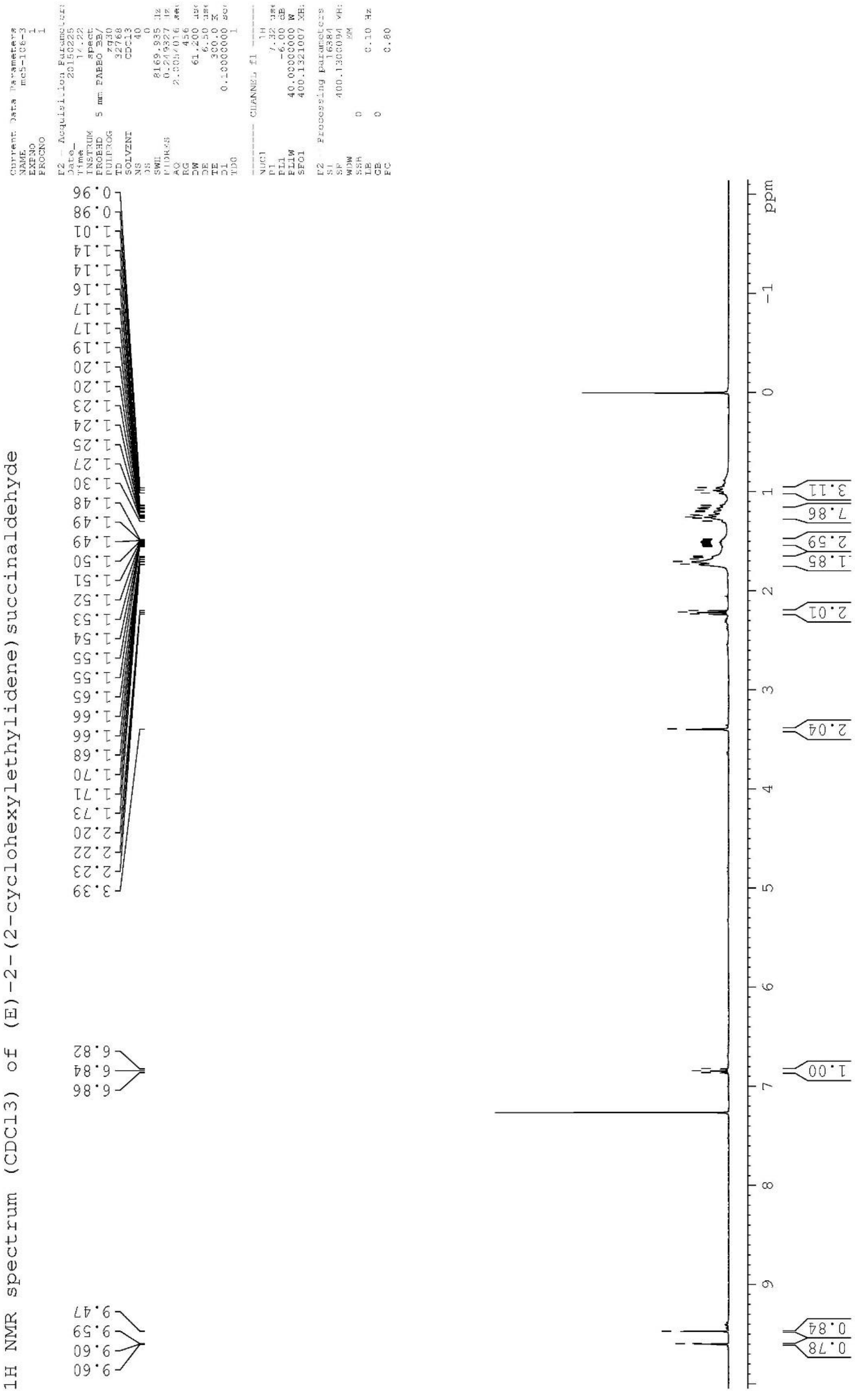

Figure 11 ^{1}H NMR spectrum in $CDCl_3$ (400 MHz) of (*E*)-2-(2-cyclohexylethylidene)succinaldehyde (**15**).

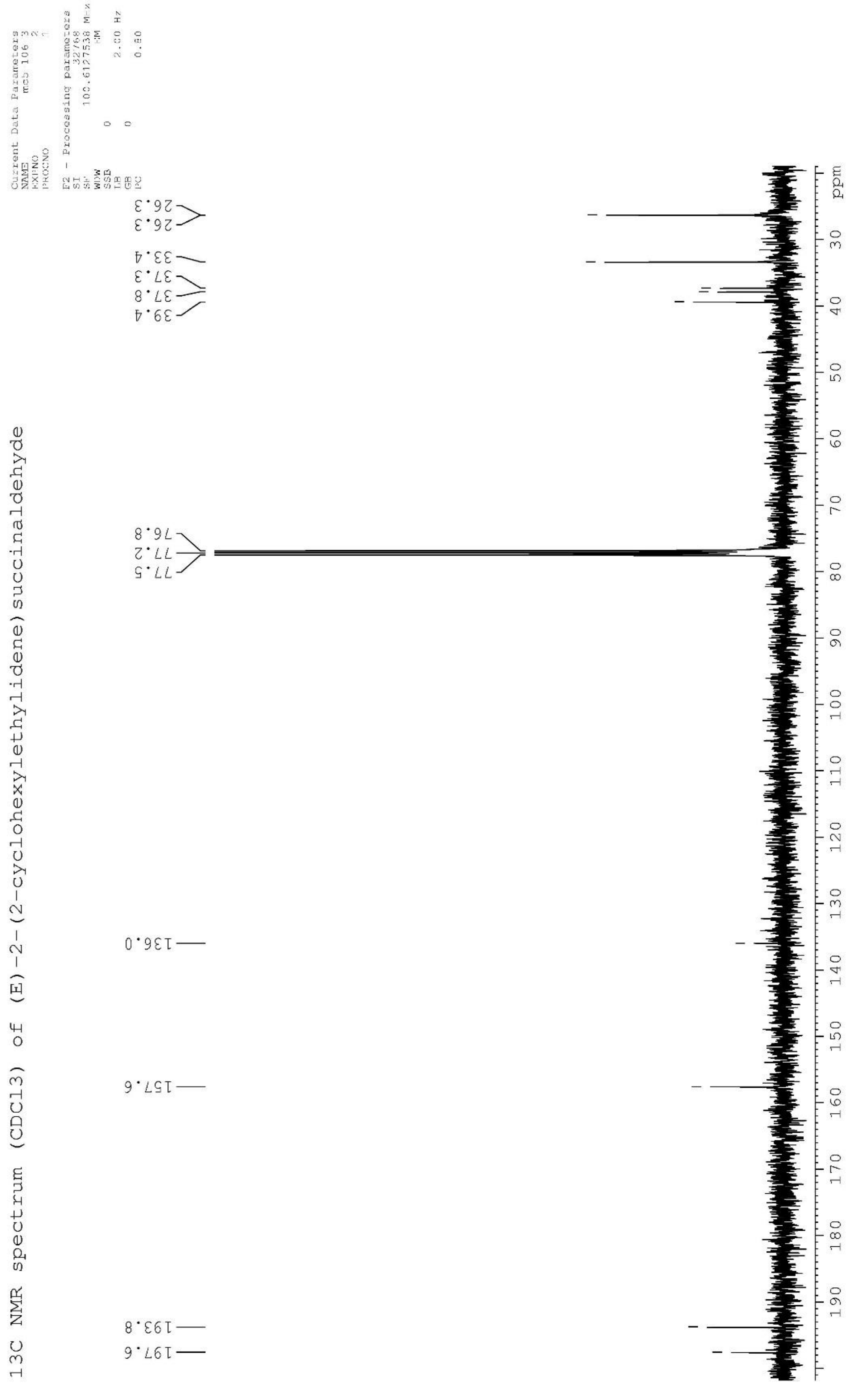

Figure 12 ^{13}C NMR spectrum in $CDCl_3$ (100 MHz) of (*E*)-2-(2-cyclohexylethylidene)succinaldehyde (**15**).

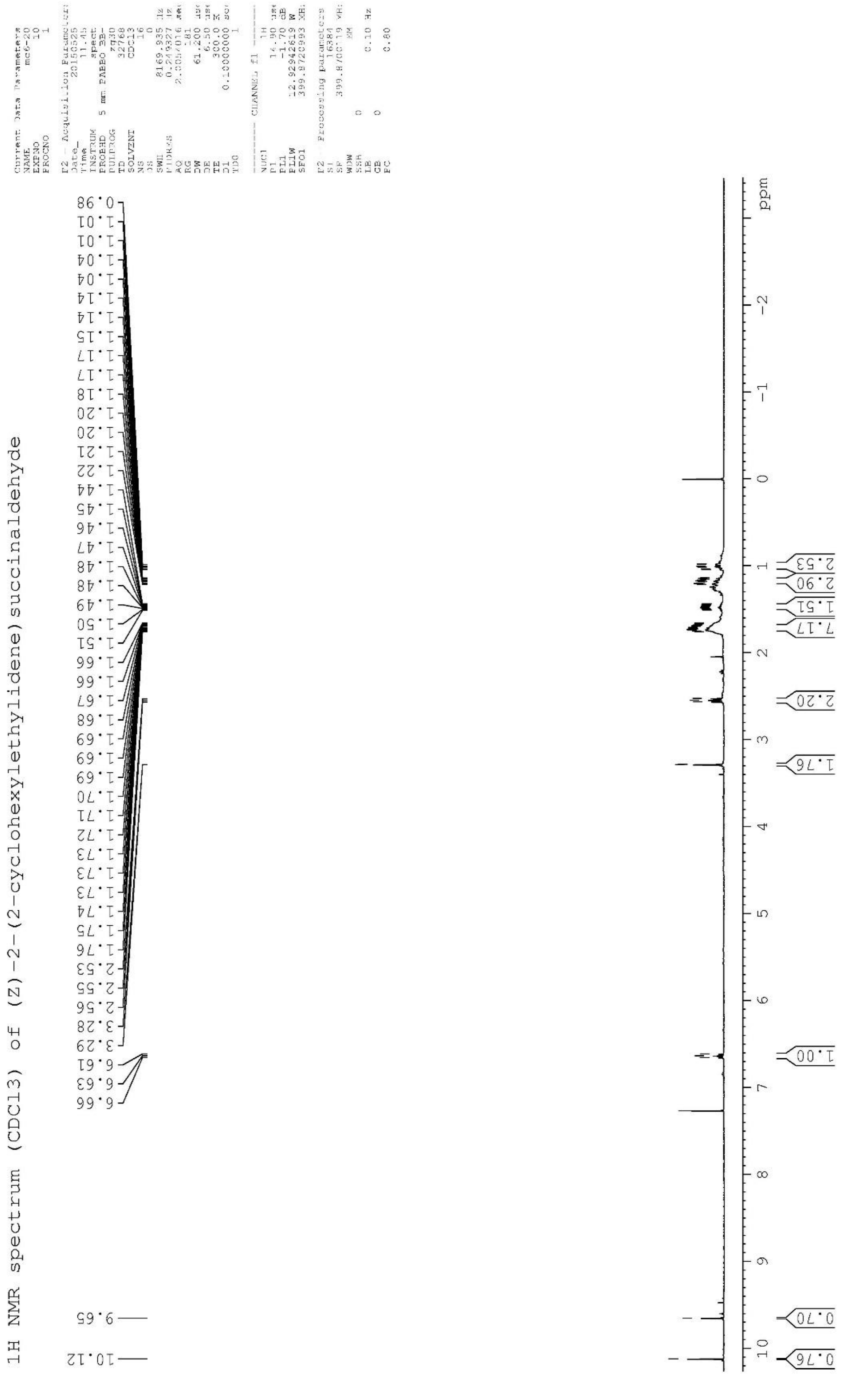

Figure 13 ^{1}H NMR spectrum in $CDCl_3$ (400 MHz) of (*Z*)-2-(2-cyclohexylethylidene)succinaldehyde (**16**).

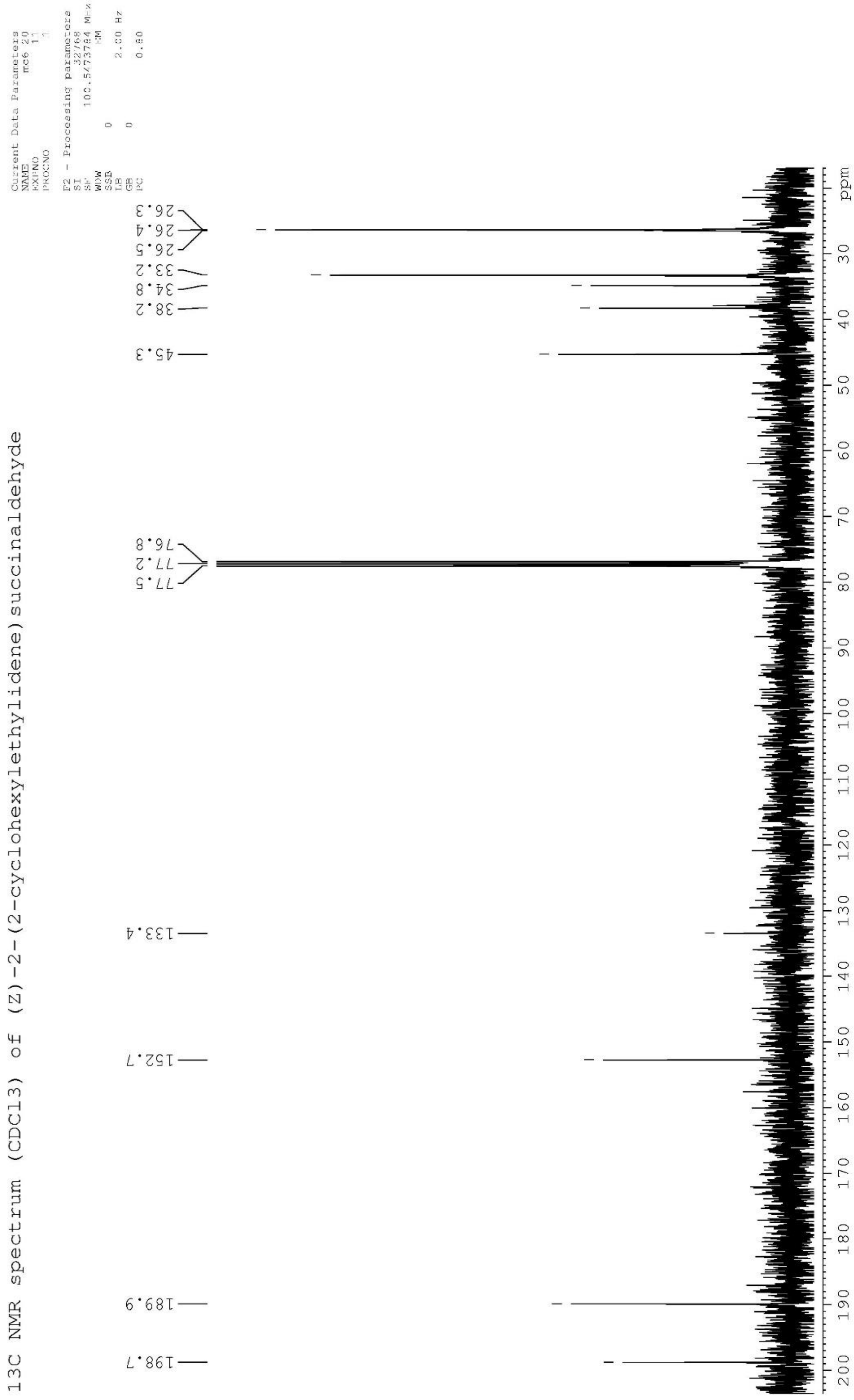

Figure 14 ^{13}C NMR spectrum in $CDCl_3$ (100 MHz) of (*Z*)-2-(2-cyclohexylethylidene)succinaldehyde (**16**).

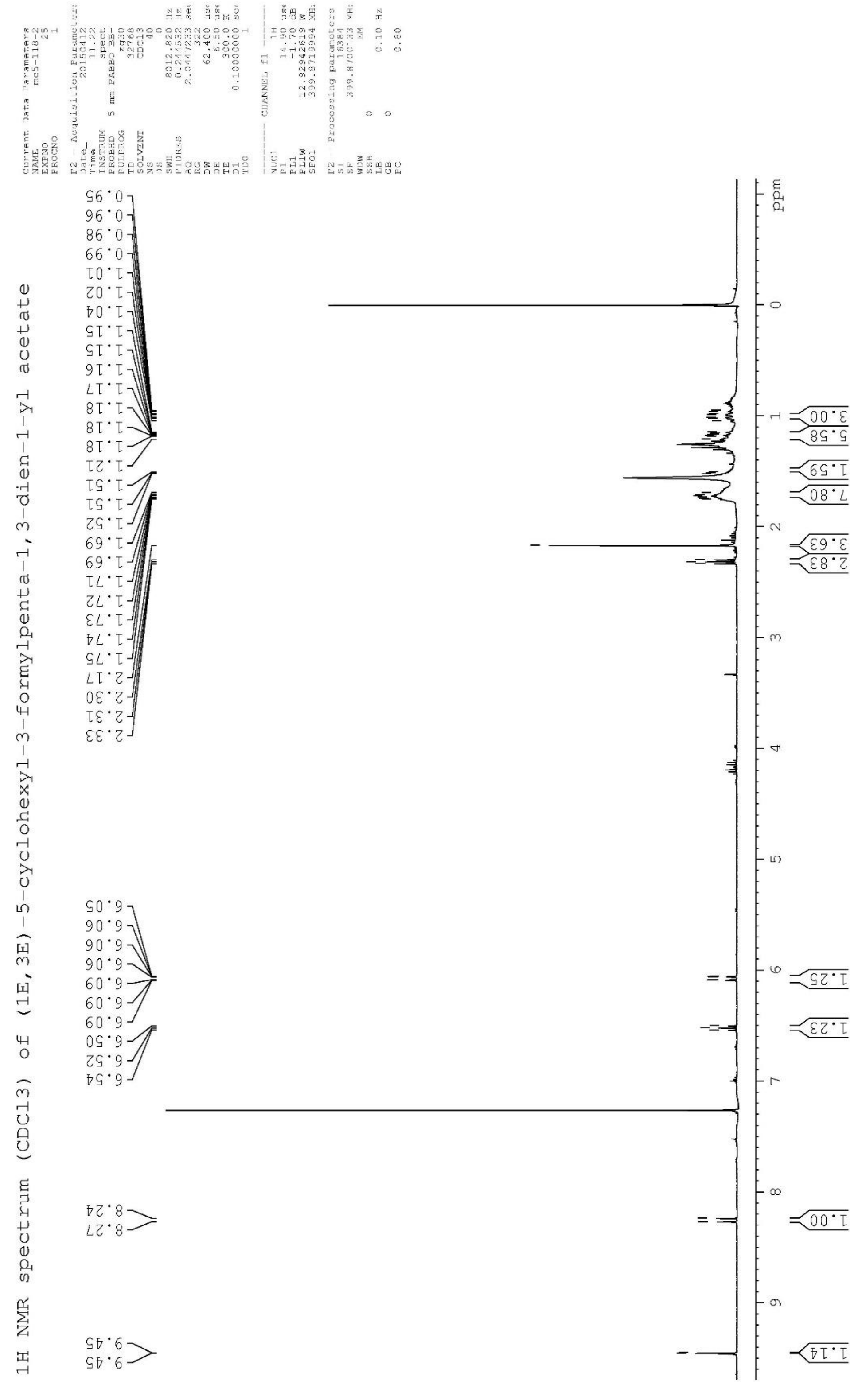

Figure 15 ^{1}H NMR spectrum in $CDCl_3$ (400 MHz) of (1*E*,3*E*)-5-cyclohexyl-3-formylpenta-1,3-dien-1-yl acetate (**17**).

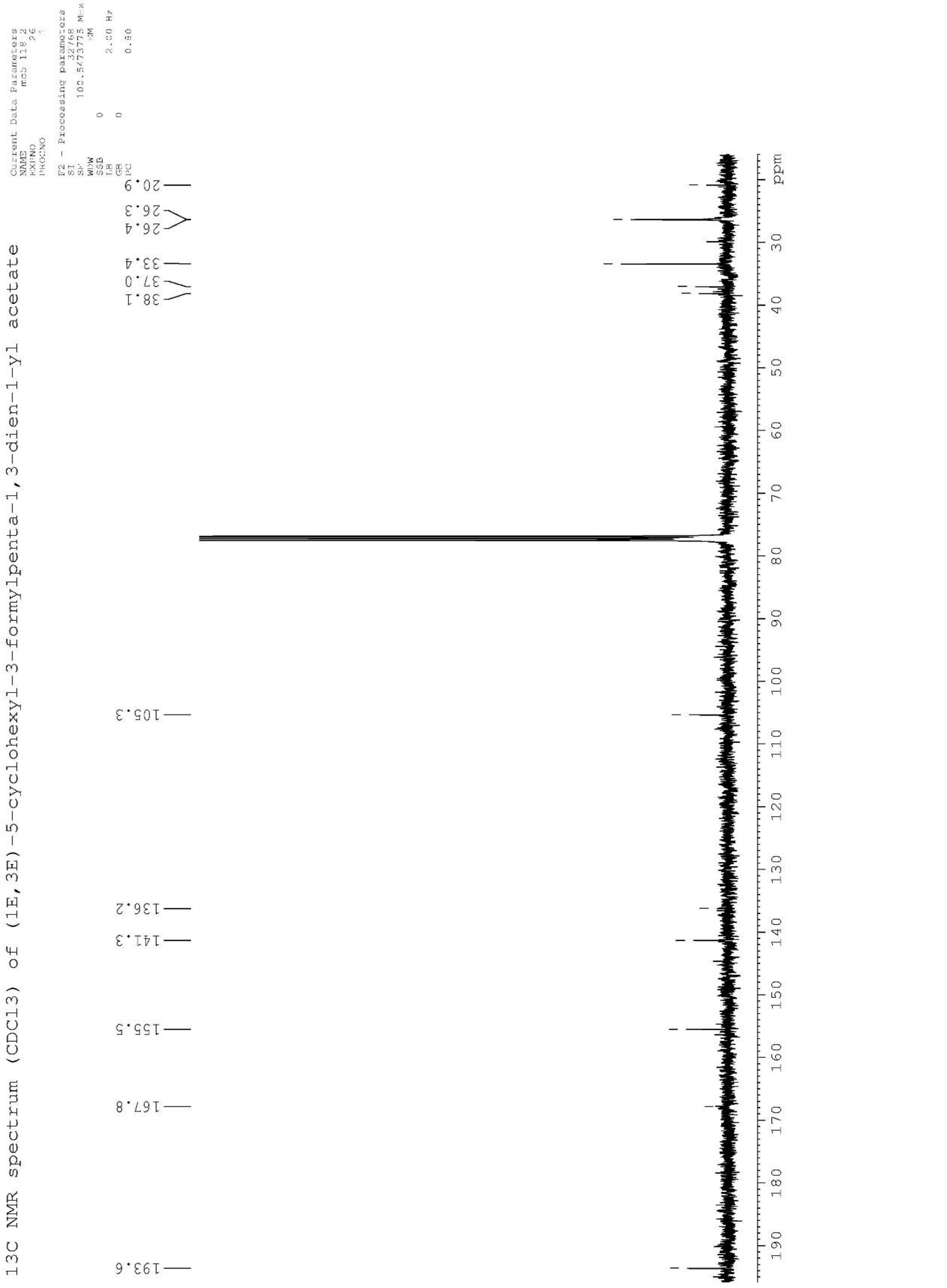

Figure 16 ^{13}C NMR spectrum in $CDCl_3$ (100 MHz) of (1*E*,3*E*)-5-cyclohexyl-3-formylpenta-1,3-dien-1-yl acetate (**17**).

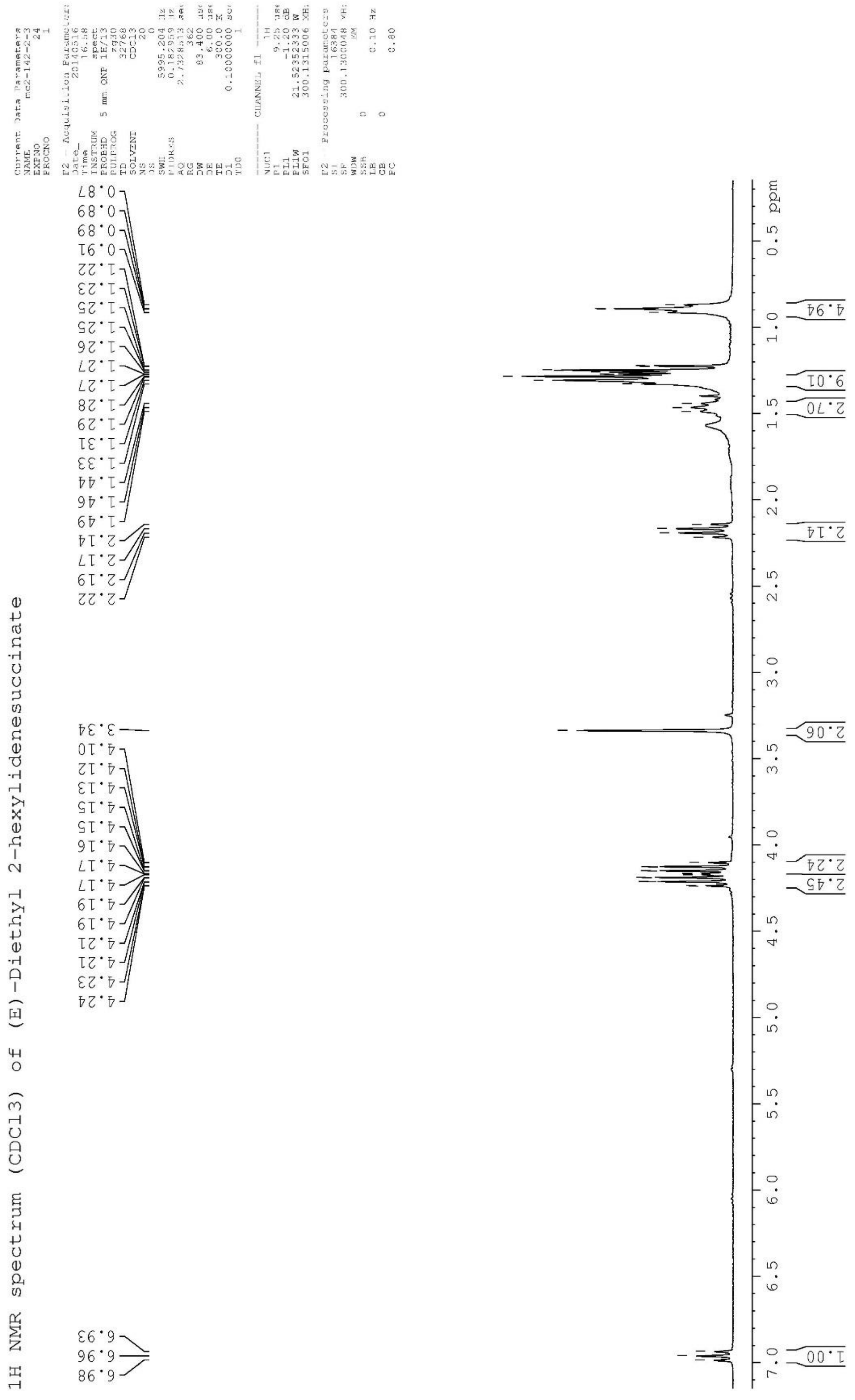

Figure 17 ^{1}H NMR spectrum in $CDCl_3$ (300 MHz) of (*E*)-diethyl 2-hexylidenesuccinate (**20**).

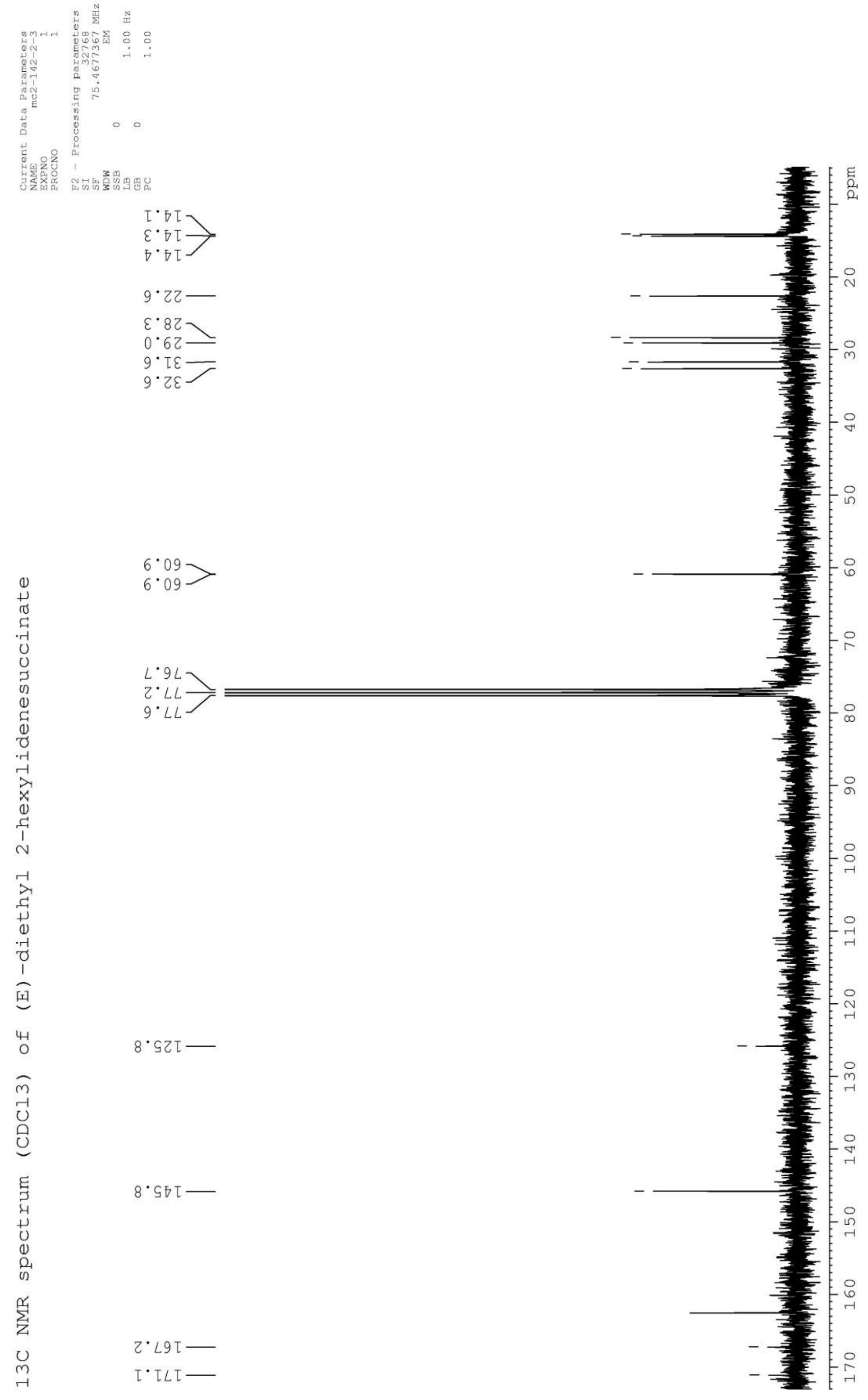

Figure 18 ^{13}C NMR spectrum in $CDCl_3$ (75 MHz) of (*E*)-diethyl 2-hexylidenesuccinate (**20**).

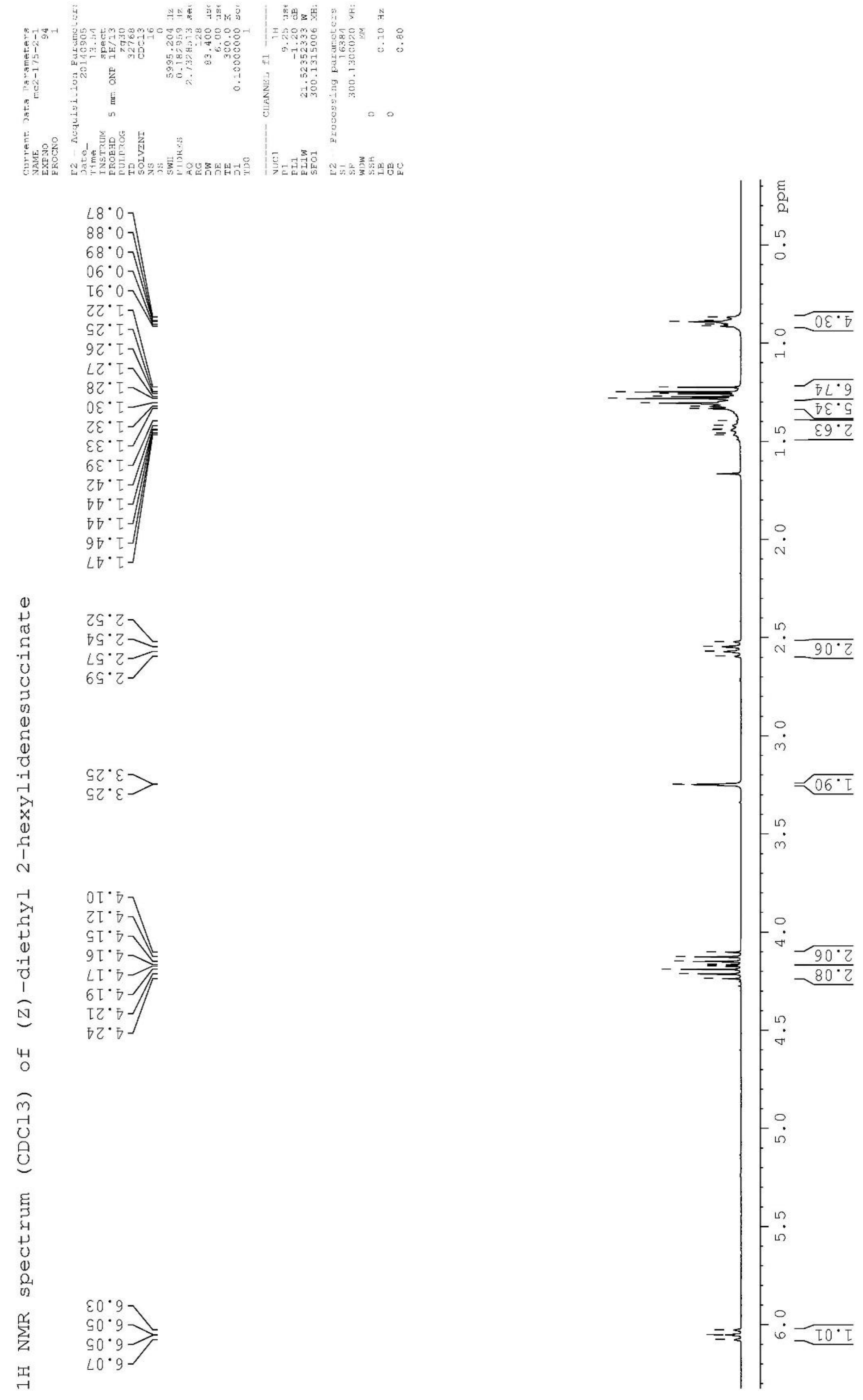

Figure 19 ^{1}H NMR spectrum in $CDCl_3$ (300 MHz) of (*Z*)-diethyl 2-hexylidenesuccinate (**21**).

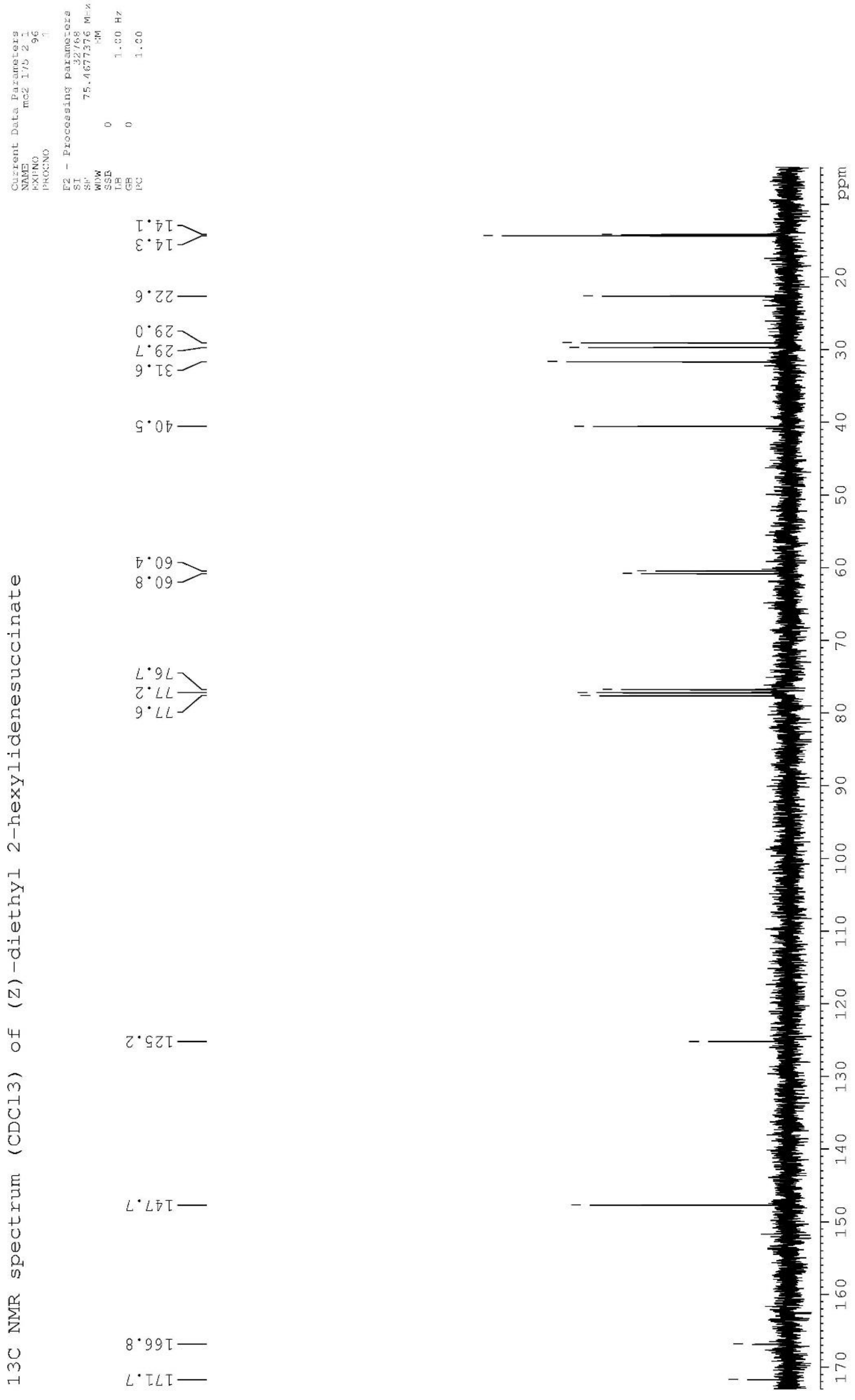

Figure 20 ^{13}C NMR spectrum in $CDCl_3$ (75 MHz) of (*Z*)-diethyl 2-hexylidenesuccinate (**21**).

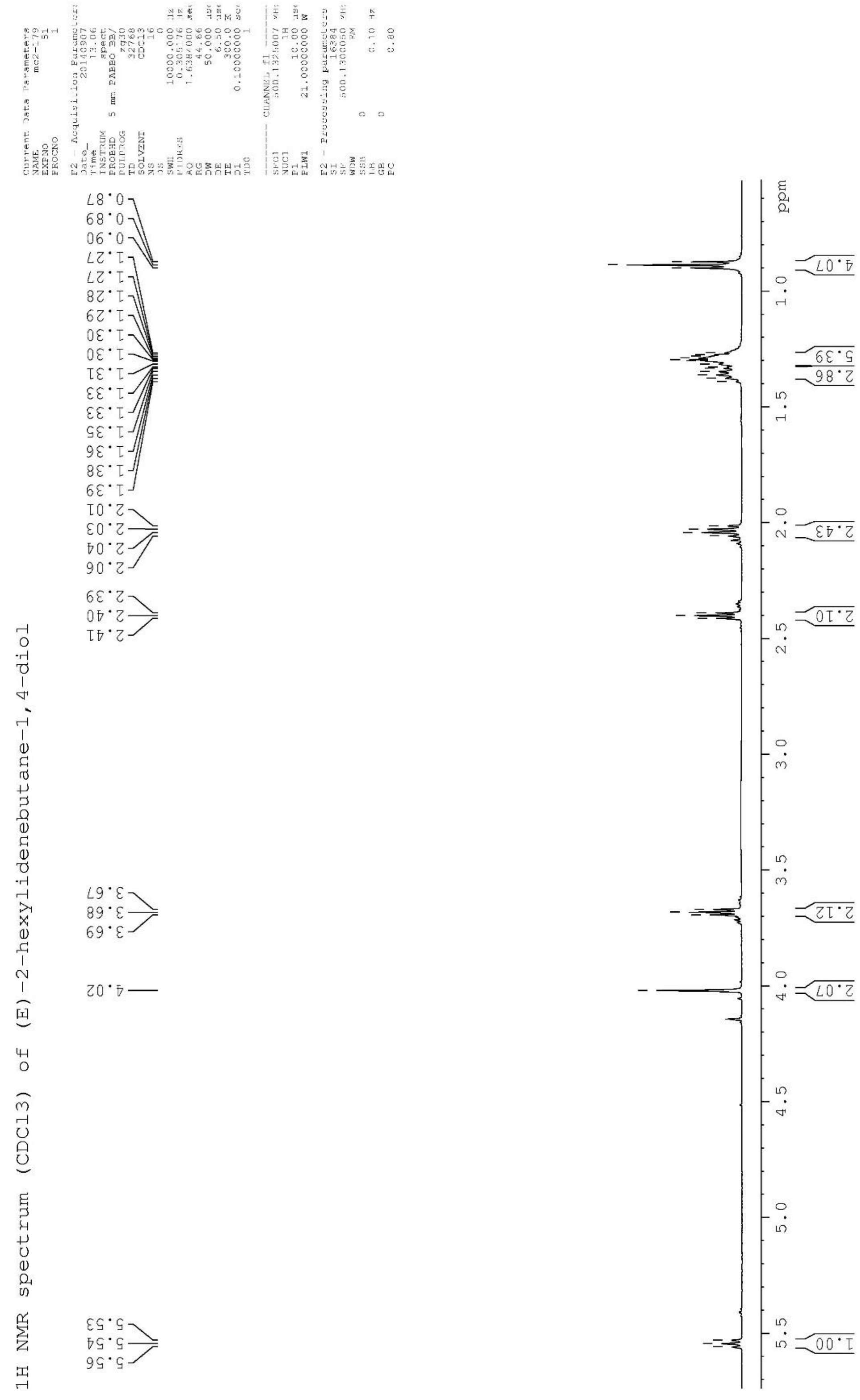

Figure 21 ^{1}H NMR spectrum in $CDCl_3$ (500 MHz) of (*E*)-2-hexylidenebutane-1,4-diol (**22**).

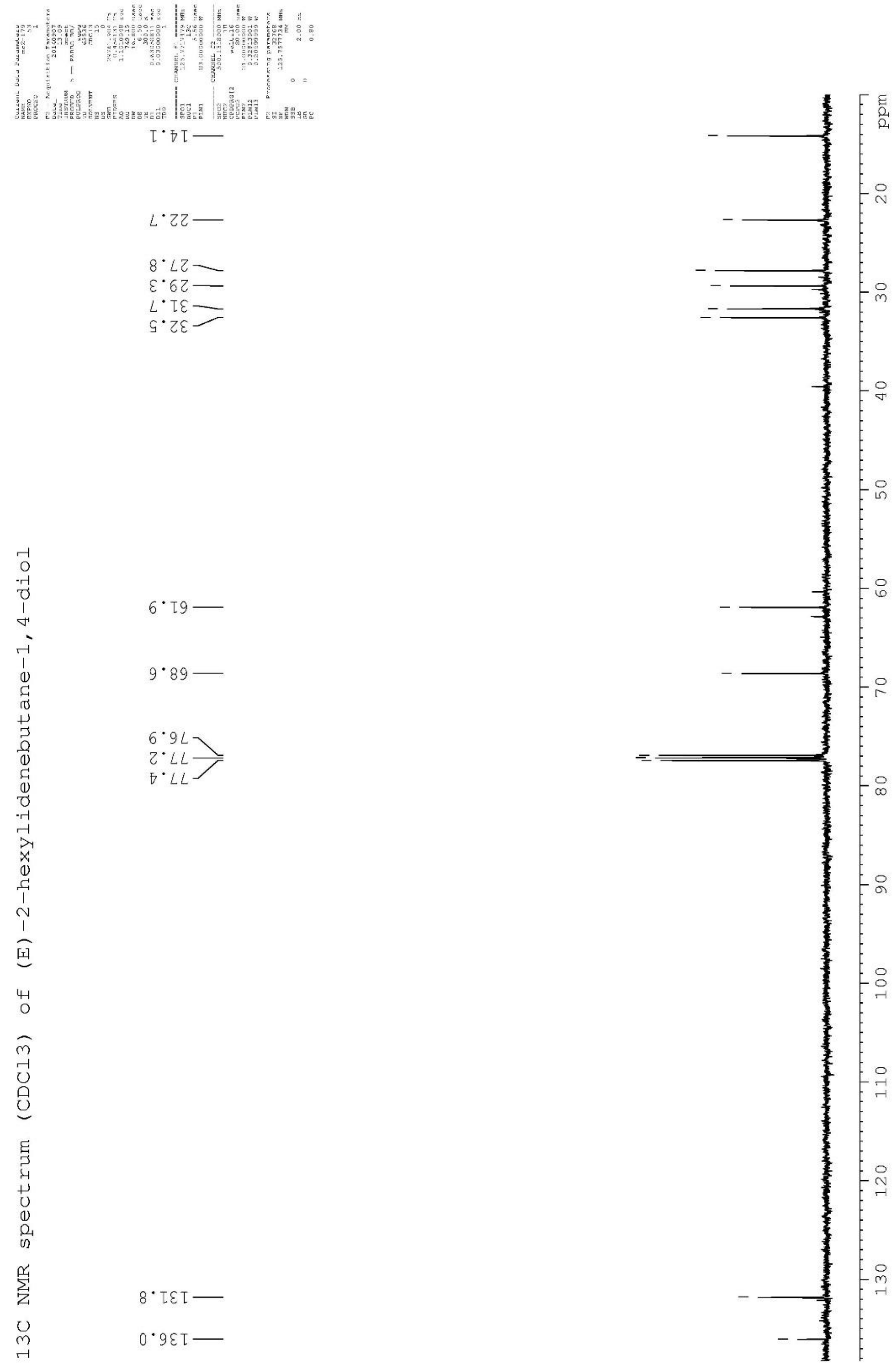

Figure 22 ^{13}C NMR spectrum in $CDCl_3$ (125 MHz) of (*E*)-2-hexylidenebutane-1,4-diol (**22**).

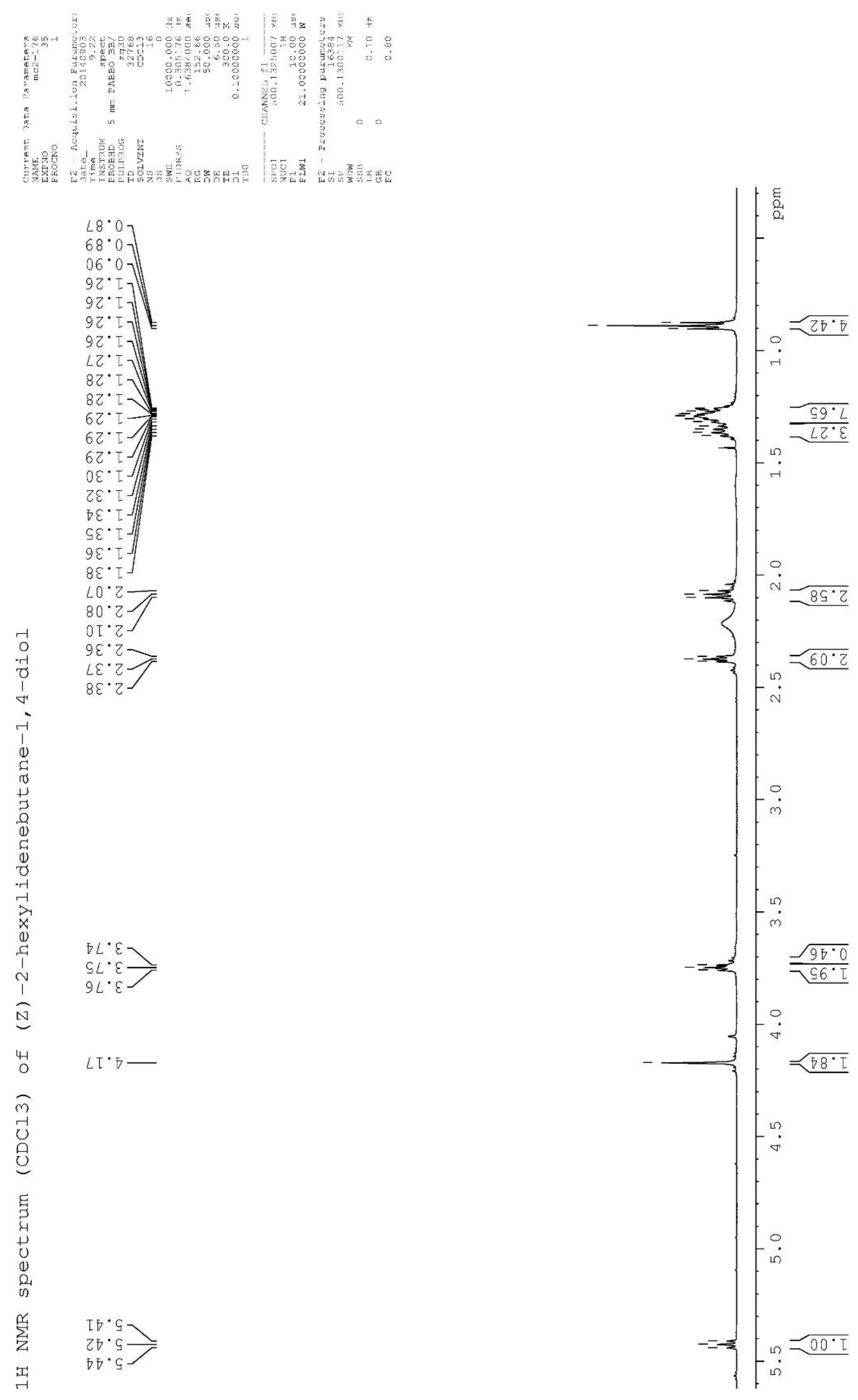

Figure 23 ^{1}H NMR spectrum in $CDCl_3$ (500 MHz) of (*Z*)-2-hexylidenebutane-1,4-diol (**23**).

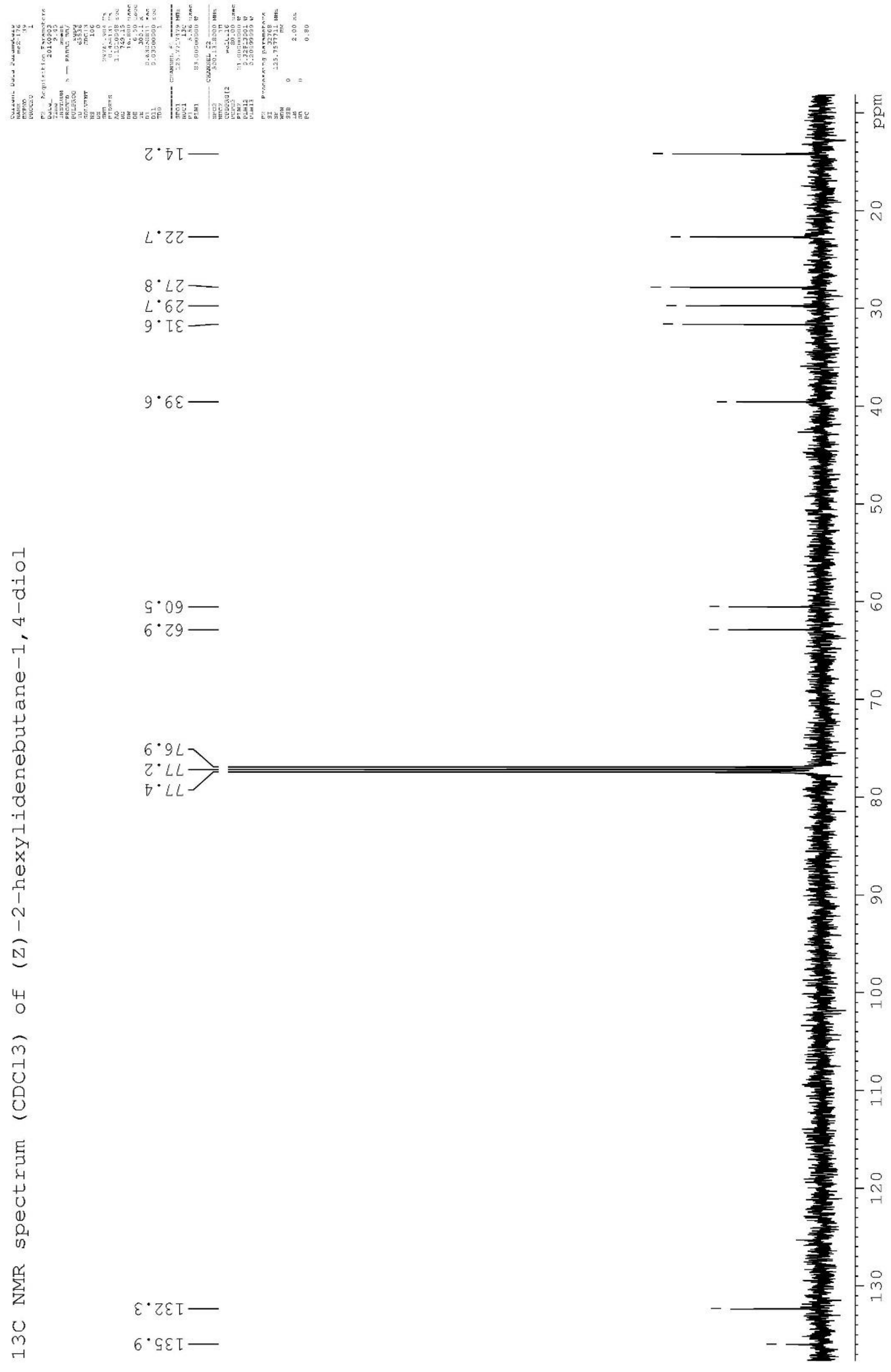

Figure 24 ^{13}C NMR spectrum in $CDCl_3$ (125 MHz) of (*Z*)-2-hexylidenebutane-1,4-diol (**23**).

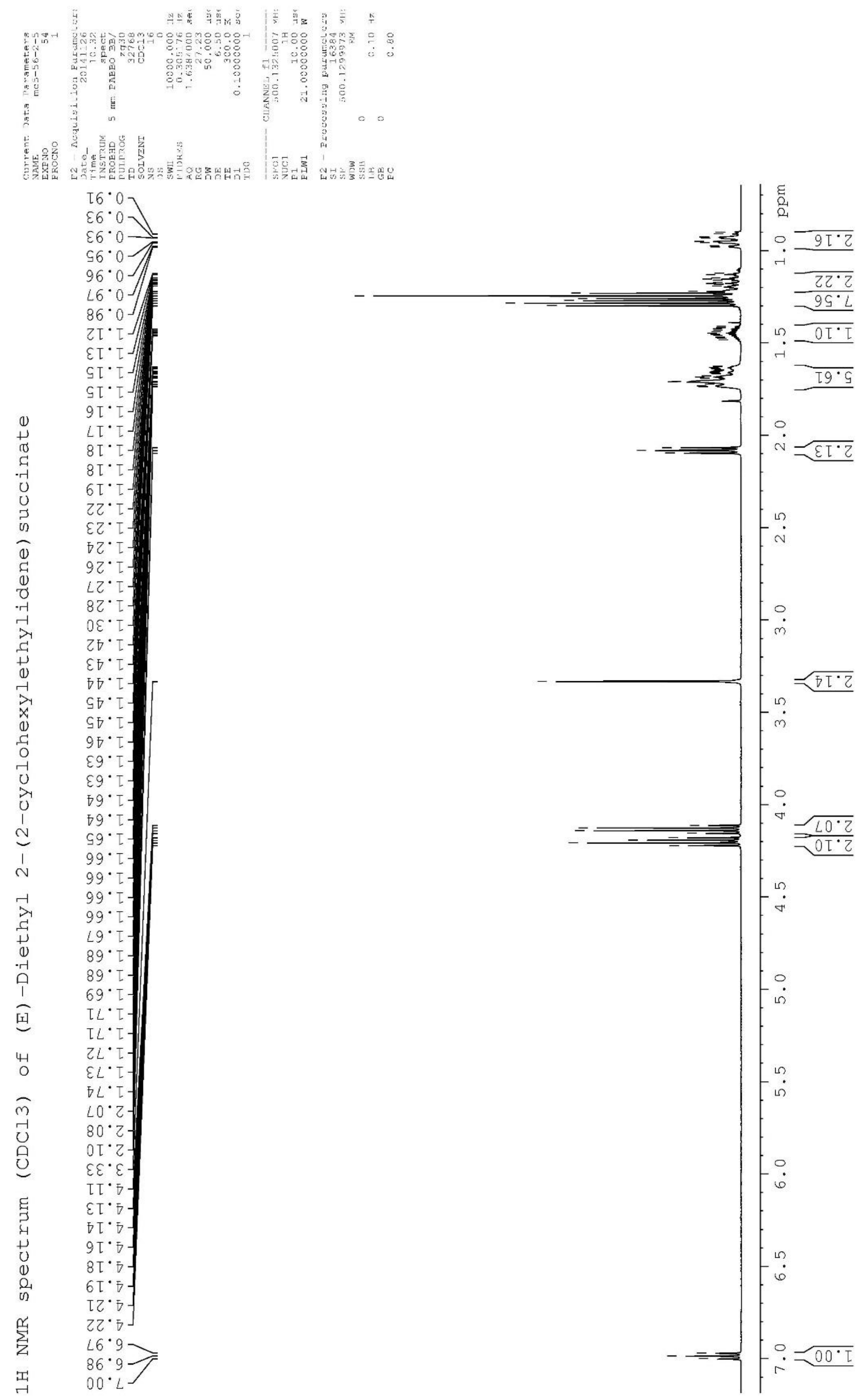

Figure 25 ^{1}H NMR spectrum in $CDCl_3$ (500 MHz) of (*E*)-diethyl 2-(2-cyclohexylethylidene)succinate (**25**).

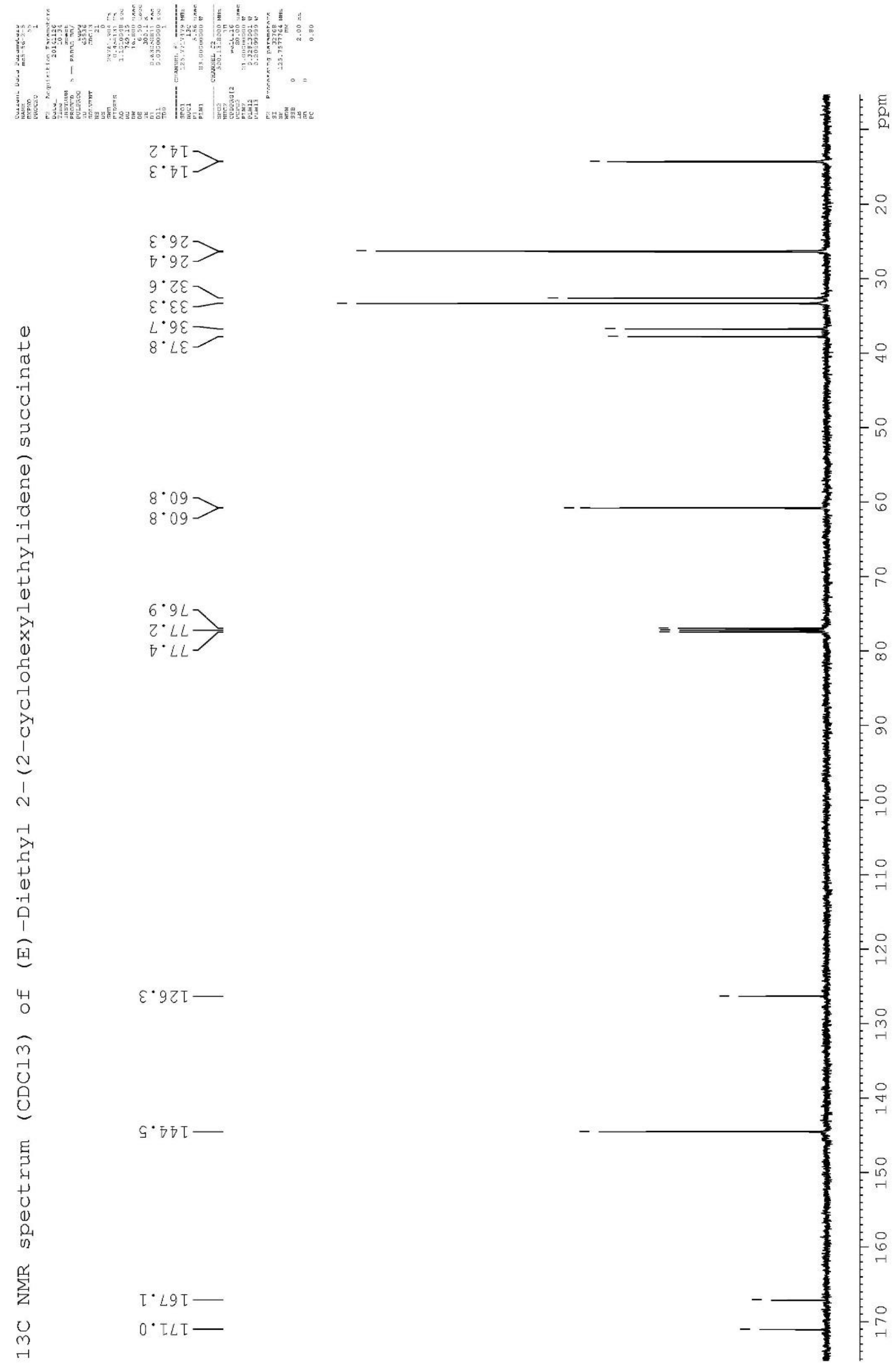

Figure 26 ^{13}C NMR spectrum in $CDCl_3$ (125 MHz) of (*E*)-diethyl 2-(2-cyclohexylethylidene)succinate (**25**).

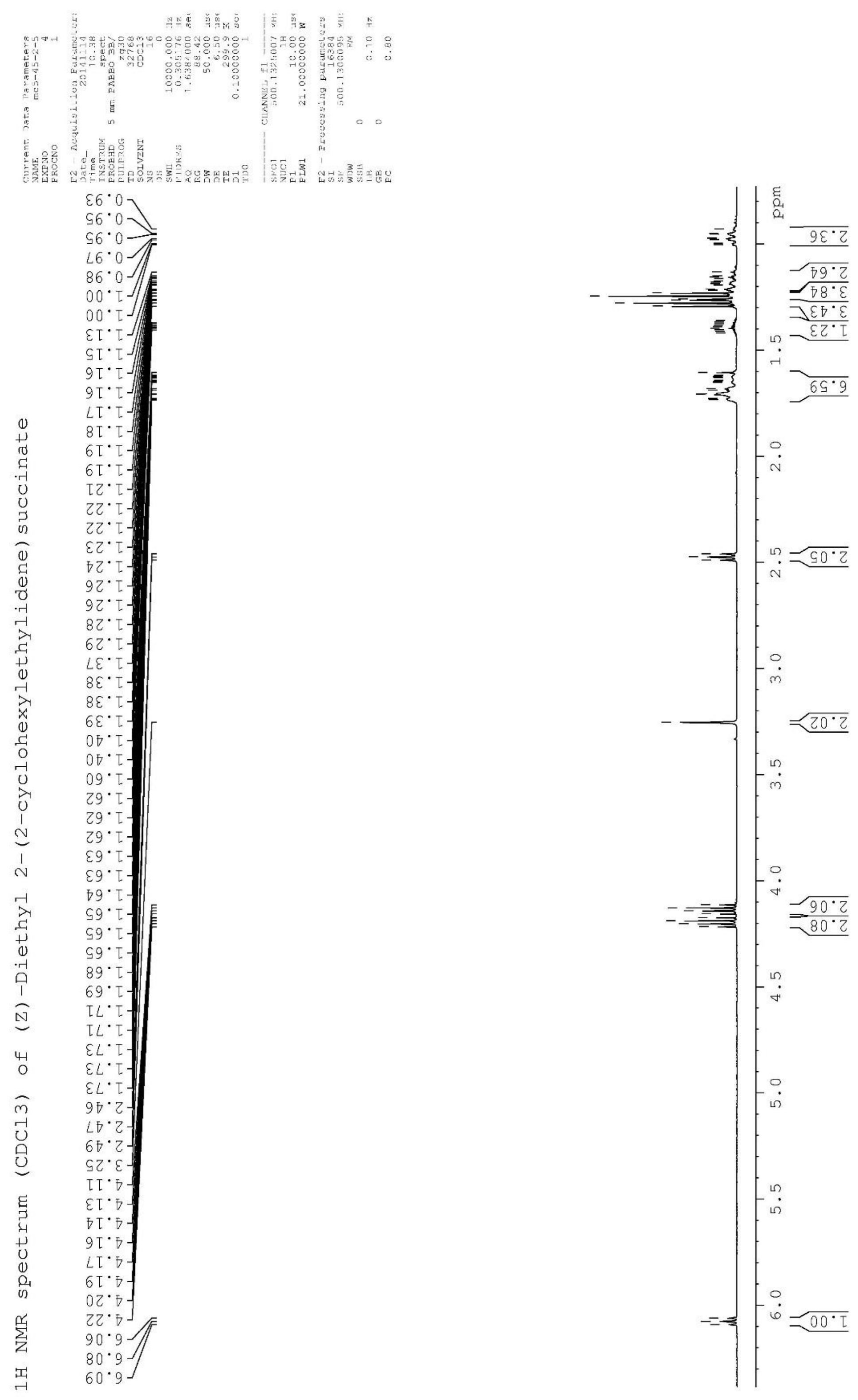

Figure 27 ^{1}H NMR spectrum in $CDCl_3$ (500 MHz) of (*Z*)-diethyl 2-(2-cyclohexylethylidene)succinate (**26**).

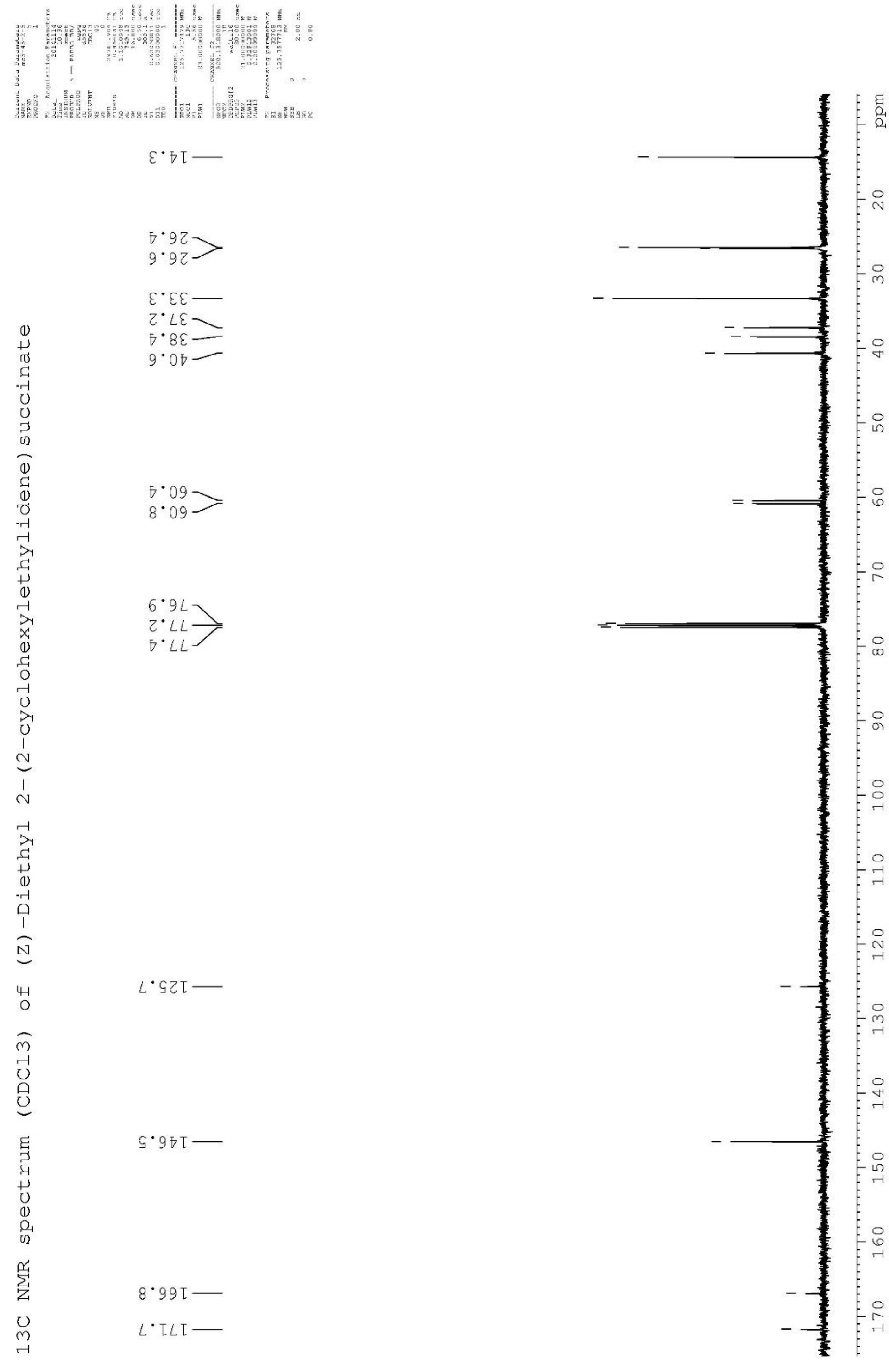

Figure 28 ^{13}C NMR spectrum in $CDCl_3$ (125 MHz) of (*Z*)-diethyl 2-(2-cyclohexylethylidene)succinate (**26**).

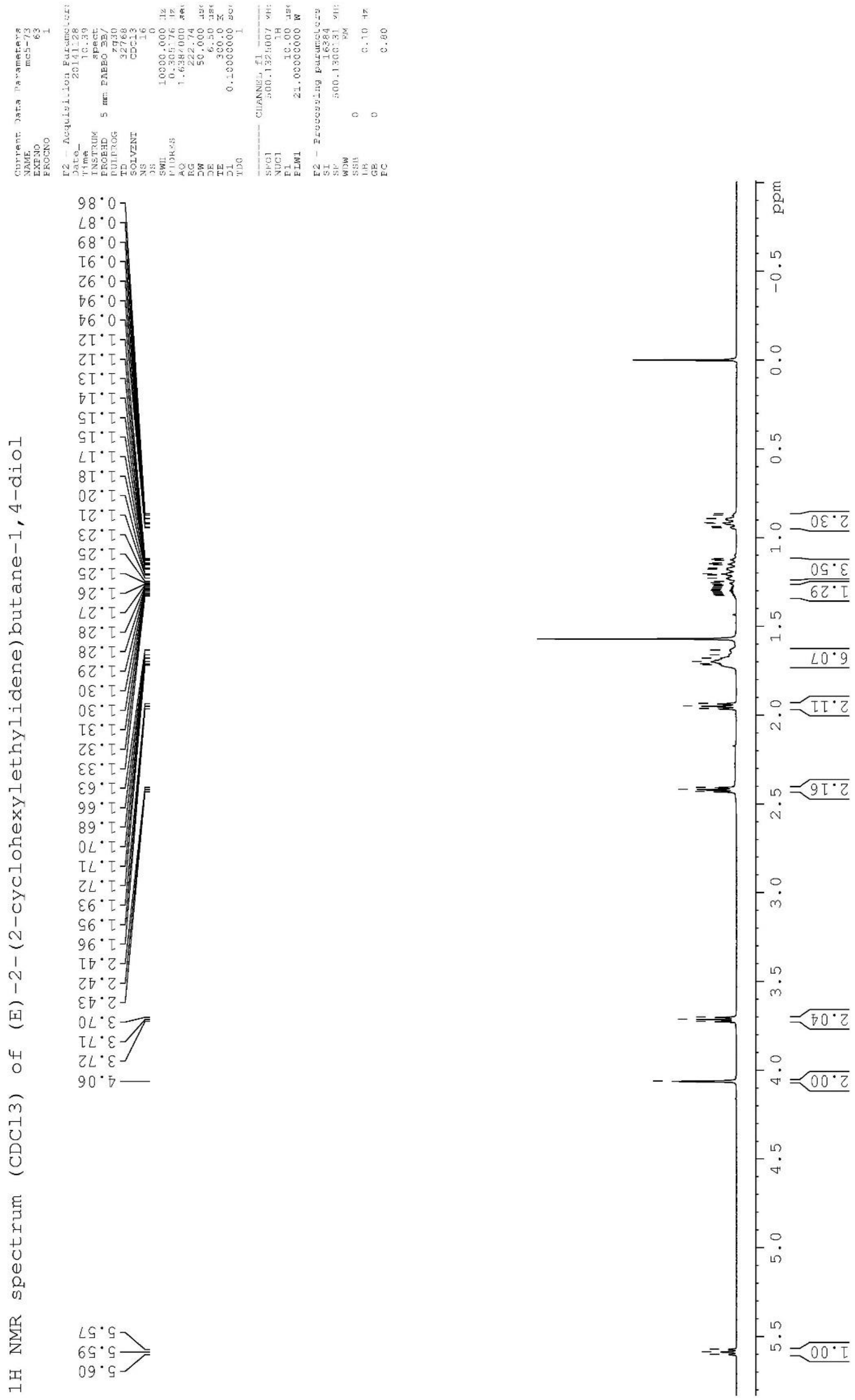

Figure 29 ^{1}H NMR spectrum in $CDCl_3$ (500 MHz) of (*E*)-2-(2-cyclohexylethylidene)butane-1,4-diol (**27**).

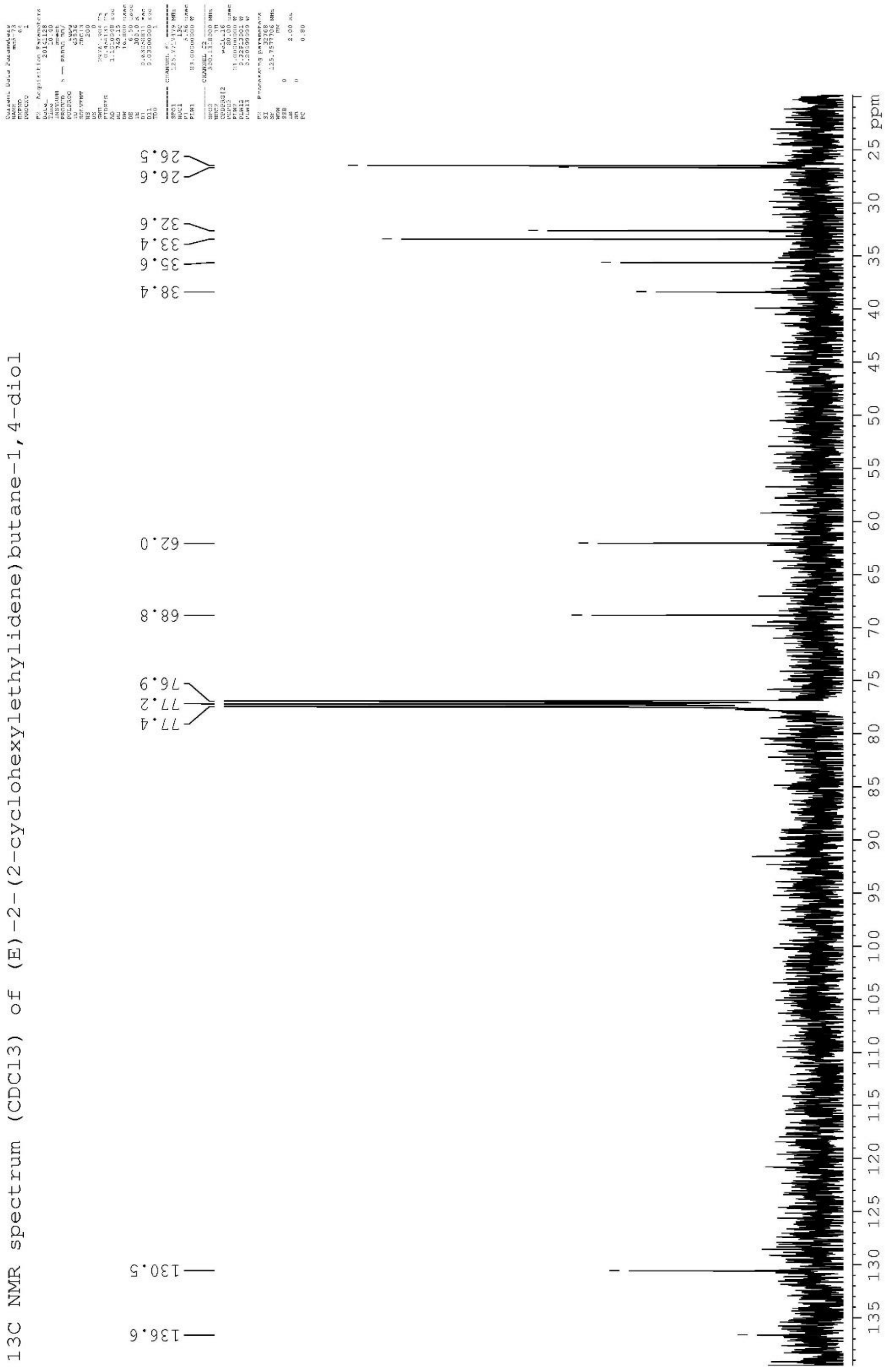

Figure 30 ^{13}C NMR spectrum in $CDCl_3$ (125 MHz) of (*E*)-2-(2-cyclohexylethylidene)butane-1,4-diol (**27**).

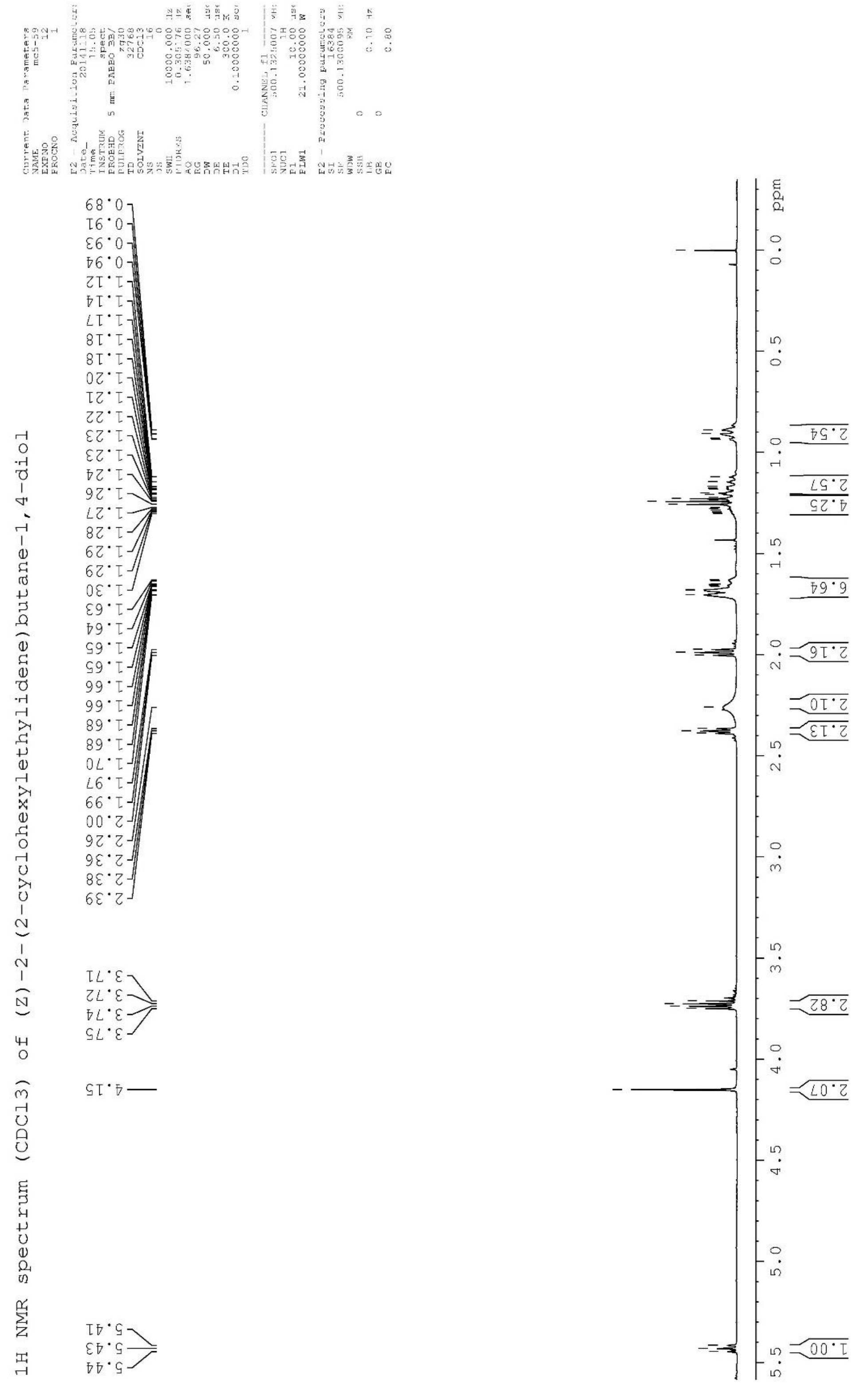

Figure 31 ^{1}H NMR spectrum in $CDCl_3$ (500 MHz) of (*Z*)-2-(2-cyclohexylethylidene)butane-1,4-diol (**28**).

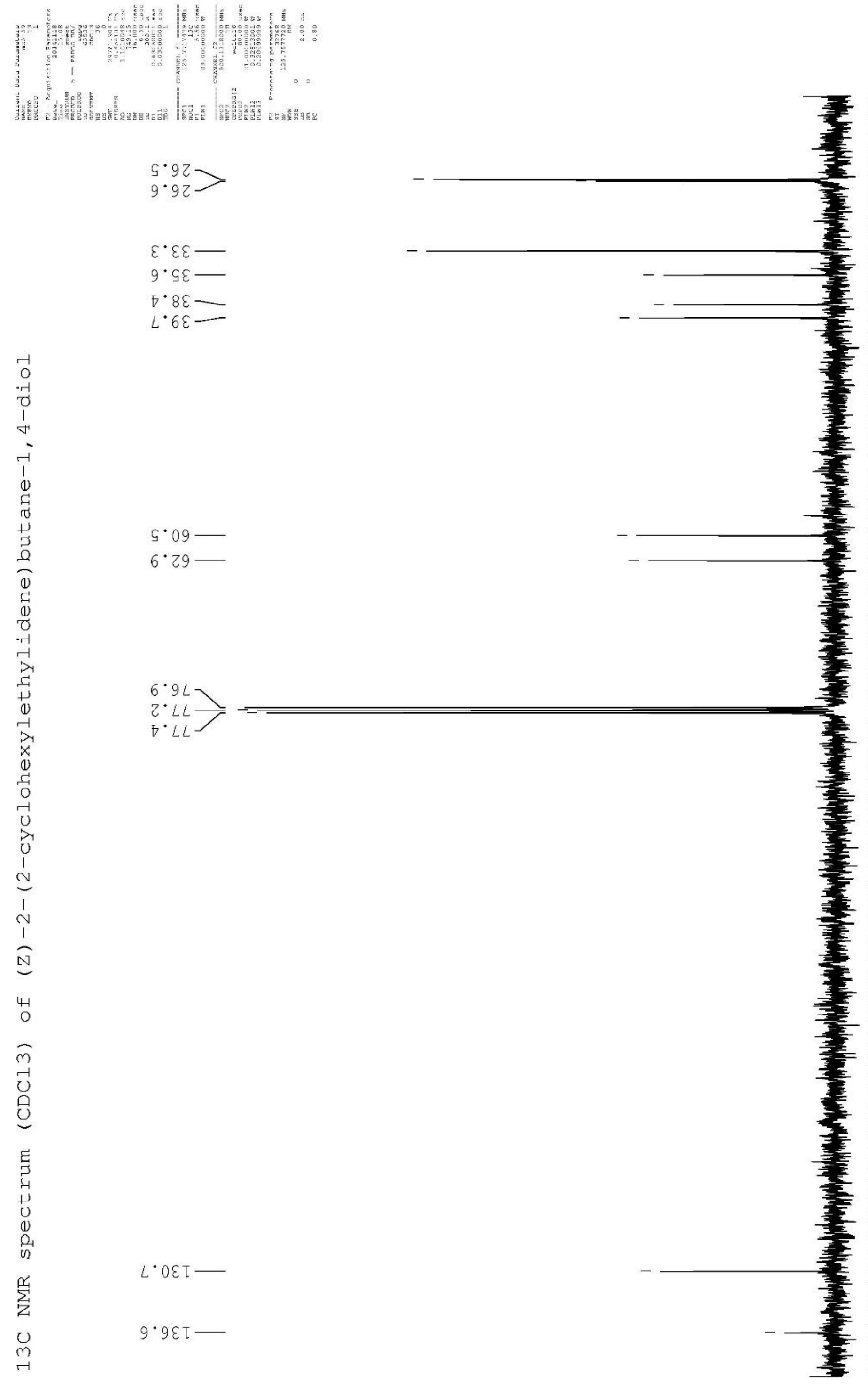

Figure 32 ^{13}C NMR spectrum in $CDCl_3$ (125 MHz) of (*Z*)-2-(2-cyclohexylethylidene)butane-1,4-diol (**28**).

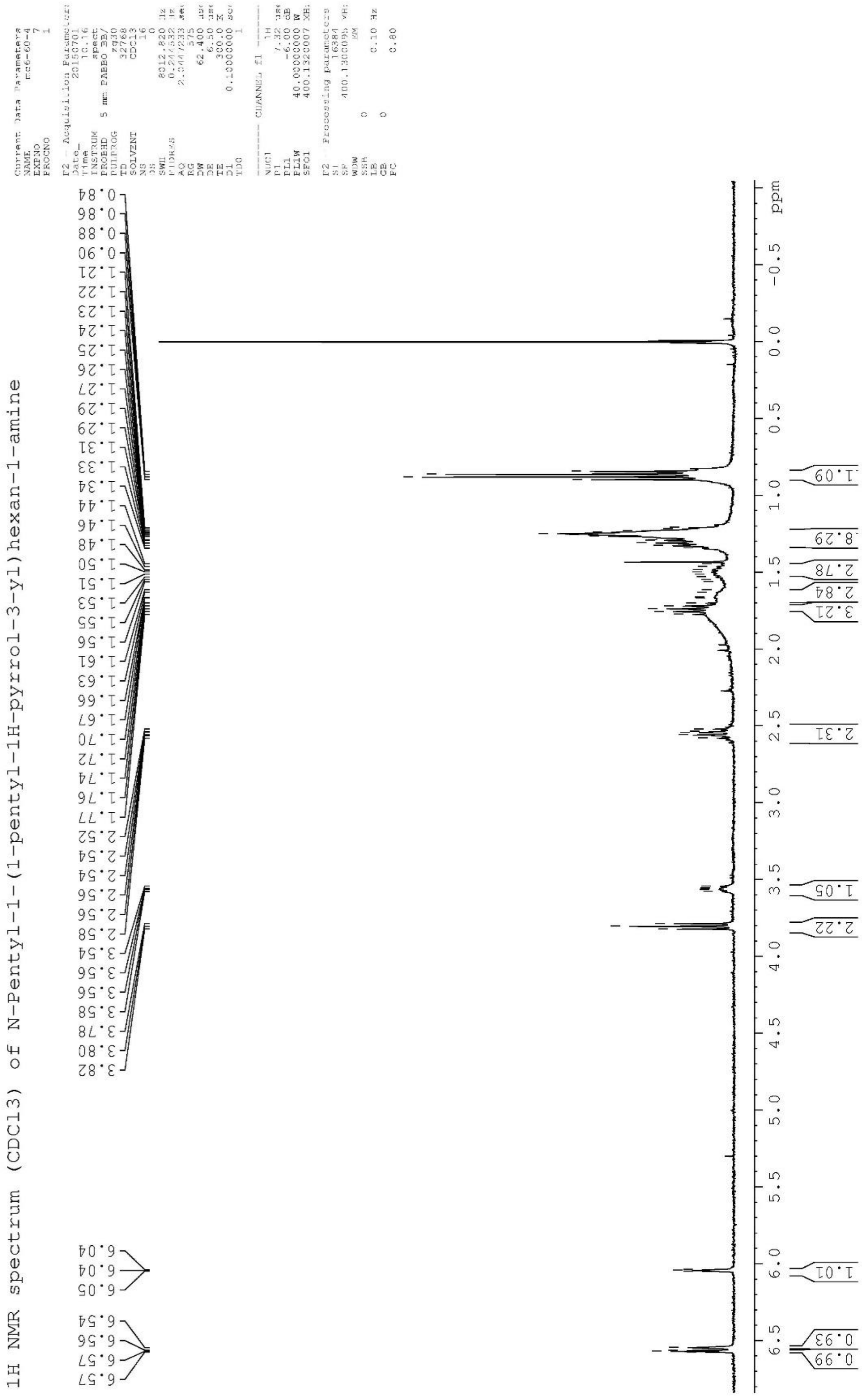

Figure 33 ^{1}H NMR spectrum in $CDCl_3$ (400 MHz) of *N*-pentyl-1-(1-pentyl-1*H*-pyrrol-3-yl)hexan-1-amine (**29**).

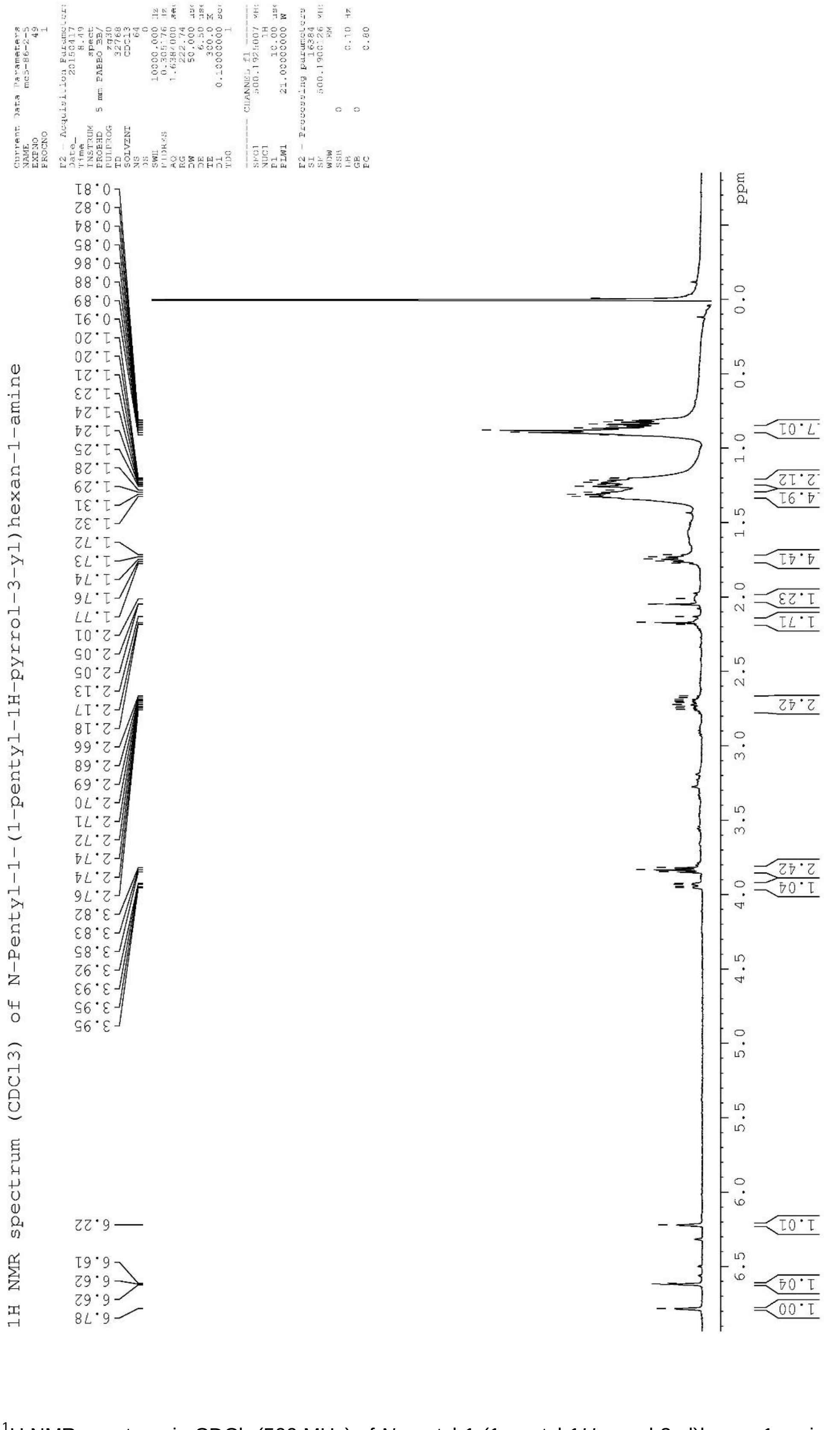

Figure 34 ^{1}H NMR spectrum in $CDCl_3$ (500 MHz) of *N*-pentyl-1-(1-pentyl-1*H*-pyrrol-3-yl)hexan-1-amine (**30**).

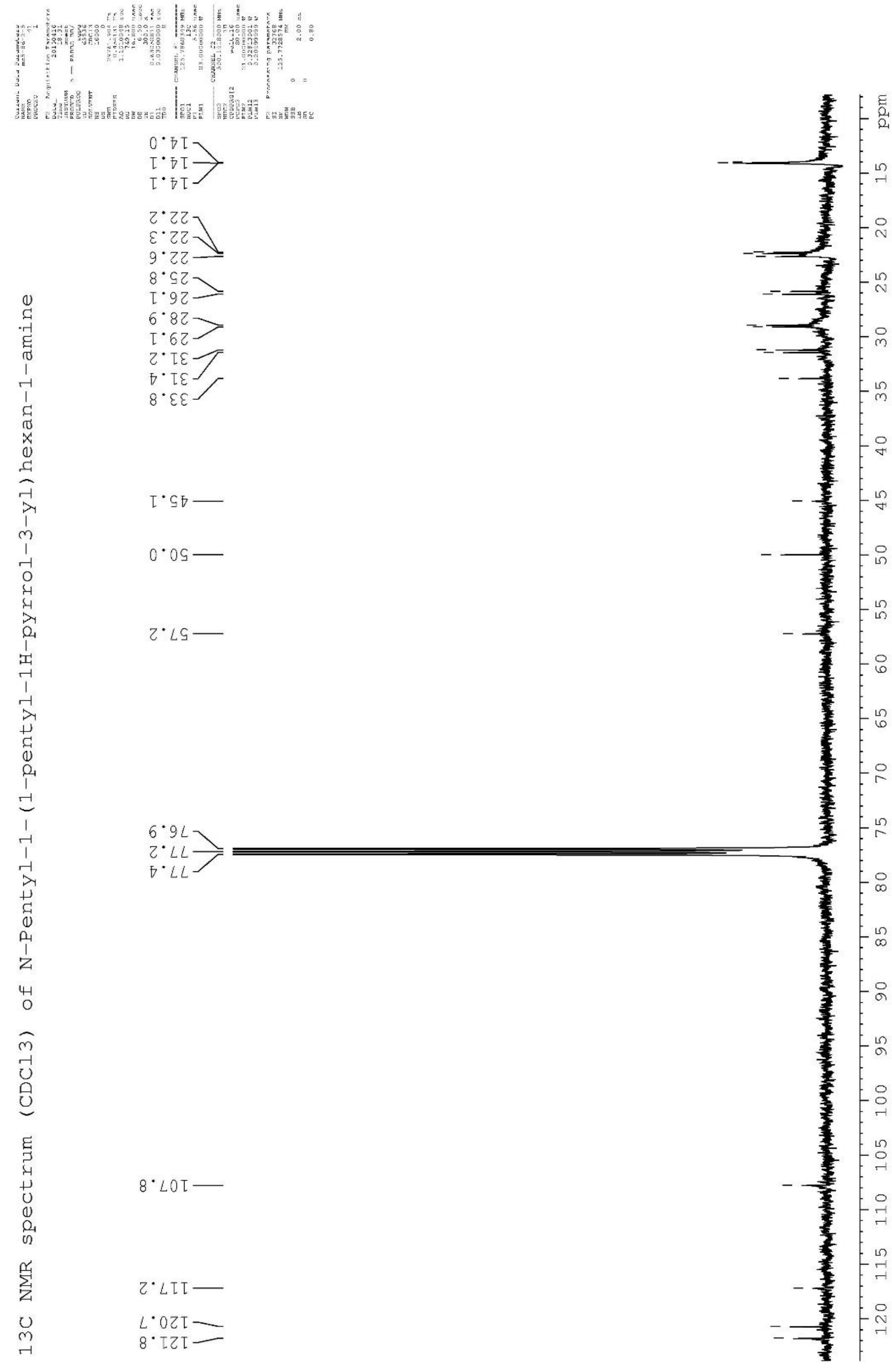

Figure 35 ^{13}C NMR spectrum in $CDCl_3$ (125 MHz) of *N*-pentyl-1-(1-pentyl-1*H*-pyrrol-3-yl)hexan-1-amine (**30**).

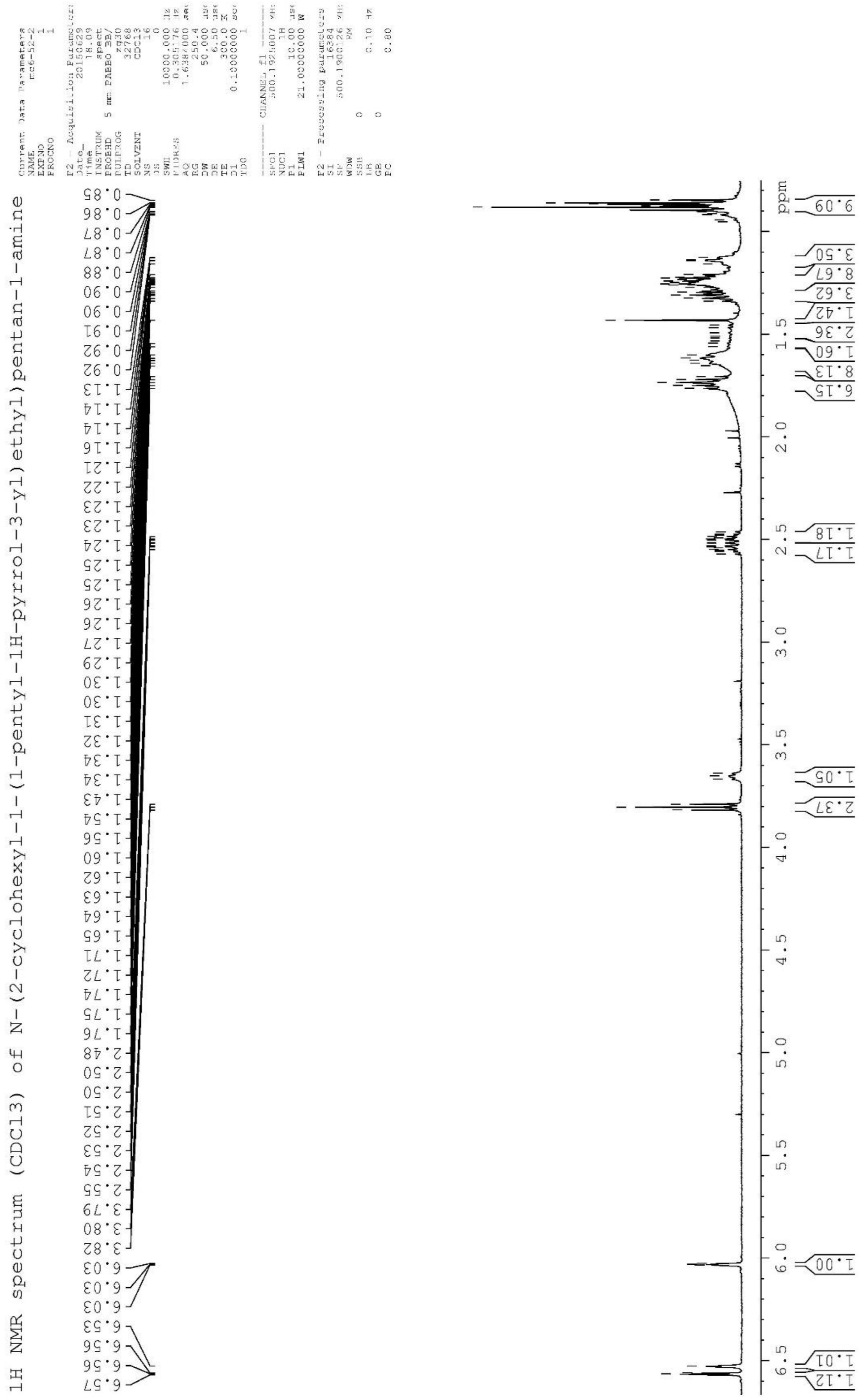

Figure 36 ^{1}H NMR spectrum in $CDCl_3$ (500 MHz) of *N*-(2-cyclohexyl-1-(1-pentyl-1*H*-pyrrol-3-yl)ethyl)pentan-1-amine (**31**).

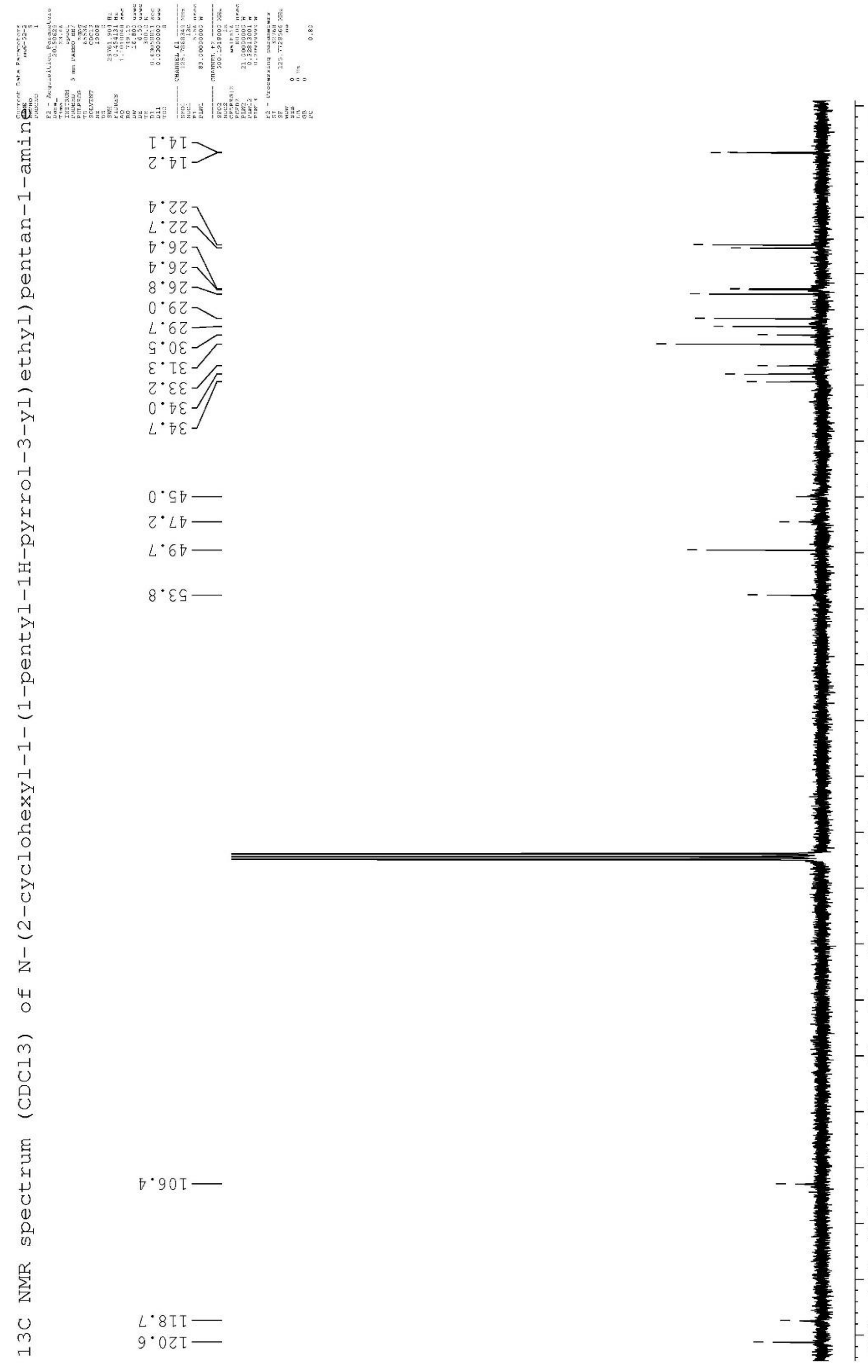

Figure 37 ^{13}C NMR spectrum in $CDCl_3$ (125 MHz) of *N*-(2-cyclohexyl-1-(1-pentyl-1*H*-pyrrol-3-yl)ethyl)pentan-1-amine (**31**).

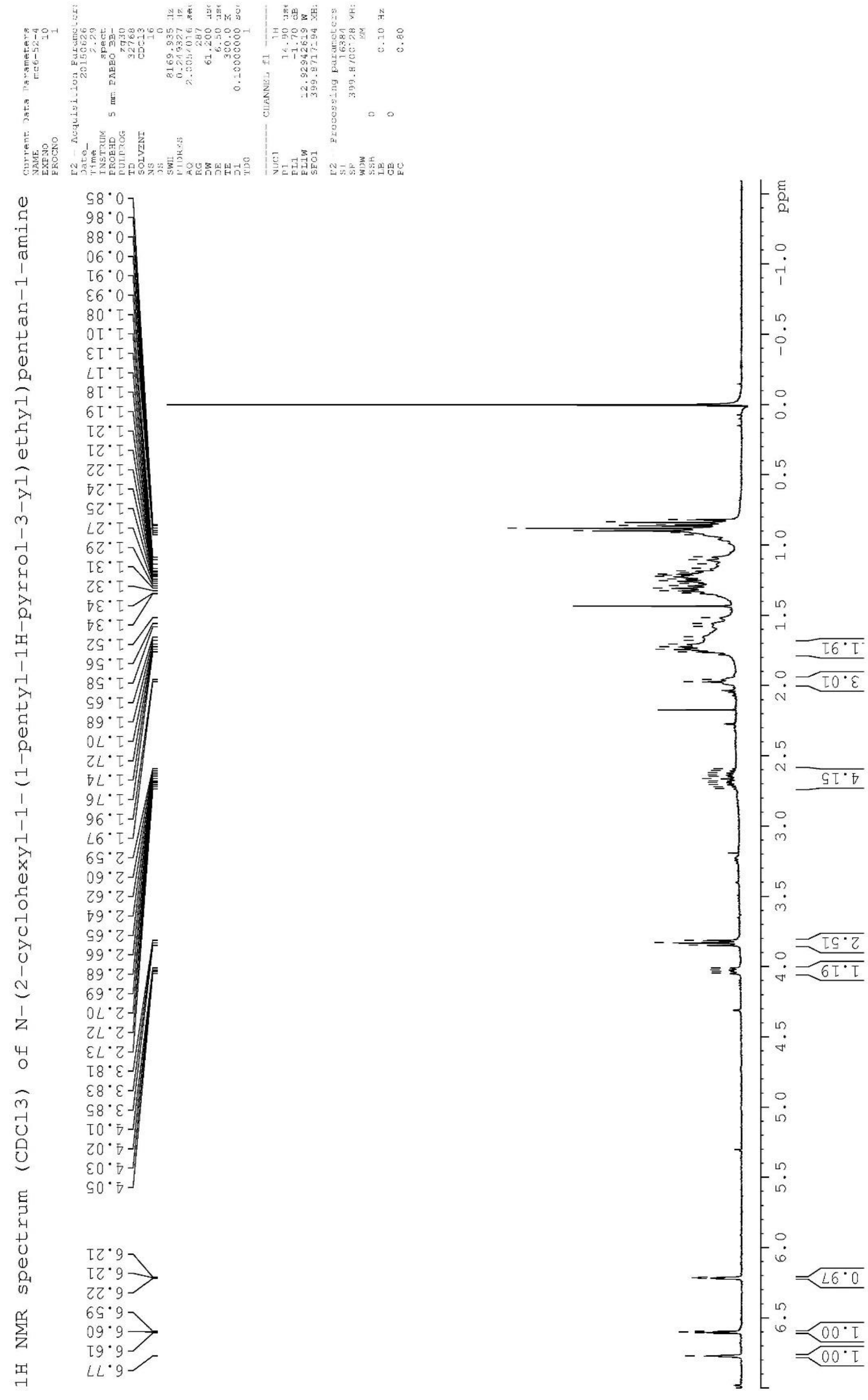

Figure 38 ^{1}H NMR spectrum in $CDCl_3$ (400 MHz) of *N*-(2-cyclohexyl-1-(1-pentyl-1*H*-pyrrol-3-yl)ethyl)pentan-1-amine (**32**).

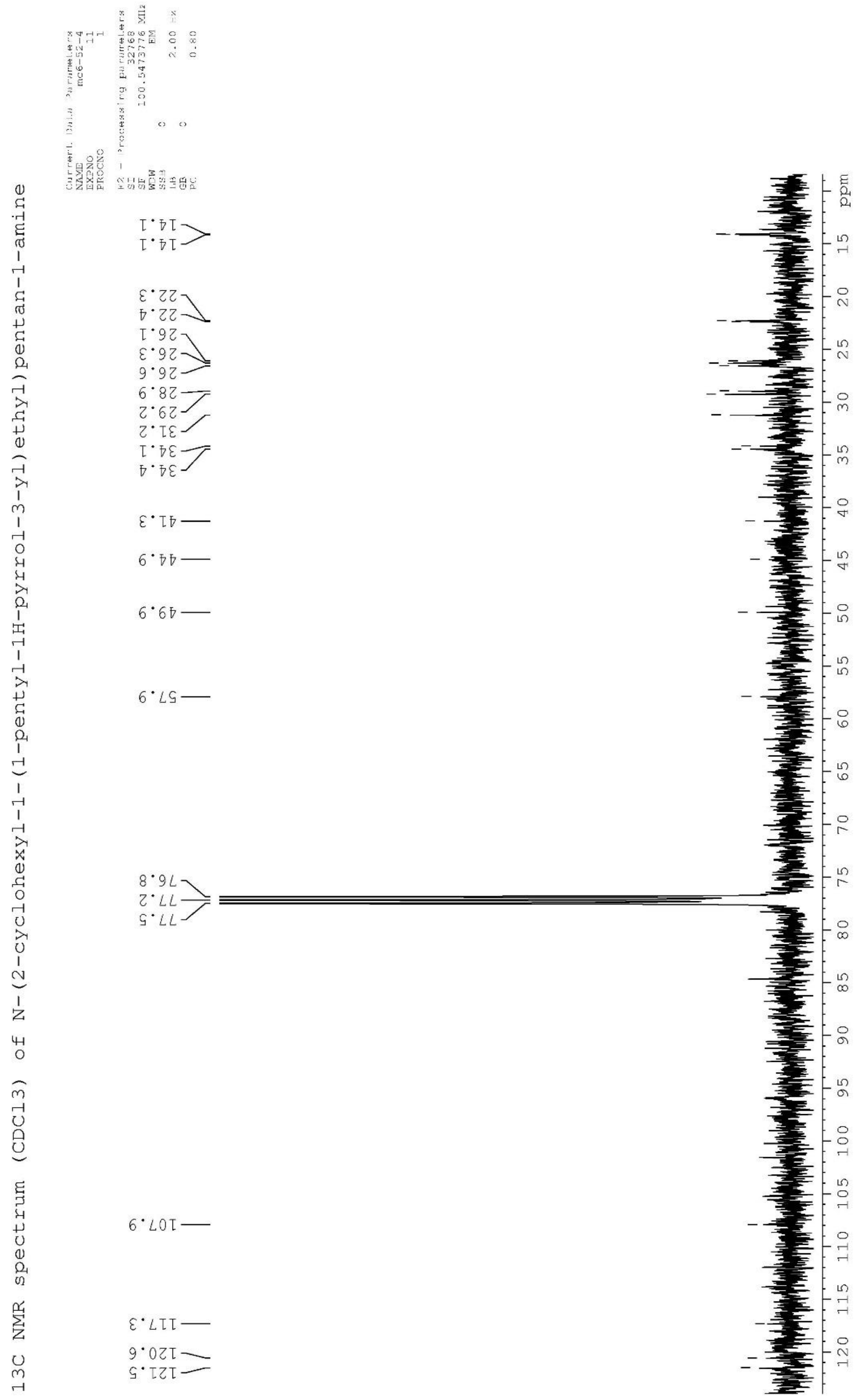

Figure 39 ^{13}C NMR spectrum in $CDCl_3$ (100 MHz) of *N*-(2-cyclohexyl-1-(1-pentyl-1*H*-pyrrol-3-yl)ethyl)pentan-1-amine (**32**).
